# Systematic review: Effects, design choices, and context of pay-for-performance in health care

**DOI:** 10.1186/1472-6963-10-247

**Published:** 2010-08-23

**Authors:** Pieter Van Herck, Delphine De Smedt, Lieven Annemans, Roy Remmen, Meredith B Rosenthal, Walter Sermeus

**Affiliations:** 1 Center for Health Services and Nursing Research, Katholieke Universiteit Leuven, Kapucijnenvoer 35, 3000 Leuven, Belgium; 2 Department of Public Health, Ghent University, De Pintelaan 185 Blok A-2, 9000 Gent, Belgium; 3 Department of General Practice, University Antwerp, Universiteitsplein 1, 2610 Wilrijk, Belgium; 4 Harvard School of Public Health, Health Policy and Management, 677 Huntington Avenue, Boston, MA 02115, USA

## Abstract

**Background:**

Pay-for-performance (P4P) is one of the primary tools used to support healthcare delivery reform. Substantial heterogeneity exists in the development and implementation of P4P in health care and its effects. This paper summarizes evidence, obtained from studies published between January 1990 and July 2009, concerning P4P effects, as well as evidence on the impact of design choices and contextual mediators on these effects. Effect domains include clinical effectiveness, access and equity, coordination and continuity, patient-centeredness, and cost-effectiveness.

**Methods:**

The systematic review made use of electronic database searching, reference screening, forward citation tracking and expert consultation. The following databases were searched: Cochrane Library, EconLit, Embase, Medline, PsychINFO, and Web of Science. Studies that evaluate P4P effects in primary care or acute hospital care medicine were included. Papers concerning other target groups or settings, having no empirical evaluation design or not complying with the P4P definition were excluded. According to study design nine validated quality appraisal tools and reporting statements were applied. Data were extracted and summarized into evidence tables independently by two reviewers.

**Results:**

One hundred twenty-eight evaluation studies provide a large body of evidence -to be interpreted with caution- concerning the effects of P4P on clinical effectiveness and equity of care. However, less evidence on the impact on coordination, continuity, patient-centeredness and cost-effectiveness was found. P4P effects can be judged to be encouraging or disappointing, depending on the primary mission of the P4P program: supporting minimal quality standards and/or boosting quality improvement. Moreover, the effects of P4P interventions varied according to design choices and characteristics of the context in which it was introduced.

Future P4P programs should (1) select and define P4P targets on the basis of baseline room for improvement, (2) make use of process and (intermediary) outcome indicators as target measures, (3) involve stakeholders and communicate information about the programs thoroughly and directly, (4) implement a uniform P4P design across payers, (5) focus on both quality improvement and achievement, and (6) distribute incentives to the individual and/or team level.

**Conclusions:**

P4P programs result in the full spectrum of possible effects for specific targets, from absent or negligible to strongly beneficial. Based on the evidence the review has provided further indications on how effect findings are likely to relate to P4P design choices and context. The provided best practice hypotheses should be tested in future research.

## Background

Research into the quality of health care has produced evidence of widespread deficits, even in countries with extensive resources for healthcare delivery[1-4]. One intervention to support quality improvement is to directly relate a proportion of the remuneration of providers to the achieved result on quality indicators. This mechanism is known as pay-for-performance (P4P). Although 'performance' is a broad concept that also includes efficiency metrics, P4P focuses on clinical effectiveness measures as a minimum, in any possible combination with other quality domains. Programs with a single focus on efficiency or productivity are not covered by the P4P concept as it is commonly applied.

Current P4P programs are heterogeneous with regard to the type of incentive, the targeted healthcare providers, the criteria for quality, etc. Several literature reviews have been published on the effects of P4P and on what works and what does not. In the summer of 2009, a meta-review performed in preparation for this paper identified 16 relevant reviews of sufficient methodological quality, according to Cochrane guidelines[5-20]. However, several new studies have been published in the last five years, which were not addressed.

Previous reviews concluded that the evidence is mixed with regard to P4P effectiveness, often finding a lack of impact or inconsistent effects. Existing reviews also reported a lack of evidence on the incidence of unintended consequences of P4P. Despite this, new programs continue to be developed around the world at an accelerated rate. This uninformed course risks producing a suboptimal level of health gain and/or wasting highly needed financial resources.

This paper presents the results of a systematic review of P4P effects and requisite conditions based on peer-reviewed evidence published prior to July 2009. The settings examined are medical practice in primary care and hospital care. The objectives are: (1) to provide an overview of how P4P affects clinical effectiveness, access and equity, coordination and continuity, patient-centeredness, and cost-effectiveness; (2) to summarize evidence-based insights about how such P4P effects are affected by the design choices made during the P4P design, implementation and evaluation process; and (3) to analyze the mediating effect on P4P effects stemming from the context in which a P4P program is introduced. Contextual mediators under review include healthcare system, payer, provider and patient characteristics.

## Methods

**Figure 1:**
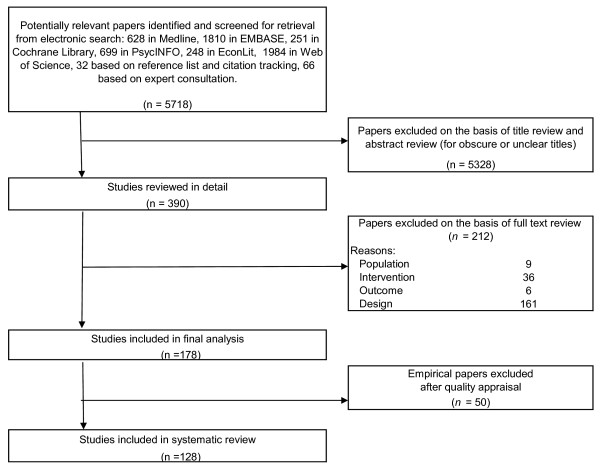
**Flow chart of search strategy, relevance screening, and quality appraisal**.

Figure 1 presents a flow chart of the methods used. Detailed information on all methodological steps can be consulted in Additional file 1. Two reviewers (DDS and PVH) independently searched for pertinent published studies, applied the inclusion and exclusion criteria to the retrieved studies, and performed quality appraisal analyses. In case of non-corresponding results, consensus was sought by consulting a third reviewer (RR). Comparison of the analysis results of the two reviewers identified 18 non-corresponding primary publications out of 5718 potentially relevant primary publications (Cohen's Kappa: 99.7%). As with previous reviews, we did not perform a meta-analysis because the selected studies had a high level of clinical heterogeneity[14].

Medline, EMBASE, the Cochrane Library, Web of Science, PsychINFO, and EconLit were searched. The concepts of quality of care, financial incentive, and a primary or acute hospital care setting were combined into a standardized search string using MeSH and non-MeSH entry terms. We limited our electronic database search to relevant studies of sufficient quality published from 2004 to 2009 or from 2005 to 2009, based on the database period covered in previous reviews. These reviews and all references of relevant publications were screened and forward citation tracking was applied without any time restriction. More than 60 international experts were asked to provide additional relevant publications.

The inclusion and exclusion criteria applied in the present review are described in Table 1.

**Table 1:** Definition of inclusion and exclusion criteria

Inclusion criteria	Exclusion criteria
Participants/Population	

Healthcare providers in primary and/or acute hospital care; being a provider organization (hospital, practice, medical group, etc.); team of providers or an individual physician	Patients as target group for financial incentives; providers in mental or behavioral health settings, or in nursing homes

Intervention	

The use of an explicit financial positive or negative incentive directly related to providers' performance with regard to specifically measured quality-of-care targets and directed at a person's income or at further investment in quality improvement; performance measured as achievement and/or improvement	The use of implicit financial incentives, which might affect quality of care but are not specifically intended to explicitly promote quality (e.g., fee-for-service, capitation, salary); the use of indirect financial incentives that affect payment only through patient attraction (e.g., public reporting)

Comparison	

No inclusion criteria specified for relevance screening	No exclusion criteria specified for relevance screening

Outcome	

At least one structural, process, or (intermediate) outcome measure on clinical effectiveness of care, access and/or equity of care, coordination and/or continuity of care, patient-centeredness, and/or cost-effectiveness of care	Subjective structural, process, or (intermediate) outcome perceptions or statements that are not measured quantitatively using a standardized validated instrument; a single focus on cost containment or productivity as targets

Design	

Primary evaluation studies published in a peer-reviewed journal or published by the Agency for Healthcare Research and Quality (AHRQ), the Institute of Medicine (IOM), the National Health Service Department of Health or a non-profit independent academic institution	Editorials, perspectives, comments, letters; papers on P4P theory, development and/or implementation without evaluation

To evaluate the study designs reported in the relevant P4P papers, we composed an appraisal tool based on nine validated appraisal tools and reporting statements: two had generic applicability,[21,22] three examined RCT design studies,[23-25] one examined cluster RCT design studies,[26] one examined interrupted time-series design studies,[27] three examined observational cohort studies,[24,28,29] and one examined cross-sectional studies[29].

For all relevant studies, we appraised ten generic items: clear description of research question, patient population and setting, intervention, comparison, effects, design, sample size, statistics, generalizability, and the addressing of confounders. If applicable, we also appraised four design-specific items: randomization, blinding, clustering effect, and number of data collection points. Modeling and cost-effectiveness studies were appraised according to guidelines from the Belgian Federal Health Care Knowledge Centre (KCE) and the International Society for Pharmacoeconomics and Outcomes Research (ISPOR)[30,31].

The following data were extracted and summarized in evidence tables: citation; country; primary versus hospital care; health system characteristics; payer characteristics; provider characteristics; patient characteristics; quality goals and targets; P4P incentives; implementing and communicating the program; quality measurement; study design; sampling, response, dropout; comparison; analysis; effectiveness evaluation; access and/or equity evaluation; coordination and/or continuity evaluation; patient-centeredness evaluation; cost-effectiveness evaluation; co-interventions; and relationship results with regard to health system, payer, provider, and patient. An overview of data extraction is provided in Additional file 2.

## Results

The first two sections provide the description of studies and the overview of effect findings, independent from design choices and context. Afterwards the latter are both specifically addressed.

### Description of studies

We identified 128 relevant evaluation studies of sufficient quality, of which 79 studies were not covered in previous review papers. In the 1990s, only a few studies were published each year. Since 2000, however, this number increased to more than 20 studies per annum in 2007 and 2008, and 18 studies in the first half of 2009. Sixty-three included studies were conducted in the USA and 57 were conducted in the UK. Two studies took place in Australia, two in Germany, and two in Spain. One P4P evaluation study from Argentina and one from Italy were published. The majority of preventive and acute care results were extracted from studies performed in the USA. A mixture of countries contributed to chronic care results. Of the 128 studies, 111 evaluated P4P use in a primary care setting, and 30 studies assessed P4P use in a hospital setting. Thirteen studies took place in both settings.

Seven study designs can be distinguished based on randomization, comparison group, and time criteria: Nine randomized studies were included. Eighteen studies used a concurrent-historic comparison design without randomization. Three studies used a concurrent comparison design. Twenty studies were based on an interrupted time-series design. Nineteen studies used a historic before-and-after design, 51 studies used a cross-sectional design, and eight studies applied economic modeling.

### Effect findings

#### Clinical effectiveness

The clinical effectiveness of P4P is presented in Additional file 3, as assessed through randomized, concurrent plus historical and time-series design studies (47 studies in total). Thirty nine of these studies, reporting a clinical effect size, are presented in Additional file 3. It provides an overview by patient group and P4P target specification (type and definition) of the main effect reported throughout these studies (positive, negative, absent or contradictory findings) and the range of the effect size in terms of percentage of change. Five of 39 studies report effects on non-incentivized quality measures, next to incentivized P4P targets.

The effects of P4P ranged from negative or absent to positive (1 to 10%) or very positive (above 10%), depending on the target and program. Negative results were found only in a minority of cases: in three studies on one target, each of which also reported positive results on other targets [32-34]. It is noteworthy that 'negative' in this context means less quality improvement compared to non P4P use and not a quality decline. In general there was about 5% improvement due to P4P use, but with a lot of variation, depending on the measure and program.

For preventive care, we found more conflicting results for screening targets than immunization targets. Across the studies, P4P most frequently failed to affect acute care. In chronic care, diabetes was the condition with the highest rates of quality improvement due to P4P implementation. Positive results were also reported for asthma and smoking cessation. This contrasts with finding no effect with regard to coronary heart disease (CHD) care. The effect of P4P on non-incentivized quality measures varied from none to positive. However, one study reported a declining trend in improvement rate for non-incentivized measures of asthma and CHD after a performance plateau was reached[35]. Finally, one study found positive effects on P4P targets concerning coronary heart disease, COPD, hypertension and stroke when applied to non-incentivized medical conditions (10.9% effect size), suggesting a spillover effect[36]. This implies a better performance on the same measures as included in a P4P program, but applied on patient groups outside off the program.

#### Access and equity of care

In addition to affecting clinical effectiveness, P4P also affects the equity of care. The body of evidence supporting this finding mainly comes from the UK. In general, P4P did not have negative effects on patients of certain age groups, ethnicity, or socio-economic status, or patients with different comorbid conditions. This finding is supported by 28 studies with a balanced utilization of cross sectional, before after, time series and concurrent comparison research designs [37-64]. In fact, throughout these studies a closing gap has been identified for performance differences. A small difference implicating less P4P achievement for female as compared to male patients was found[46,49,60,61]. One should take into account that no randomized intervention studies have reported equity of care results.

#### Coordination and continuity of care

Isolated before-and-after studies lacking control groups show that directing P4P toward the coordination of care might have positive effects[65,66]. One time-series study reported no effect on non-incentivized access and communication measures[35]. This study, however, did observe a patient self-reported decrease in timely access to patients' regular doctors, which might be a negative spillover effect[35].

#### Patient Centeredness

With regard to patient-centeredness, two studies--one Spanish before-and-after study without a control group and one cross-sectional study in the US--found no and positive P4P effects, respectively, on patient experience[67,68]. Another before-and-after study, this one from Argentina, reported that P4P had no significant effect on patient satisfaction, due to a ceiling effect[69].

#### Cost effectiveness

Concerning modeling of health gain, cost, and/or cost-effectiveness, one study found a short-term 2.5-fold return on investment for each dollar spent, as a consequence of cost savings[70]. Kahn et al (2006) report on the Premier project, a hospital P4P program in the US, not having a sufficient amount of pooled resources originating from financial penalties to cover bonus expenses[71]. Findings for health gain were varied, target specific, and baseline dependent[72-74]. Cost-effectiveness of P4P use was confirmed by four studies[75-78]. These few studies vary in methodological quality, but all report positive effects.

### Design choices

We describe the evidence on the effect of P4P design choices within a quality improvement cycle of (1) quality goals and targets, (2) quality measurement, (3) P4P incentives, (4) implementing and communicating the program, and (5) evaluation. All 128 studies are used as an input to summarize relevant findings on the effect of design choices.

#### (1) Quality goals and targets

With regard to quality goals and targets, process indicators [32,79-81] generally yielded higher improvement rates than outcome measures,[82-86] with intermediate outcome measures yielding in-between rates[35,87-90]. Whereas early programs generally addressed one patient group or focus (e.g. immunization), recent programs have expanded patient group coverage and the diversity of targets included. Still, coverage is limited to only up to ten patient groups, with each time a sub selection of measurement areas, of all groups and areas a care provider deals with.

Both underuse- and overuse-focused P4P programs can lead to positive results. However, the latter option is, at present, scarcely evaluated[57,91]. Study findings confirmed that the level of baseline performance, which defines room for improvement, influences program impact: more room for improvement enables a higher effect size[92-95]. Even though ceiling effects occur most often in acute care,[82,96] studies in this area do not typically consider such effects when selecting targets as part of P4P development.

Furthermore, studies reporting involvement of stakeholders in target selection and definition (see for example references [79,88,97,98]) seem to have found more positive P4P effects (above 10% effect size) than those that don't.

#### (2) Quality measurement

With regard to quality measurement, differences in data collection methods (chart audit, claims data, newly collected data, etc.) did not lead to substantial differences in P4P results. Both risk adjustment and exception reporting (all UK studies) seem to support P4P results when applied as part of the program. Gaming by over exception reporting and over classifying patients was kept minimal, although only three studies measured gaming specifically (e.g., 0.87% of patients exception reported wrongly)[40,43,85]. Therefore, there is limited evidence that gaming does occur with P4P use, although it is not clear what is the incidence of gaming without P4P use.

#### (3) P4P incentives

Incentives of a purely positive nature (financial rewards)[99-105] seem to have generated more positive effects than incentives based on a competitive approach (in which there are winners and losers)[33,47,81,85,92,93,95,104]. This relationship, however, is not straightforward and is clouded by the influence of other factors such as incentive size, level of stakeholder involvement, etc. The use of a fixed threshold (see for example the UK studies) versus a continuous scale to reward quality target achievement and/or improvement, are both options that resulted in positive effects in some studies (UK) but no or mixed effects in others[80,101,106]. What is clear, however, is that the positive effect was higher for initially low performers as compared to already high performers[42,64,81,92,99]. This corresponds to the above-mentioned finding regarding room for improvement. Furthermore, we found no clear relationship between incentive size and the reported P4P results. One study found a strong relationship between the program adoption rate by physicians and incentive size[100]. In this instance, the reward level, which was also determined by the number of eligible patients per provider, explained 89 to 95% of the variation in participation. Several authors in the USA indicated that a diluting effect for incentive size due to payer fragmentation likely affected the P4P results[34,100,107]. Dilution refers to the influence of providers being remunerated by multiple payers who use different incentive schemes. This results in smaller P4P patient panels per provider and lower incentive payments per provider.

With regard to the target unit of the incentive, programs aimed at the individual provider level [99,100,102,103,108] and/or team level [79,91,98] generally reported positive results. The study of Young et al. (2007)[95] is one exception. Programs aimed at the hospital level were more likely to have a smaller effect[34,92,104-106,110]. Again, these programs also showed positive results, but additional efforts seemed to be required in terms of support generation and/or internal incentive transfer. One study that examined incentives at the administrator/leadership level found mixed results[80]. A combination of incentives aimed at different target units was rarely used, but did lead to positive results[93,111].

#### (4) Implementing and communicating the program

Making new funds available for a P4P program showed positive effects (e.g. UK studies), while reallocation of funds generally showed more mixed effects [32,81,85,92].). However, as the cost-effectiveness results indicated, in the long-term, continually adding additional funding is not an option. Balancing the invested resources with estimated cost savings was applied in one study, [103] with positive results (2 to 4% effect size).

In the UK, P4P was not introduced in a stepwise fashion. As a result, subsequent nationwide corrections were required in terms of indicator selection, threshold definition and the bonus size per target[35,40,42]. Other countries have used demonstration projects to introduce P4P. At present, it is too early to tell whether the lessons learned from a phased approach will lead to a higher positive impact of P4P.

There is conflicting evidence for using voluntary versus mandatory P4P implementation. One study tested the hypothesis that voluntary schemes lead to overrepresentation of already high performers[98]. These authors found that each of the tested periods showed selection bias for only one or two of the eleven indicators tested. However, another study found significant differences between participants and non-participants in terms of hospital size, teaching status, volume of eligible patients, and mortality rate[85].

Our analysis for the present review identified communication and participant awareness of the program as important factors that affect P4P results. Several studies that found no P4P effects related their findings to an absent or insufficient awareness of the existence and the elements of a P4P program[105,106]. With regard to different communication strategies, studies found positive P4P effects (5 to 20% effect size) with programs that fostered extensive and direct communication with involved providers[87,97,106,112]. Involving all stakeholders in P4P program development also had positive effects (effect size of >10%)[79,88,97,98]. However, findings for studies examining high stakeholder involvement remain mixed[83,98].

P4P programs are often part of larger quality improvement initiatives and are therefore combined with other interventions such as feedback, education, public reporting, etc. In the UK (time-series study, 5 to 8% effect size)[35]; Spain (time-series study, above 30% effect size)[111]; and Argentina (before-and-after study, 5 to 30% effect size),[69] This arrangement seems to have reinforced the P4P effect, although one cannot be sure due to the lack of randomized studies with multiple control groups. In the USA, the impact of combined programs has been more mixed (mixed study designs, -11 to >30% effect size)[32-34,75,80,81,83,85,87,88,90,92,93,95,97-99,101-106,109,110,113-115].

#### (5) Evaluation

With regard to sustainability of change, there is evidence that a plateau of performance might be reached, with attenuation of the initial improvement rate[35].

### Context findings

We describe the influence of contextual mediators in terms of (1) healthcare system, (2) payer, (3) provider, and (4) patient characteristics on the effects of P4P.

#### (1) Healthcare system characteristics

Nation-level P4P decision making led to more uniform P4P results (as illustrated by the UK example),[35,42,64,89,116] whereas more fragmented initiatives led to more variable P4P results (as seen in the USA)[32-34,75,80,81,83,85,87,88,90,92,93,95,97-99,101-106,109,110,113-115]. The level of decision making affected many of the previously described design choices, such as the level of incentive dilution, the level of incentive awareness, etc. It is currently not clear how this also might relate to other healthcare system characteristics such as type of healthcare purchasing, degree of regulation, etc. although one could assume that such aspects also modify the level of incentive dilution.

#### (2) Payer characteristics

Theory predicts that capitation, as a dominant payment system, is associated with more underuse than overuse problems, whereas fee-for-service (FFS) is associated with more overuse than underuse problems [117]. Therefore, as a corrective incentive to complement capitation, P4P should focus more on underuse; and as a complement to FFS, it should focus more on overuse. However, the effects of P4P can be undone by the dominant payment system. A reinforcing effect is expected in instances in which the goal of both incentive structures is the same. Supportive evidence concerning these interactions is currently largely absent, due to the frequent non reporting of dominant payment system characteristics and the lack of overuse focused studies as a comparison point.

#### (3) Provider characteristics

Provider characteristics which might influence P4P effectiveness include both organizational and personal aspects. Examples of organizational aspects are leadership, culture, the history of engagement with quality improvement activities, ownership and size of the organization. Personal aspects consist of demographics, level of experience, etc. Individual provider demographics, experience, and professional background had no or a moderate effect on P4P performance[40,57,99,103,105,109,113,118-121]. In the following paragraph the influence of organizational aspects is described.

Regarding programs in which the provider is either a team or organization, one study found no relationship between the role of leadership and P4P performance[122]. Although another study found no significant association between the nature of the organizational culture and P4P performance, it did find a positive association between a patient-centered culture and P4P performance[123]. Vina et al. (2009)[122] reported a positive relationship between P4P performance effects and an organizational culture that supports the coordination of care, the perceived pace of change in the organization, the willingness to try new projects, and a focus on identifying system errors rather than blaming individuals. The history of engagement with quality improvement activities was in one study associated with positive P4P results[75]. However, another study found no relationship between P4P performance and prior readiness to meet quality standards[100]. One study found a positive relationship between P4P performance and a multidisciplinary team approach, the use of clinical pathways, and having adequate human resources for quality improvement projects[122].

Medical groups were likely to perform better than independent practice associations (IPA)[119,120,124-126]. Some studies reported a positive relationship between P4P performance and the age of the group or the age of the organization[120,127]. In the USA, ownership of the organization by a hospital or health plan was positively related to P4P performance, as compared to individual provider ownership [119,120,124,127-129].

We encountered conflicting evidence concerning the influence of the size of an organization in terms of the number of providers and number of patients. Some studies reported a positive relationship between the number of patients and P4P performance[53,119,127,130-133]. Others reported no relationship [105] or a negative relationship[37,40,109]. Some studies reported that group practices performed better on P4P than individual practices, [94,105,109] whereas one study reported the opposite[113].

Mehrotra et al. (2007)[125] found a positive relationship between P4P performance and practices that had more than the median number of physicians available, which was 39. Other studies obtained similar results[43,63,120,124,128,129]. Tahrani et al. (2008)[134] reported that the performance gap between large versus small practices in the UK disappeared after P4P implementation. Previously, small practices performed worse than large practices.

#### (4) Patient characteristics

With regard to patient characteristics, there is no available evidence on how patient awareness of P4P affects performance. Patient behavior (e.g., lifestyle, cooperation, and therapeutic compliance), however, could affect P4P results. Again, there is a lack of evidence on this specific topic, with the exception of P4P programs that specifically target long-term smoking cessation outcomes, which is more patient-lifestyle related. Results show that P4P interventions targeting smoking cessation outcomes are harder to meet[86]. The effect of other patient characteristics, such as age, gender, and socio-economical status, has been described previously (see P4P effects on equity).

## Discussion

This review analyzed published evidence on the effects of P4P reported in the recent accelerating growth of P4P literature. Previous reviews identified a lack of studies, but expected the number to increase rapidly [5,9,10,12,14,135]. The publication rate as described for the last 20 years illustrates this evolution. About two additional years have been covered as compared to previous reviews [7,13,16,17,136,137]. Seventy nine studies were not reviewed previously. One factor which likely contributed to the difference in study retrieval is the focus of some reviews on only one subset of medical conditions (e.g. prevention) [16,18,20], on one setting [13] or one study design [10]. Our review purposely focused on both primary care and hospital care without a restriction on medical condition. Furthermore, the difference in retrieval number is related to the search strategy itself. If one for example omits to include 'Quality and Outcomes Framework' (QOF), one fails to identify dozens of relevant studies.

The use of multiple study designs to investigate P4P design, implementation and effects is at present well accepted in literature [7,9,12,14]. Compared to previous reviews this paper adds the use of cross sectional survey studies to summarize contextual relations. As stated by other authors using similar quality appraisal methods, the scientific quality of the evidence available is increasing [12]. The vast majority of identified studies was not randomized (only nine were) and roughly 75 studies were either cross-sectional or employed a simple before-and-after design. However, as the evidence-base continues to grow, conclusions on the effects of P4P can increasingly be drawn with more certainty, despite the fact that the scientific quality of current evidence is still poor.

In terms of set up and reporting of P4P programs our review draws its results from studies showing an evolution from small scale, often single element, to broader programs using multidimensional quality measures, as was previously described [7,135]. Underreporting of key mediators of a P4P program remains a problem, but has recently improved for information on incentive size, frequency, etc. Combined with a larger number of studies this enables a stronger focus on the influence of design choices and on the context in which P4P results were obtained.

As is typical for health service and policy interventions, reported effects are nuanced. Magic bullets do not exist in this arena [5,7,9,12]. However, published results do show that a number of specific targets may be improved by P4P when design choices and context are optimized and aligned. Interpretation of effect size is dependent on the primary mission of P4P. When it functions to support uniform minimal standards, P4P serves its purpose in the majority of studies. If P4P is intended to boost performance of all providers, its capability to do so is confirmed for only a number of specific targets, e.g., in diabetic care.

Negative effects, in terms of less quality improvement compared to non P4P use, which were first reported in the review paper by Petersen et al (2006) [14], are rarely encountered within the 128 studies, but do occur exceptionally. Previous authors also questioned the level of gaming [7,135], and possible neglecting effects on non- incentivized quality aspects [14,135]. The presence of limited gaming is confirmed in this review, although it is only addressed in a minority of studies. Its assessment is obscured by uncertainty of the level of gaming in a non P4P context as a comparison point. As the results show, a few studies included non- incentivized measures as control variables for possible neglecting effects on non P4P quality targets. Such effects were absent in almost all of these studies The results of one study suggest the need to monitor unintended consequences further and to refine the program more swiftly and fundamentally when the target potential becomes saturated[35]. It is too early to draw firm conclusions about gaming and unintended consequences. However, based on the evidence, there may be some indications of the limited occurrence of gaming and a limited neglecting effect on non-incentivized measures. Positive spillover effects on non-incentivized medical conditions are observed in some cases, but need to be explored further[36].

Equity has not suffered under P4P implementation and is improving in the UK[37-64]. Cost-effectiveness at the population level is confirmed by the few studies available[75-78]. Further attention should be given, both in practice and research, to how P4P affects other quality domains, including access, coordination, continuity and patient-centeredness.

Previous reviews mentioned a few contextual variables which should be taken into account when running a P4P program. Examples are the influence of patient behaviour on target performance [9,10] and the difference between solo and group practices in P4P performance [12]. Our review has further contributed to the contextual framework from a health system, payer, provider and patient perspective. Program development and context findings, which related P4P effects to its design and implementation within a cyclical approach, enable us to identify preliminary P4P program recommendations. As Custers et al (2008) suggested, incentive forms are dependent on its objectives and contextual characteristics [8]. However, considering the context and goals of a P4P program, six recommendations are supported by evidence throughout the 128 studies:

1. Select and define P4P targets based on baseline room for improvement. This important condition has been overlooked in many programs, with a clear effect on results.

2. Make use of process and (intermediary) outcome indicators as target measures. See also Petersen et al (2006) and Conrad and Perry (2009), who stress that some important preconditions, including adequate risk adjustment, must be fulfilled if outcome indicators are used [14,136].

3. Involve stakeholders and communicate the program thoroughly and directly throughout development, implementation, and evaluation. The importance of awareness was already stated previously [5,7,12,14].

4. Implement a uniform P4P design across payers. If not, program effects risk to be diluted [5,6,14,135]. However, one should be cautious for anti-trust issues.

5. Focus on quality improvement and achievement, as also recommended by Petersen et al (2006) [14]. The evidence shows that both may be effective when developed appropriately. A combination of both is most likely to support acceptance and to direct the incentive to both low and high performing providers.

6. Distribute incentives at the individual level and/or at the team level. Previous reviews disagreed on the P4P target level. As in our review, some authors listed evidence on the importance of incentivizing providers individually [5,14]. Others questioned this, because of two arguments: First, the enabling role at a higher (institutional) level which controls the level of support and resources provided to the individual [9,14]. Secondly, having a sufficiently large patient panel as a sample size per target to ensure measurement reliability [136]. Our review confirms that targeting the individual has generally better effects than not to do so. A similar observation was made for incentives provided at a team level. Statistical objections become obsolete when following a uniform approach (see recommendation 4).

The following recommendations are theory based but at present show absent evidence (no. 1) or conflicting evidence (no. 2 and 3):

1. Timely refocus the programs when goals are fulfilled, but keep monitoring scores on old targets to see if achieved results are preserved.

2. Support participation and program effectiveness by means of a sufficient incentive size. As noted by other authors, there is an urgent need for further research on the dose-response relationship in P4P programs [5,7,9,10,12-14,135,136]. This is especially important, because although no clear cut relation of incentive size and effect has been established, many P4P programs in the US make use of a remarkably low incentive size (mostly 1 to 2% of income) [20,135,136]. Conflicting evidence does not justify the use of any incentive size, while still expecting P4P programs to deliver results.

3. Provide quality improvement support to participants through staff, infrastructure, team functioning, and use of quality improvement tools. See also Conrad and Perry (2009)[136].

More recent studies, after the time frame of our review, have confirmed our findings (e.g. to focus on individual providers as target unit [138]) or provided new insights. Chung et al (2010) reported in one randomized study that the frequency of P4P payment, quarterly versus yearly, does not impact performance[139]. These studies illustrate that a regular update of reviewing P4P effects and mediators is necessary.

Some limitations apply to the review results. First, there were restrictions in the search strategy used (e.g. number of databases consulted). Secondly, although quality appraisal was performed, and most studies controlled for potential confounders, selection bias cannot be ruled out with regard to observational findings. However, an analysis of randomized studies identified the same effect findings, when assessed on a RCT only inclusion basis.

Thirdly, publication bias is likely to impact the evidence-base of P4P effectiveness and data quality bias may make the comparison of results across P4P programs problematic[140].

Fourthly, behavioural health care and nursing home care were excluded from the study as potential settings for P4P application.

Fifthly, the large degree of voluntary participation in P4P programs might lead to a self selection of higher performing providers with less room for improvement (ceiling effect). This could induce an underestimation of P4P effectiveness in the general population of providers.

Finally, P4P introduces one type of financial incentive, but does not act in isolation. Other interventions are often simultaneously introduced alongside a P4P program, which might lead to an overestimation of effects. Furthermore, the fit with non financial and other payment incentives, each with their own objectives, could be leveraged in a more coordinated fashion [6,8,12,14]. Current P4P studies only provide some pieces of this more complex puzzle.

## Conclusions

Based on a quickly growing number of studies this review has confirmed previous findings with regard to P4P effects, design choices and context. New effect findings include the increasing support for positive equity and cost effectiveness effects and preliminary indications of the minimal occurrence of unintended consequences. The effectiveness of P4P programs implemented to date is highly variable, from negative (rarely) or absent to positive or very positive. The review provides further indications that programs can improve the quality of care, when optimally designed and aligned with context. To maximize the probability of success programs should take into account recommendations such as attention for baseline room for improvement and targeting at least the individual provider level.

Future research should address the issues where evidence is absent or conflicting. An unknown dose response relationship, mediated by other factors, could preclude many programs from reaching their full potential.

## Competing interests

The authors declare that they have no competing interests.

## Authors' contributions

All authors contributed to the study concept and design. PVH and DDS had full access to all of the data in the study and take responsibility for the integrity of the data and the accuracy of the data analysis. LA, RR, MBR, and WS assisted the analysis and interpretation of data. PVH provided the first draft of the manuscript. All authors read and approved the final manuscript.

## Pre-publication history

The pre-publication history for this paper can be accessed here:

http://www.biomedcentral.com/1472-6963/10/247/prepub

## Supplementary Material

Additional file 1 **Systematic review methods tables and description of included studies**. Click here for file

Additional file 2 **Overview of data extraction**. Click here for file

Additional file 3 **The effects of P4P on clinical effectiveness**. Click here for file
