# Supplementary material for: Systematic review: Effects, design choices, and context of pay-for-performance in health care
Source: BMC Health Serv Res. 2010 Aug 23;10:247. doi: 10.1186/1472-6963-10-247 (PMC2936378; doi:10.1186/1472-6963-10-247)
Supplement: Additional file 1 — Systematic review methods tables and description of included studies. [file 1472-6963-10-247-S1.DOC]

additional material: Systematic review methods tables and description of included studies

- eTable 1: Systematic reviews electronic search strategy per database 2
- eTable 2: Systematic reviews relevance screening. Rationale for exclusion based on

full text review 11

- eTable 3: Systematic reviews quality appraisal 13
- eTable 4: Primary evaluation studies electronic search strategy per database 20
- eTable 5: Primary evaluation studies relevance screening. Rationale for exclusion

based on full text review 28

- eTable 6: Primary evaluation studies quality appraisal 32
- eTable 7: Cost-effectiveness and modelling studies quality appraisal 45
- eTable 8: Description of included studies 44
- Reference list 54

**eTable 1: Systematic reviews electronic search strategy per database**

| Database | **Medline** |
| --- | --- |
| Host | <http://www.ncbi.nlm.nih.gov/sites/entrez> (Pubmed) |
| Date of search | 16/07/2009 |
| Years covered | 2000-July 2009 |
| Search Strategy | 1. (("Salaries and Fringe Benefits"[Majr] OR "Reimbursement, Incentive"[Majr] OR "Fees and Charges"[Majr] OR p4q OR p4p OR pay* OR incentive* OR compensation* OR reimbursement* OR financ* OR bonus* OR remunerat*) AND ("Treatment Outcome"[Majr] OR "Medical Errors"[Majr] OR "Quality Control"[Majr] OR "Cost-Benefit Analysis"[Majr] OR "Safety"[Majr] OR "Health Services Accessibility"[Majr] OR quality OR outcome* OR performance OR error* OR safety* OR access* OR equity OR effectiveness) AND ("Hospitals"[Majr] OR "Physicians"[Majr] OR hospital* OR physician* OR practitioner*)) AND **systematic[sb]** Limits: only items with abstracts, Humans 2. (("Salaries and Fringe Benefits"[Majr] OR "Reimbursement, Incentive"[Majr] OR "Fees and Charges"[Majr] OR p4q OR p4p OR pay* OR incentive* OR compensation* OR reimbursement* OR financ* OR bonus* OR remunerat*) AND ("Treatment Outcome"[Majr] OR "Medical Errors"[Majr] OR "Quality Control"[Majr] OR "Cost-Benefit Analysis"[Majr] OR "Safety"[Majr] OR "Health Services Accessibility"[Majr] OR quality OR outcome* OR performance OR error* OR safety* OR access* OR equity OR effectiveness) AND ("Hospitals"[Majr] OR "Physicians"[Majr] OR hospital* OR physician* OR practitioner*)) Limits: only items with abstracts, Humans, **Meta-Analysis, Practice Guideline, Review** |
| Language restrictions | none |
| Number of citations | 1813 |

**eTable 1: Systematic reviews electronic search strategy per database (Cont.)**

| Database | **Embase** |
| --- | --- |
| Host | <http://www.embase.com/home> |
| Date of search | 16/07/2009 |
| Years covered | 2000-July 2009 |
| Search Strategy | 1. ('salary and fringe benefit'/exp/mj OR 'reimbursement'/exp/mj OR 'fee'/exp/mj OR p4q OR p4p OR pay* OR incentive* OR compensation* OR reimbursement* OR financ* OR bonus* OR remunerat*) AND ('clinical effectiveness'/exp/mj OR 'medical error'/exp/mj OR 'treatment outcome'/exp/mj OR 'quality control'/exp/mj OR 'cost effectiveness analysis'/exp/mj OR 'patient safety'/exp/mj OR 'health care access'/exp/mj OR quality OR outcome* OR 'performance'/exp/mj OR error* OR safety* OR access* OR equity OR 'cost effectiveness'/exp/mj OR effectiveness) AND ('hospital'/exp/mj OR 'physician'/exp/mj OR hospital* OR physician* OR practitioner*) AND [humans]/lim AND [abstracts]/lim AND [**review**]/lim AND [embase]/lim 2. ('salary and fringe benefit'/exp/mj OR 'reimbursement'/exp/mj OR 'fee'/exp/mj OR p4q OR p4p OR pay* OR incentive* OR compensation* OR reimbursement* OR financ* OR bonus* OR remunerat*) AND ('clinical effectiveness'/exp/mj OR 'medical error'/exp/mj OR 'treatment outcome'/exp/mj OR 'quality control'/exp/mj OR 'cost effectiveness analysis'/exp/mj OR 'patient safety'/exp/mj OR 'health care access'/exp/mj OR quality OR outcome* OR 'performance'/exp/mj OR error* OR safety* OR access* OR equity OR 'cost effectiveness'/exp/mj OR effectiveness) AND ('hospital'/exp/mj OR 'physician'/exp/mj OR hospital* OR physician* OR practitioner*) AND [humans]/lim AND [abstracts]/lim AND **([cochrane review]/lim OR [meta analysis]/lim OR [systematic review]/lim)** AND [embase]/lim |
| Language restrictions | none |
| Number of citations | 1966 |

**eTable 1: Systematic reviews electronic search strategy per database (Cont.)**

| Database | **Web of science** |
| --- | --- |
| Host | <http://apps.isiknowledge.com/> |
| Date of search | 16/07/2009 |
| Years covered | 2000-July 2009 |
| Search Strategy | TS=(salary and fringe benefit OR reimbursement OR fee OR p4q OR p4p OR pay* OR incentive* OR compensation* OR reimbursement* OR financ* OR bonus* OR remunerat*) AND TS=(clinical effectiveness OR medical error OR treatment outcome OR quality control OR cost effectiveness analysis OR patient safety OR health care access OR quality OR outcome* OR performance OR error* OR safety* OR access* OR equity OR cost effectiveness OR effectiveness) AND TS=(hospital OR physician OR hospital* OR physician* OR practitioner*) AND Document Type=(Review) |
| Language restrictions | none |
| Number of citations | 476 |

**eTable 1: Systematic reviews electronic search strategy per database (Cont.)**

| Database | **Centre for Reviews and Dissemination** |
| --- | --- |
| Host | <http://www.york.ac.uk/inst/crd/> |
| Date of search | 16/07/2009 |
| Years covered | 2000-July 2009 |
| Search Strategy | TS=(salary and fringe benefit OR reimbursement OR fee OR p4q OR p4p OR pay* OR incentive* OR compensation* OR reimbursement* OR financ* OR bonus* OR remunerat*) AND TS=(clinical effectiveness OR medical error OR treatment outcome OR quality control OR cost effectiveness analysis OR patient safety OR health care access OR quality OR outcome* OR performance OR error* OR safety* OR access* OR equity OR cost effectiveness OR effectiveness) AND TS=(hospital OR physician OR hospital* OR physician* OR practitioner*) AND Document Type=(Review)  Further selection of DARE and HTA results. |
| Language restrictions | none |
| Number of citations | 457 |

**eTable 1: Systematic reviews electronic search strategy per database (Cont.)**

| Database | **Cochrane Library** |
| --- | --- |
| Host | <http://www3.interscience.wiley.com/cgi-bin/mrwhome/106568753/HOME?CRETRY=1&SRETRY=0> |
| Date of search | 16/07/2009 |
| Years covered | 2000-July 2009 |
| Search Strategy | | #1 | [MeSH descriptor **Salaries and Fringe Benefits** explode all trees](http://www3.interscience.wiley.com/cochrane/searchHistory?mode=runquery&qnum=1) | 561 | | --- | --- | --- | | #2 | [MeSH descriptor **Reimbursement, Incentive** explode all trees](http://www3.interscience.wiley.com/cochrane/searchHistory?mode=runquery&qnum=2) | 48 | | #3 | [MeSH descriptor **Fees and Charges** explode all trees](http://www3.interscience.wiley.com/cochrane/searchHistory?mode=runquery&qnum=3) | 1079 | | #4 | [p4q](http://www3.interscience.wiley.com/cochrane/searchHistory?mode=runquery&qnum=4) | 0 | | #5 | [p4p](http://www3.interscience.wiley.com/cochrane/searchHistory?mode=runquery&qnum=5) | 2 | | #6 | [pay*](http://www3.interscience.wiley.com/cochrane/searchHistory?mode=runquery&qnum=6) | 5006 | | #7 | [incentive*](http://www3.interscience.wiley.com/cochrane/searchHistory?mode=runquery&qnum=7) | 1146 | | #8 | [compensation*](http://www3.interscience.wiley.com/cochrane/searchHistory?mode=runquery&qnum=8) | 862 | | #9 | [reimbursement*](http://www3.interscience.wiley.com/cochrane/searchHistory?mode=runquery&qnum=9) | 1571 | | #10 | [financ*](http://www3.interscience.wiley.com/cochrane/searchHistory?mode=runquery&qnum=10) | 4446 | | #11 | [bonus*](http://www3.interscience.wiley.com/cochrane/searchHistory?mode=runquery&qnum=11) | 50 | | #12 | [remunerat*](http://www3.interscience.wiley.com/cochrane/searchHistory?mode=runquery&qnum=12) | 83 | | #13 | [(#1 OR #2 OR #3 OR #4 OR #5 OR #6 OR #7 OR #8 OR #9 OR #10 OR #11 OR #12)](http://www3.interscience.wiley.com/cochrane/searchHistory?mode=runquery&qnum=13) | 12435 | | #14 | [MeSH descriptor **Treatment Outcome** explode all trees](http://www3.interscience.wiley.com/cochrane/searchHistory?mode=runquery&qnum=14) | 58725 | | #15 | [MeSH descriptor **Medical Errors** explode all trees](http://www3.interscience.wiley.com/cochrane/searchHistory?mode=runquery&qnum=15) | 1720 | | #16 | [MeSH descriptor **Quality Control** explode all trees](http://www3.interscience.wiley.com/cochrane/searchHistory?mode=runquery&qnum=16) | 382 | | #17 | [MeSH descriptor **Cost-Benefit Analysis** explode all trees](http://www3.interscience.wiley.com/cochrane/searchHistory?mode=runquery&qnum=17) | 14751 | | #18 | [MeSH descriptor **Safety** explode all trees](http://www3.interscience.wiley.com/cochrane/searchHistory?mode=runquery&qnum=18) | 0 | | #19 | [MeSH descriptor **Health Services Accessibility** explode all trees](http://www3.interscience.wiley.com/cochrane/searchHistory?mode=runquery&qnum=19) | 979 | | #20 | [quality](http://www3.interscience.wiley.com/cochrane/searchHistory?mode=runquery&qnum=20) | 50940 | | #21 | [outcome*](http://www3.interscience.wiley.com/cochrane/searchHistory?mode=runquery&qnum=21) | 124378 | | #22 | [performance](http://www3.interscience.wiley.com/cochrane/searchHistory?mode=runquery&qnum=22) | 29573 | | #23 | [error*](http://www3.interscience.wiley.com/cochrane/searchHistory?mode=runquery&qnum=23) | 10574 | | #24 | [safety*](http://www3.interscience.wiley.com/cochrane/searchHistory?mode=runquery&qnum=24) | 42639 | | #25 | [access*](http://www3.interscience.wiley.com/cochrane/searchHistory?mode=runquery&qnum=25) | 389266 | | #26 | [equity](http://www3.interscience.wiley.com/cochrane/searchHistory?mode=runquery&qnum=26) | 195 | | #27 | [cost effectiveness](http://www3.interscience.wiley.com/cochrane/searchHistory?mode=runquery&qnum=27) | 18992 | | #28 | [effectiveness](http://www3.interscience.wiley.com/cochrane/searchHistory?mode=runquery&qnum=28) | 47031 | | #29 | [(#14 OR #15 OR #16 OR #17 OR #18 OR #19 OR #20 OR #21 OR #22 OR #23 OR #24 OR #25 OR #26 OR #27 OR #28)](http://www3.interscience.wiley.com/cochrane/searchHistory?mode=runquery&qnum=29) | 453881 | | #30 | [MeSH descriptor **Hospitals** explode all trees](http://www3.interscience.wiley.com/cochrane/searchHistory?mode=runquery&qnum=30) | 2913 | | #31 | [MeSH descriptor **Physicians** explode all trees](http://www3.interscience.wiley.com/cochrane/searchHistory?mode=runquery&qnum=31) | 847 | | #32 | [hospital*](http://www3.interscience.wiley.com/cochrane/searchHistory?mode=runquery&qnum=32) | 122116 | | #33 | [physician*](http://www3.interscience.wiley.com/cochrane/searchHistory?mode=runquery&qnum=33) | 17387 | | #34 | [practitioner*](http://www3.interscience.wiley.com/cochrane/searchHistory?mode=runquery&qnum=34) | 6203 | | #35 | [(#30 OR #31 OR #32 OR #33 OR #34)](http://www3.interscience.wiley.com/cochrane/searchHistory?mode=runquery&qnum=35) | 135710 | | #36 | [(#13 AND #29 AND #35), from 2000 to 2009](http://www3.interscience.wiley.com/cochrane/searchHistory?mode=runquery&qnum=36) | 4022 |   Further selection of Cochrane reviews and Other review results |
| Language restrictions | none |
| Number of citations | 1116 |

**eTable 1: Systematic reviews electronic search strategy per database (Cont.)**

| Database | **PsycINFO** |
| --- | --- |
| Host | <http://ovidsp.tx.ovid.com/spa/ovidweb.cgi?T=JS&D=psyh&PAGE=main> |
| Date of search | 16/07/2009 |
| Years covered | 2000-July 2009 |
| Search Strategy | 1. exp salaries/ or exp bonuses/ or exp employee benefits/ or exp professional fees/  2. incentives/ or exp monetary incentives/ or exp "awards (merit)"/ or exp rewards/  3. p4p.mp. [mp=title, abstract, heading word, table of contents, key concepts]  4. p4q.mp. [mp=title, abstract, heading word, table of contents, key concepts]  5. pay*.mp. [mp=title, abstract, heading word, table of contents, key concepts]  6. incentive*.mp. [mp=title, abstract, heading word, table of contents, key concepts]  7. compensation*.mp. [mp=title, abstract, heading word, table of contents, key concepts]  8. reimbursement*.mp. [mp=title, abstract, heading word, table of contents, key concepts]  9. bonus*.mp. [mp=title, abstract, heading word, table of contents, key concepts]  10. remunerat*.mp. [mp=title, abstract, heading word, table of contents, key concepts]  11. exp Treatment Effectiveness Evaluation/  12. exp "Quality of Care"/  13. exp Errors/  14. exp Treatment Outcomes/  15. exp Quality Control/  16. exp "Costs and Cost Analysis"/  17. exp Safety/  18. exp health disparities/  19. quality.mp. [mp=title, abstract, heading word, table of contents, key concepts]  20. outcome*.mp. [mp=title, abstract, heading word, table of contents, key concepts]  21. error*.mp. [mp=title, abstract, heading word, table of contents, key concepts]  22. safety*.mp. [mp=title, abstract, heading word, table of contents, key concepts]  23. access*.mp. [mp=title, abstract, heading word, table of contents, key concepts]  24. equity.mp. [mp=title, abstract, heading word, table of contents, key concepts]  25. effectiveness.mp. [mp=title, abstract, heading word, table of contents, key concepts]  26. exp Hospitals/  27. exp Physicians/  28. exp Health Care Services/  29. hospital*.mp. [mp=title, abstract, heading word, table of contents, key concepts]  30. physician*.mp. [mp=title, abstract, heading word, table of contents, key concepts]  31. practitioner*.mp. [mp=title, abstract, heading word, table of contents, key concepts]  32. healthcare.mp. [mp=title, abstract, heading word, table of contents, key concepts]  33. 6 or 3 or 7 or 9 or 2 or 8 or 1 or 4 or 10 or 5  34. 25 or 11 or 21 or 17 or 12 or 20 or 15 or 14 or 22 or 18 or 24 or 23 or 13 or 16 or 19  35. 27 or 32 or 28 or 30 or 26 or 31 or 29  36. 35 and 33 and 34  37. limit 36 to (human and abstracts and yr="2000 - 2009")  38. limit 37 to ("0800 literature review" or "0830 systematic review" or 1200 meta analysis) |
| Language restrictions | none |
| Number of citations | 74 |

**eTable 1: Systematic reviews electronic search strategy per database (Cont.)**

| Database | **Econlit** |
| --- | --- |
| Host | <http://csaweb110v.csa.com/> |
| Date of search | 16/07/2009 |
| Years covered | 2000-July 2009 |
| Search Strategy | ((KW=(p4q or p4p or pay*) or KW=(incentive* or compensation* or reimbursement*) or KW=(bonus* or remunerat*)) or(DE=("pay" or "salary")) or(DE=("incentive compatibility" or "bonuses"))) and((DE=("cost effectiveness" or "quality" or "safety")) or(KW=(quality or outcome* or error*) or KW=(effectiveness or safety or access*) or KW=(disparit* or equit*))) and((DE=("healthcare" or "hospital" or "physician")) or(KW=(hospital* or physician* or practitioner*) or KW=healthcare)) |
| Language restrictions | none |
| Number of citations | 377 |

**eTable 2: Systematic reviews relevance screening. Rationale for exclusion based on full text review**

| **Citation** | **Patient/ population** | **Intervention** | **Outcome** | **Design** |
| --- | --- | --- | --- | --- |
| Chen & Feldman, 2000 ^(1)^ |  | no explicit fin incentive for quality |  |  |
| Gosden et al, 2000 ^(2)^ |  | only implicit incentives in payments systems |  |  |
| Baker, 2002 ^(3)^ |  | only implicit incentives in payment systems | only cost and productivity related managed care measures, no other quality measures |  |
| Armour & Pitts, 2003 ^(4)^ |  |  | costs and productivity |  |
| Havranek et al, 2003 ^(5)^ |  |  |  | no review |
| Yavroff et al, 2003 ^(6)^ |  | no financial incentive |  |  |
| Baily, 2004 ^(7)^ |  | only implicit incentives in payment systems |  |  |
| Borenstein et al, 2004 ^(8)^ |  |  |  | no review |
| Mojica et al, 2004 ^(9)^ |  | Financial incentives aimed at patients |  |  |
| Arnold & Straus, 2005 ^(10)^ |  | about reimbursement (yes/no) as such |  |  |
| Harris et al, 2005 ^(11)^ |  | about patient compensation status |  |  |
| Shortell et al, 2005 ^(12)^ |  | Financing and incentives for quality are addressed, but not related as an intervention. Remaining two factors next to each other. |  |  |
| Chaillet et al, 2006 ^(13)^ |  | No financial incentive |  |  |
| Veloski et al, 2006 ^(14)^ |  | about feedback |  |  |
| Yen et al, 2006 ^(15)^ |  |  |  | reviews only reviews |
| Khunti et al, 2007 ^(16)^ |  |  |  | Reviews two periods for comparison as in a primary study set up |
| Sood et al, 2007 ^(17)^ |  |  | only quantity focused, without an evidence base |  |
| Akbari et al, 2008 ^(18)^ |  | only implicit incentives in payment systems |  |  |
| Bremer et al, 2008 ^(19)^ | Mental health |  |  |  |
| Chan et al, 2008 ^(20)^ | pharmacy only | no explicit quality goal |  |  |
| Glickman et al, 2008 ^(21)^ |  | specific indicator use in P4P |  |  |
| Kaestner & Guardado, 2008 ^(22)^ |  | about reimbursement level as such |  |  |
| Lu et al, 2008 ^(23)^ |  | modification of patient copayments |  |  |
| Raftery et al, 2008 ^(24)^ |  |  | aimed at research participation |  |
| Welton et al, 2008 ^(25)^ |  | no explicit fin incentive for quality goal |  | no review |
| Briesacher et al, 2009 ^(26)^ | Nursing homes |  |  |  |
| Scott, 2009 ^(27)^ |  |  |  | Reviews only reviews + 2 original publications |

**eTable 3: Systematic reviews quality appraisal**

| Citation | Research question (PICOD) | | Search strategy | | | | | Relevance selection | | | Quality appraisal | | | Data extraction | | | Studies description | | | Hetero-geneity and pooling | | Validity rating |
| --- | --- | --- | --- | --- | --- | --- | --- | --- | --- | --- | --- | --- | --- | --- | --- | --- | --- | --- | --- | --- | --- | --- |
|  |  |  | Databases | | Entry terms | | Period |  |  |  |  |  |  |  |  |  |  |  |  |  |  |  |
| Sarnoff & Rundall, 1998 ^(28)^ | Clear (O = prevention) | | Medline only, English only | | 6 terms, unclear string | | 1975-1997 | clear | | | unclear | | | unclear | | | clear | | | clear | | Insuf-ficient |
| Achat et al, 1999 ^(29)^ | Clear (O = prevention) | | Medline only, English only | | 2 terms | | 1966-1998 | unclear | | | unclear | | | unclear | | | clear | | | NA | | Insuf-ficient |
| Buchan et al, 2000 ^(30)^ | clear | | broad (7), English only | | 11 terms, but unclear string | | 1989-1999 | clear | | | unclear | | | unclear | | | unclear | | | NA | | Insuf-ficient |
| Chaix-Couturier et al, 2000 ^(31)^ | clear (broad fin incentives) | | broad (6), Engl, French | | very broad | | 1993-1999 | clear | | | clear (EPOC) | | | clear | | | clear | | | NA | | sufficient |
| Giuffrida et al, 2000 ^(32)^ | clear (target payment) | | broad (7), plus grey | | strong string | | x-1997 | clear | | | clear (EPOC) | | | clear | | | clear | | | NA | | sufficient |
| Armour et al, 2001 ^(33)^ | clear | | refers to Cochrane collaboration handbook | | | | | | | | | | | clear | | | clear | | | NA | | sufficient |
| Heffner, 2001 ^(34)^ | clear | | narrative, unsystematic review, no criteria fulfilled | | | | | | | | | | | | | | | | | | | Insuf-ficient |
| Flynn et al, 2002 ^(35)^ | clear | | no methods specified | | | | | | | | | | | | | | | | | | | Insuf-ficient |
| Stone et al, 2002 ^(36)^ | clear (O = immunization) | | three | | unclear | | x-1999 | clear | | | clear | | | clear | | | clear | | | meta regression, ok | | sufficient |
| Grol & Grimshaw, 2003 ^(37)^ | clear | | listing of other reviews, no methods specified | | | | | | | | | | | | | | | | | | | Insuf-ficient |
| Alper, 2004 ^(38)^ | clear | | narrative, unsystematic review, no criteria fulfilled | | | | | | | | | | | | | | | | | | | Insuf-ficient |
| Conrad & Christianson, 2004 ^(39)^ | clear | | no methods specified | | | | | | | | | | | | | | | | | | | Insuf-ficient |
| Czubak et al, 2004 ^(40)^ | clear | | no methods specified | | | | | | | | | | | | | | | | | | | Insuf-ficient |
| Dudley et al, 2004 ^(41)^ | clear (RCT’s) | | two, grey, ongoing | | very broad | | 1980-2003 | clear | | | | clear | | clear | | | clear | | NA | | | sufficient |
| Kane et al, 2004 ^(42)^ | clear (O = prevention) | | five | | broad | | 1966-2002 | clear | | | | clear | | clear | | | clear | | NA | | | sufficient |
| Shortell, 2004 ^(43)^ | clear | | narrative, unsystematic review, no criteria fulfilled | | | | | | | | | | | | | | | | | | | Insuf-ficient |
| Smellie & Roy, 2005 ^(44)^ |  | | no methods specified | | | | | | | | | | | | | | | | | | | Insuf-ficient |
| Sturm et al, 2007 ^(45)^ | clear (P = prescribers) | | fifteen | | strong string | | x-2003/4/5 | clear | | clear | | | | clear | | | clear | | | NA | | sufficient |
| Town et al, 2005 ^(46)^ | clear (O = prevention, RCT’s) | | four, 16nglish only | | broad | | 1966-2002 | clear | | clear | | | | clear | | | clear | | | NA | | sufficient |
| Fenter & Lewis, 2006 ^(47)^ |  | | no methods specified | | | | | | | | | | | | | | | | | | | Insuf-ficient |
| Freed & Uren, 2006 ^(48)^ | clear | | no methods specified | | | | | | | | | | | | | | | | | | | Insuf-ficient |
| McNamara, 2006 ^(49)^ | clear | | narrative, unsystematic review, no criteria fulfilled | | | | | | | | | | | | | | | | | | | Insuf-ficient |
| Petersen et al, 2006 ^(50)^ | clear | | medline only, 16nglish only | | broad | | 1980- nov 2005 | clear | | | clear | | | clear | | | clear | | NA | | | sufficient |
| Pink et al, 2006 ^(51)^ | clear | | narrative, unsystematic review, no criteria fulfilled | | | | | | | | | | | | | | | | | | | Insuf-ficient |
| Rosenthal & Frank, 2006 ^(52)^ | clear | | five | | narrow P4Q terms | | x-2003 | clear | | | clear | | | clear | | clear | | | NA | | | sufficient |
| Chien et al, 2007 ^(53)^ | clear | | narrative, unsystematic review, no criteria fulfilled | | | | | | | | | | | | | | | | | | | Insuf-ficient |
| Doran & Fullwood, 2007 ^(54)^ | clear | | narrative, unsystematic review, no criteria fulfilled | | | | | | | | | | | | | | | | | | | Insuf-ficient |
| Ensor & Weinzierl, 2007 ^(55)^ | Clear (p = low income countries) | | Medline, econlit, grey | | unclear | | unclear | unclear | | | unclear | | | clear | | clear | | | NA | | Insuf-ficient | |
| Frolich et al, 2007 ^(56)^ | clear (RCT’s) | | medline, cochrane | | broad | | 1980- june 2005 | clear | | | clear | | | clear | | clear | | | NA | | sufficient | |
| Gonzalez et al, 2007 ^(57)^ | clear | | no methods specified | | | | | | | | | | | | | | | | | | Insuf-ficient | |
| Hartig & Allison, 2007 ^(58)^ | clear | | narrative, unsystematic review, no criteria fulfilled | | | | | | | | | | | | | | | | | | Insuf-ficient | |
| Pierce et al, 2007 ^(59)^ | clear | medline only | | | narrow P4Q terms | | x- nov 2005 | unclear | | | | unclear | unclear | | unclear | | | NA | | | Insuf-ficient | |
| Scott, 2007 ^(60)^ | clear | narrative, unsystematic review, no criteria fulfilled | | | | | | | | | | | | | | | | | | | Insuf-ficient | |
| Sikka, 2007 ^(61)^ | clear | narrative, unsystematic review, no criteria fulfilled | | | | | | | | | | | | | | | | | | | Insuf-ficient | |
| Varela, 2007 ^(62)^ | clear | narrative, unsystematic review, no criteria fulfilled | | | | | | | | | | | | | | | | | | | Insuf-ficient | |
| Chopra et al, 2008 ^(63)^ | clear (SYST REV ONLY)) | four, English only | | | unclear | | 1979- sept 2006 | clear | | | | clear | clear | | clear | | | NA | | | Insuf-ficient | |
| Christianson et al, 2008 ^(64)^ | clear | five | | | broad | | x- june 2007 | clear | | | | unclear | clear | | clear | | | NA | | | Sufficient | |
| Curry et al, 2008 ^(65)^ | clear | narrative, unsystematic review, no criteria fulfilled | | | | | | | | | | | | | | | | | | | Insuf-ficient | |
| Custers et al, 2008 ^(66)^ | clear | medline, proquest, grey, English only | | | broad | | 1995- 1 may 2006 | clear | | | | unclear | unclear | | clear | | | NA | | | sufficient | |
| Hamblin, 2008 ^(67)^ | clear | No methods specified | | | | | | | | | | | | | | | | | | | Insuf-ficient | |
| Hart-Hester et al, 2008 ^(68)^ | clear | No methods specified | | | | | | | | | | | | | | | | | | | Insuf-ficient | |
| Mason, 2008 ^(69)^ | clear | nine | | | unclear | | 1992- march 2006 | clear | | | | unclear | clear | | clear | | | NA | | | Insuf-ficient | |
| Mcdonald et al, 2008 ^(70)^ | clear (P = aust, engl, new-zealand, O = access) | four | | | unclear | | 1995- mid 2007 | unclear | | | | Not done and no reporting by design or quality subgroup | unclear | | unclear | | | NA | | | Insuf-ficient | |
| Sabatino et al, 2008 ^(71)^ | Clear (O = prevention) | five | | | clear | | X – 2004 | clear | | | | clear | clear | | clear | | | NA | | | sufficient | |
| Schatz, 2008 ^(72)^ | clear (ambulatory) | medline only | | | very narrow terms | | 2006-2007 | clear | | | | unclear | clear | | clear | | | NA | | | sufficient | |
| Thomas & Rosenthal, 2008 ^(73)^ | clear | Medline and grey | | | Very narrow terms | | unclear | clear | | | | clear | unclear | | unclear | | | NA | | | Insuf-ficient | |
| Sloan & Kasper, 2008 ^(74)^ | clear | narrative, unsystematic review, no criteria fulfilled | | | | | | | | | | | | | | | | | | | Insuf-ficient | |
| Conrad & Perry, 2009 ^(75)^ | clear | No methods specified | | | | | | | | | | | | | | | | | | | Insuf-ficient | |
| Greene & Nash, 2009 ^(76)^ | clear | Medline only | | | two | | 2004-2008 | unclear | | | | unclear | unclear | | clear | | | NA | | | Insuf-ficient | |
| James & Rosenbaum, 2009 ^(77)^ | clear | No methods specified | | | | | | | | | | | | | | | | | | | Insuf-ficient | |
| Khanduja et al, 2009 ^(78)^ | clear | Medline only | | | unclear | | 2000-2008 | unclear | | | | unclear | unclear | | unclear | | | NA | | | Insuf-ficient | |
| Mehrotra et al, 2009 ^(79)^ | Clear (P = hospital) | five | | broad | | 1996-2007 | | clear | unclear | | | | ckear | | clear | | | NA | | | Sufficient | |
| Rhoads et al, 2009 ^(80)^ | clear | No methods specified | | | | | | | | | | | | | | | | | | | Insuf-ficient | |

Legend: ‘P’ = Patient population or setting; ‘O’ = Outcome; ‘NA’ = Not applicable. If more than three items were assessed as unclear or insufficient, a publication was excluded.

**eTable 4: Primary evaluation studies electronic search strategy per database**

| Database | **Medline** |
| --- | --- |
| Host | <http://www.ncbi.nlm.nih.gov/sites/entrez> (Pubmed) |
| Date of search | 16/07/2009 |
| Years covered | 2005-July 2009. The most recent, Frolich et al (2007) searched till June 2005. Custers et al (2008) and Schatz (2008) have some methodological drawbacks. |
| Search Strategy | ("Salaries and Fringe Benefits"[Majr] OR "Reimbursement, Incentive"[Majr] OR "Fees and Charges"[Majr] OR p4q OR p4p OR pay* OR incentive* OR bonus*) AND ("Treatment Outcome"[Majr] OR "Medical Errors"[Majr] OR "Quality Control"[Majr] OR "Cost-Benefit Analysis"[Majr] OR "Safety"[Majr] OR "Health Services Accessibility"[Majr] OR quality OR outcome* OR performance OR error* OR safety* OR access* OR equity OR effectiveness) AND ("Hospitals"[Majr] OR "Physicians"[Majr] OR hospital* OR physician* OR practitioner*) AND (hasabstract[text] AND ("2005/06/01"[EDat]:"2008/12/30"[EDat]) AND (Humans[Mesh]) AND (Clinical Trial[ptyp] OR Randomized Controlled Trial[ptyp] OR Case Reports[ptyp] OR Clinical Trial, Phase I[ptyp] OR Clinical Trial, Phase II[ptyp] OR Clinical Trial, Phase III[ptyp] OR Clinical Trial, Phase IV[ptyp] OR Comparative Study[ptyp] OR Controlled Clinical Trial[ptyp] OR Evaluation Studies[ptyp] OR Technical Report[ptyp] OR Validation Studies[ptyp])) |
| Language restrictions | none |
| Number of citations | 628 |

**eTable 4: Primary evaluation studies electronic search strategy per database (Cont.)**

| Database | **Embase** |
| --- | --- |
| Host | <http://www.embase.com/home> |
| Date of search | 16/07/2009 |
| Years covered | 2004-July 2009. Embase was previously searched in three systematic reviews: Chaix-Couturier et al, 2000 (broad, till 1999), Giuffrida et al, 2000 (target payments, till 1997) and Stone et al, 2002 (prevention, till 1999). The last five years are selected as a standard approach. |
| Search Strategy | ('salary and fringe benefit'/exp/mj OR 'reimbursement'/exp/mj OR 'fee'/exp/mj OR p4q OR p4p OR pay* OR incentive* OR bonus*) AND ('clinical effectiveness'/exp/mj OR 'medical error'/exp/mj OR 'treatment outcome'/exp/mj OR 'quality control'/exp/mj OR 'cost effectiveness analysis'/exp/mj OR 'patient safety'/exp/mj OR 'health care access'/exp/mj OR quality OR outcome* OR 'performance'/exp/mj OR error* OR safety* OR access* OR equity OR 'cost effectiveness'/exp/mj OR effectiveness) AND ('hospital'/exp/mj OR 'physician'/exp/mj OR hospital* OR physician* OR practitioner*) AND [humans]/lim AND [abstracts]/lim AND [embase]/lim AND [article]/lim AND [2004-2009]/py |
| Language restrictions | none |
| Number of citations | 1810 |

**eTable 4: Primary evaluation studies electronic search strategy per database (Cont.)**

| Database | **Web of science** |
| --- | --- |
| Host | <http://apps.isiknowledge.com/> |
| Date of search | 16/07/2009 |
| Years covered | 2004-July 2009. This database was most recently partially covered by Rosenthal & Frank (2006), who searched till 2003. |
| Search Strategy | TS=(salary and fringe benefit OR reimbursement OR fee OR p4q OR p4p OR pay* OR incentive* OR compensation* OR reimbursement* OR financ* OR bonus* OR remunerat*) AND TS=(clinical effectiveness OR medical error OR treatment outcome OR quality control OR cost effectiveness analysis OR patient safety OR health care access OR quality OR outcome* OR performance OR error* OR safety* OR access* OR equity OR cost effectiveness OR effectiveness) AND TS=(hospital OR physician OR hospital* OR physician* OR practitioner*) |
| Language restrictions | none |
| Number of citations | 1984 |

**eTable 4: Primary evaluation studies electronic search strategy per database (Cont.)**

| Database | **Cochrane Library** |
| --- | --- |
| Host | <http://www3.interscience.wiley.com/cgi-bin/mrwhome/106568753/HOME?CRETRY=1&SRETRY=0> |
| Date of search | 16/07/2009 |
| Years covered | 2005-July 2009. Frolich et al (2007) searched till June 2005. |
| Search Strategy | | #1 | [MeSH descriptor **Salaries and Fringe Benefits** explode all trees](http://www3.interscience.wiley.com/cochrane/searchHistory?mode=runquery&qnum=1) | 561 | | --- | --- | --- | | #2 | [MeSH descriptor **Reimbursement, Incentive** explode all trees](http://www3.interscience.wiley.com/cochrane/searchHistory?mode=runquery&qnum=2) | 48 | | #3 | [MeSH descriptor **Fees and Charges** explode all trees](http://www3.interscience.wiley.com/cochrane/searchHistory?mode=runquery&qnum=3) | 1079 | | #4 | [p4q](http://www3.interscience.wiley.com/cochrane/searchHistory?mode=runquery&qnum=4) | 0 | | #5 | [p4p](http://www3.interscience.wiley.com/cochrane/searchHistory?mode=runquery&qnum=5) | 2 | | #6 | [pay*](http://www3.interscience.wiley.com/cochrane/searchHistory?mode=runquery&qnum=6) | 5006 | | #7 | [incentive*](http://www3.interscience.wiley.com/cochrane/searchHistory?mode=runquery&qnum=7) | 1146 | | #8 | [bonus*](http://www3.interscience.wiley.com/cochrane/searchHistory?mode=runquery&qnum=8) | 50 | | #9 | [MeSH descriptor **Treatment Outcome** explode all trees](http://www3.interscience.wiley.com/cochrane/searchHistory?mode=runquery&qnum=9) | 58725 | | #10 | [MeSH descriptor **Medical Errors** explode all trees](http://www3.interscience.wiley.com/cochrane/searchHistory?mode=runquery&qnum=10) | 1720 | | #11 | [MeSH descriptor **Quality Control** explode all trees](http://www3.interscience.wiley.com/cochrane/searchHistory?mode=runquery&qnum=11) | 382 | | #12 | [MeSH descriptor **Cost-Benefit Analysis** explode all trees](http://www3.interscience.wiley.com/cochrane/searchHistory?mode=runquery&qnum=12) | 14751 | | #13 | [MeSH descriptor **Safety** explode all trees](http://www3.interscience.wiley.com/cochrane/searchHistory?mode=runquery&qnum=13) | 0 | | #14 | [MeSH descriptor **Health Services Accessibility** explode all trees](http://www3.interscience.wiley.com/cochrane/searchHistory?mode=runquery&qnum=14) | 979 | | #15 | [quality](http://www3.interscience.wiley.com/cochrane/searchHistory?mode=runquery&qnum=15) | 50940 | | #16 | [outcome*](http://www3.interscience.wiley.com/cochrane/searchHistory?mode=runquery&qnum=16) | 124378 | | #17 | [performance](http://www3.interscience.wiley.com/cochrane/searchHistory?mode=runquery&qnum=17) | 29573 | | #18 | [error*](http://www3.interscience.wiley.com/cochrane/searchHistory?mode=runquery&qnum=18) | 10574 | | #19 | [safety*](http://www3.interscience.wiley.com/cochrane/searchHistory?mode=runquery&qnum=19) | 42639 | | #20 | [access*](http://www3.interscience.wiley.com/cochrane/searchHistory?mode=runquery&qnum=20) | 389266 | | #21 | [equity](http://www3.interscience.wiley.com/cochrane/searchHistory?mode=runquery&qnum=21) | 195 | | #22 | [cost effectiveness](http://www3.interscience.wiley.com/cochrane/searchHistory?mode=runquery&qnum=22) | 18992 | | #23 | [effectiveness](http://www3.interscience.wiley.com/cochrane/searchHistory?mode=runquery&qnum=23) | 47031 | | #24 | [(**#9** OR **#10** OR **#11** OR **#12** OR **#13** OR **#14** OR **#15** OR **#16** OR **#17** OR **#18** OR **#19** OR **#20** OR **#21** OR **#22** OR **#23**)](http://www3.interscience.wiley.com/cochrane/searchHistory?mode=runquery&qnum=24) | 453881 | | #25 | [MeSH descriptor **Hospitals** explode all trees](http://www3.interscience.wiley.com/cochrane/searchHistory?mode=runquery&qnum=25) | 2913 | | #26 | [MeSH descriptor **Physicians** explode all trees](http://www3.interscience.wiley.com/cochrane/searchHistory?mode=runquery&qnum=26) | 847 | | #27 | [hospital*](http://www3.interscience.wiley.com/cochrane/searchHistory?mode=runquery&qnum=27) | 122116 | | #28 | [physician*](http://www3.interscience.wiley.com/cochrane/searchHistory?mode=runquery&qnum=28) | 17387 | | #29 | [practitioner*](http://www3.interscience.wiley.com/cochrane/searchHistory?mode=runquery&qnum=29) | 6203 | | #30 | [(**#25** OR **#26** OR **#27** OR **#28** OR **#29**)](http://www3.interscience.wiley.com/cochrane/searchHistory?mode=runquery&qnum=30) | 135710 | | #31 | [(**#1** OR **#2** OR **#3** OR **#4** OR **#5** OR **#6** OR **#7** OR **#8**)](http://www3.interscience.wiley.com/cochrane/searchHistory?mode=runquery&qnum=31) | 7480 | | #32 | [(#24 AND #30 AND #31), from 2005 to 2009](http://www3.interscience.wiley.com/cochrane/searchHistory?mode=runquery&qnum=32) | 1175 |   Further selection of clinical trials and technology assessments |
| Language restrictions | none |
| Number of citations | 251 |

**eTable 4: Primary evaluation studies electronic search strategy per database (Cont.)**

| Database | **PsycINFO** |
| --- | --- |
| Host | <http://ovidsp.tx.ovid.com/spa/ovidweb.cgi?T=JS&D=psyh&PAGE=main> |
| Date of search | 16/07/2009 |
| Years covered | 2004-July 2009. This database was most recently covered by Rosenthal & Frank (2006), who searched till 2003. |
| Search Strategy | 1. exp salaries/ or exp bonuses/ or exp employee benefits/ or exp professional fees/  2. incentives/ or exp monetary incentives/ or exp "awards (merit)"/ or exp rewards/  3. p4p.mp. [mp=title, abstract, heading word, table of contents, key concepts]  4. p4q.mp. [mp=title, abstract, heading word, table of contents, key concepts]  5. pay*.mp. [mp=title, abstract, heading word, table of contents, key concepts]  6. incentive*.mp. [mp=title, abstract, heading word, table of contents, key concepts]  7. bonus*.mp. [mp=title, abstract, heading word, table of contents, key concepts]  8. exp Treatment Effectiveness Evaluation/  9. exp "Quality of Care"/  10. exp Errors/  11. exp Treatment Outcomes/  12. exp Quality Control/  13. exp "Costs and Cost Analysis"/  14. exp Safety/  15. exp health disparities/  16. quality.mp. [mp=title, abstract, heading word, table of contents, key concepts]  17. outcome*.mp. [mp=title, abstract, heading word, table of contents, key concepts]  18. error*.mp. [mp=title, abstract, heading word, table of contents, key concepts]  19. safety*.mp. [mp=title, abstract, heading word, table of contents, key concepts]  20. access*.mp. [mp=title, abstract, heading word, table of contents, key concepts]  21. equity.mp. [mp=title, abstract, heading word, table of contents, key concepts]  22. effectiveness.mp. [mp=title, abstract, heading word, table of contents, key concepts]  23. exp Hospitals/  24. exp Physicians/  25. exp Health Care Services/  26. hospital*.mp. [mp=title, abstract, heading word, table of contents, key concepts]  27. physician*.mp. [mp=title, abstract, heading word, table of contents, key concepts]  28. practitioner*.mp. [mp=title, abstract, heading word, table of contents, key concepts]  29. healthcare.mp. [mp=title, abstract, heading word, table of contents, key concepts]  30. 22 or 8 or 18 or 14 or 9 or 17 or 12 or 11 or 19 or 15 or 21 or 20 or 10 or 13 or 16  31. 24 or 29 or 25 or 27 or 23 or 28 or 26  32. 6 or 4 or 1 or 3 or 7 or 2 or 5  33. 32 and 30 and 31  34. limit 33 to (human and abstracts and "0110 peer-reviewed journal" and journal article and yr="2004 - 2009") |
| Language restrictions | none |
| Number of citations | 699 |

**eTable 4: Primary evaluation studies electronic search strategy per database (Cont.)**

| Database | **EconLit** |
| --- | --- |
| Host | <http://csaweb110v.csa.com/> |
| Date of search | 16/07/2009 |
| Years covered | 2004-July 2009. This database was most recently covered by Rosenthal & Frank (2006), who searched till 2003. |
| Search Strategy | ((KW=(p4q or p4p or pay*) or KW=(incentive* or compensation* or reimbursement*) or KW=(bonus* or remunerat*)) or(DE=("pay" or "salary")) or(DE=("incentive compatibility" or "bonuses"))) and((DE=("cost effectiveness" or "quality" or "safety")) or(KW=(quality or outcome* or error*) or KW=(effectiveness or safety or access*) or KW=(disparit* or equit*))) and((DE=("healthcare" or "hospital" or "physician")) or(KW=(hospital* or physician* or practitioner*) or KW=healthcare)) |
| Language restrictions | none |
| Number of citations | 248 |

**eTable 5: Primary evaluation studies relevance screening. Rationale for exclusion based on full text review**

| Citation | Patient/ population | Intervention | Outcome | Design |
| --- | --- | --- | --- | --- |
| Hemenway et al, 1995 ^(81)^ |  | No P4P evaluation |  |  |
| Shen, 2003 ^(82)^ | Mental health |  |  |  |
| Dixon et al, 2004 ^(83)^ |  | Managed care, no P4P |  |  |
| Eggleston & Hsieh, 2004 ^(84)^ |  | Cost sharing |  |  |
| Feldman et al, 2004 ^(85)^ | Mental health |  |  | Panel discussion |
| Forsberg et al, 2004 ^(86)^ |  |  | Only efficiency focused |  |
| Laurence et al, 2004 ^(87)^ |  | No P4P |  |  |
| Mullen, 2004 ^(88)^ |  | Indicator use as such |  |  |
| St Jacques et al, 2004 ^(89)^ |  |  | Only productivity focused |  |
| Zivin & Pfaff, 2004 ^(90)^ |  | No P4P |  |  |
| Beersen et al, 2005 ^(91)^ |  | No P4P |  |  |
| Feldman et al, 2005 ^(92)^ | Mental health |  |  |  |
| Gandjour & Lauterbach, 2005 ^(93)^ |  | No P4P |  |  |
| Grembowski et al, 2005 ^(94)^ |  | Managed care |  |  |
| Jack, 2005 ^(95)^ |  | Cost sharing |  |  |
| Koffman et al, 2005 ^(96)^ |  | Patient incentives |  |  |
| Mentari et al, 2005 ^(97)^ |  | Reimbursement elevation as such |  |  |
| Saitto et al, 2005 ^(98)^ |  | Prospective payment |  |  |
| Schneider et al, 2005 ^(99)^ | Mental health |  |  | No evaluation |
| Shepard et al, 2005 ^(100)^ | Mental health |  |  |  |
| Spertus et al, 2005 ^(101)^ |  | No P4P |  |  |
| Bachman, 2006 ^(102)^ | Behavioral health |  |  | No evaluation |
| Backstrom & Mjorndal, 2006 ^(103)^ |  | No direct financial incentive (lottery) |  |  |
| Bloche, 2006 ^(104)^ |  | Tax exemption, no P4P |  |  |
| Ginsburg, 2006 ^(105)^ |  | No P4P |  |  |
| Paleologou et al, 2006 ^(106)^ |  | Development of HRM attitude measurement instrument, including wages |  |  |
| Thomas et al, 2006 ^(107)^ | Mental health |  |  |  |
| Biai et al, 2007 ^(108)^ |  | Cost reimbursement of additional administration |  |  |
| Cotter, 2007 ^(109)^ |  | No P4P |  |  |
| Harrison et al, 2007 ^(110)^ |  | Cost quality combination, no P4P |  |  |
| Huddle, 2007 ^(111)^ |  | No P4P |  |  |
| Ittner et al, 2007 ^(112)^ |  |  | Only productivity focused |  |
| Martens et al, 2007 ^(113)^ |  | Incentive linked to an intent, not to actual performance |  |  |
| Pelonero et al, 2007 ^(114)^ | Mental health |  |  |  |
| Pronovost et al, 2007 ^(115)^ |  | No P4P |  |  |
| Bottle et al, 2008 ^(116)^ |  | No P4P |  |  |
| Boucai & Zonszein, 2008 ^(117)^ |  | No P4P |  |  |
| Buetow & Bryant, 2008 ^(118)^ |  |  |  | Comment, no primary evaluation |
| Chung et al, 2008 ^(119)^ |  | No P4P |  |  |
| Greene et al, 2008 ^(120)^ |  | Not EBP based | Cost reduction, variability reduction |  |
| Quimbo et al, 2008 ^(121)^ |  | Payment vs no payment, no target or intervention handled |  |  |
| Scholle et al, 2008 ^(122)^ |  |  | Reliability of measures |  |
| Sinsky et al, 2008 ^(123)^ |  | How to present measures in guidelines |  |  |
| Tuerk et al, 2008 ^(124)^ |  | No P4P |  |  |
| Wright et al, 2008 ^(125)^ |  | No P4P |  |  |
| Arling et al, 2009 ^(126)^ | Nursing homes |  |  | No evaluation |
| Chung & Shauver, 2009 ^(127)^ |  |  |  | Comment, no primary evaluation |
| Eggleston & Bir, 2009 ^(128)^ |  | No P4P |  |  |
| Gellad et al, 2009 ^(129)^ |  |  |  | Comment, no primary evaluation |
| Ham et al, 2009 ^(130)^ |  |  |  | No evaluation |
| Hartzband & Groopman, 2009 ^(131)^ |  | No P4P |  | Comment, no primary evaluation |
| Hirth et al, 2009 ^(132)^ |  | No P4P, no incentive |  |  |
| Kaplan et al, 2009 ^(133)^ |  | How to construct composite measures |  |  |
| Lee & Ferris, 2009 ^(134)^ |  |  |  | Editorial, no evaluation |
| Lehman & Krumholz, 2009 ^(135)^ |  |  |  | Editorial, no evaluation |
| Lippi Bruni et al, 2009 ^(136)^ |  | Pay for participation, pay for compliance without performance verification |  |  |
| Marco & Bottles, 2009 ^(137)^ |  |  |  | Comment, no primary evaluation |
| Petersen et al, 2009 ^(138)^ |  | No P4P |  |  |
| Rother, 2009 ^(139)^ |  |  |  | Comment, no primary evaluation |
| Scholle et al, 2009 ^(140)^ |  |  | The number of events as an input into P4P |  |
| Zimmerman et al, 2009 ^(141)^ |  | Incentives directed at employees in a patient role, not in a staffing role. |  |  |

Footnote: In addition to these papers, a total of 151 publications were also excluded after full text review, because they were conceptual in nature and reported no empirical findings.

**eTable 6: Primary evaluation studies quality appraisal**

|  | Research question | Patient popu-lation and setting | | | Interven-tion | | Compa-rison | | Out-come | | De-sign | Sample size | | Statis-tics | | Generali-sability | Confoun-ders addressed | | | Randomi-zation | Blin-ding | Cluste-ring effect | Nr. data points | Total score |
| --- | --- | --- | --- | --- | --- | --- | --- | --- | --- | --- | --- | --- | --- | --- | --- | --- | --- | --- | --- | --- | --- | --- | --- | --- |
| Feely et al, 1990 ^(142)^ | 1 | 1 | | | 1 | | 0 | | 1 | | 1 | 1 | | -1 | | -1 | -1 | | | 0 | 0 | 0 | 0 | 3 |
| Reid et al, 1991 ^(143)^ | 1 | 1 | | | 1 | | 0 | | 1 | | 1 | 1 | | -1 | | 1 | -1 | | | 0 | 0 | 0 | 0 | 5 |
| Ritchie et al, 1992 ^(144)^ | 1 | 1 | | | 1 | | 0 | | 1 | | 1 | 1 | | 1 | | 1 | 1 | | | 0 | 0 | 0 | 0 | 9 |
| Kouides et al, 1993 ^(145)^; Bennett et al, 1994 ^(146)^ | 1 | 1 | | | 1 | | 1 | | 1 | | 1 | -1 | | 1 | | 1 | 1 | | | 0 | 0 | 0 | 0 | 8 |
| Lynch, 1995 ^(147)^ | 1 | 1 | | | 1 | | 0 | | 1 | | 1 | 1 | | 1 | | 1 | 1 | | | 0 | 0 | 0 | 0 | 9 |
| Morrow et al, 1995 ^(148)^ | 1 | 1 | | | 1 | | 0 | | 1 | | 1 | 1 | | 1 | | 1 | 1 | | | 0 | 0 | 0 | 0 | 9 |
| Fairbrother et al, 1997 ^(149)^ | 1 | 1 | | | 1 | | 0 | | 1 | | 1 | -1 | | 1 | | 1 | 1 | | | 0 | 0 | 1 | 0 | 8 |
| Grady et al, 1997 ^(150)^ | 1 | 1 | | | 1 | | 1 | | 1 | | 1 | 1 | | 1 | | -1 | 1 | | | 1 | 0 | 0 | 0 | 9 |
| Hillman et al, 1998 ^(151)^ | 1 | 1 | | | 1 | | 1 | | 1 | | 1 | 1 | | 1 | | 1 | 1 | | | 1 | 0 | 0 | 0 | 11 |
| Kouides et al, 1998 ^(152)^ | 1 | 1 | | | 1 | | 1 | | 1 | | 1 | -1 | | 1 | | 1 | 1 | | | 1 | 0 | 0 | 0 | 9 |
| Cameron et al, 1999 ^(153)^ | 1 | 1 | | | 1 | | 0 | | 1 | | 1 | 1 | | -1 | | 1 | 1 | | | 0 | 0 | 0 | 1 | 8 |
| Fairbrother et al, 1999 ^(154)^ | 1 | 1 | | | 1 | | 1 | | 1 | | 1 | -1 | | 1 | | 1 | 1 | | | 1 | 1 | 1 | 0 | 11 |
| Hillman et al, 1999 ^(155)^ | 1 | 1 | | | 1 | | 1 | | 1 | | 1 | 1 | | 1 | | 1 | 1 | | | 1 | 1 | 0 | 0 | 12 |
| Hopkins, 1999 ^(156)^ | 1 | 1 | | | 1 | | 0 | | -1 | | 1 | 1 | | -1 | | -1 | 1 | | | 0 | 0 | 0 | 0 | 3 |
| Lebaron et al, 1999 ^(157)^ | 1 | 1 | | | 1 | | 1 | | 1 | | 1 | 1 | | -1 | | 1 | 1 | | | 0 | 0 | 0 | 0 | 8 |
| Schauffler et al, 1999 ^(158)^ | Purely descriptive study of % targets attained, no statistical analysis present. | | | | | | | | | | | | | | | | | | | 0 | 0 | 0 | 0 | Over-rule |
| Woodson, 1999 ^(159)^ | Two descriptive case studies, only narrative results reporting. | | | | | | | | | | | | | | | | | | | 0 | 0 | 0 | 0 | Over-rule |
| Safran et al, 2000 ^(160)^ | 1 | | 1 | 1 | | 0 | | 1 | | 1 | | 1 | 1 | | 1 | | | 1 | | 0 | 0 | 0 | 0 | 9 |
| Sussman et al, 2002 ^(161)^ | No reported P4Q description and outcomes, which is one of the intervention components | | | | | | | | | | | | | | | | | | 0 | | 0 | 0 | 0 | Over-rule |
| Cattaneo et al, 2001 ^(162)^ | 1 | | 1 | 1 | | 0 | | 1 | | 1 | | 1 | 1 | | 1 | | | 1 | | 0 | 0 | 0 | 0 | 9 |
| Coleman et al, 2001 ^(163)^ | 1 | | 1 | 1 | | 1 | | 1 | | 1 | | 1 | -1 | | 1 | | | -1 | | 1 | 0 | -1 | 0 | 6 |
| Fairbrother et al, 2001 ^(164)^ | 1 | | 1 | 1 | | 1 | | 1 | | 1 | | 1 | 1 | | 1 | | | 1 | | 1 | 0 | 1 | 0 | 12 |
| Shortell et al, 2001 ^(165)^ | 1 | | 1 | 1 | | 0 | | 1 | | 1 | | 1 | 1 | | 1 | | | 1 | | 0 | 0 | 0 | 0 | 9 |
| Ashworth et al, 2002b ^(166)^ | primary outcome results (effect on prescribing) not shown, no figures, only narrative | | | | | | | | | | | | | | | | | | 0 | | 0 | 0 | 0 | Over-rule |
| Ashworth et al, 2002a ^(167)^ | descriptive survey design, without any result related relationship testing | | | | | | | | | | | | | | | | | | 0 | | 0 | 0 | 0 | Over-rule |
| Bond et al, 2002 ^(168)^ | 1 | | 1 | 1 | | 0 | | 1 | | 1 | | 1 | 1 | | 1 | | | 1 | | 0 | 0 | 0 | 0 | 9 |
| Amundson et al, 2003 ^(169)^ | 1 | | 1 | 1 | | 0 | | 1 | | 1 | | 1 | 1 | | 1 | | | 1 | | 0 | 0 | 0 | 0 | 9 |
| Casalino et al, 2003 ^(170)^ | 1 | | 1 | 1 | | 0 | | 1 | | 1 | | 1 | 1 | | 1 | | | 1 | | 0 | 0 | 0 | 0 | 9 |
| Chung et al, 2003 ^(171)^ | 1 | | 1 | 1 | | 0 | | 1 | | 1 | | 1 | 1 | | 1 | | | 1 | | 0 | 0 | 0 | 0 | 9 |
| Larsen et al, 2003 ^(172)^ | 1 | | 1 | 1 | | 0 | | 1 | | 1 | | 1 | 1 | | 1 | | | 1 | | 0 | 0 | 0 | 0 | 9 |
| McMenamin et al, 2003 ^(173)^ | 1 | | 1 | 1 | | 0 | | 1 | | 1 | | 1 | 1 | | 1 | | | 1 | | 0 | 0 | 0 | 0 | 9 |
| Roski et al, 2003 ^(174)^ | 1 | | 1 | 1 | | 1 | | 1 | | 1 | | 1 | 1 | | 1 | | | 1 | | 1 | 0 | 1 | 0 | 12 |
| Amour et al, 2004 ^(175)^ | 1 | | 1 | 1 | | 0 | | 1 | | 1 | | 1 | 1 | | 1 | | | 1 | | 0 | 0 | 0 | 0 | 9 |
| Ashworth et al, 2004 ^(176)^ | Although presented as quality – incentive effects the author acknowledges the lack of reporting this primary outcome. | | | | | | | | | | | | | | | | | | 0 | | 0 | 0 | 0 | Over-rule |
| Berthiaume et al, 2004 ^(177)^ | Purely descriptive, no results testing, no outcome reporting. | | | | | | | | | | | | | | | | | | 0 | | 0 | 0 | 0 | Over-rule |
| Borenstein et al, 2004 ^(8)^ | Selection of accreditation applicants as participants. Too big threat to generalisability, | | | | | | | | | | | | | | | | | | 0 | | 0 | 0 | 0 | Over-rule |
| Greene et al, 2004 ^(178)^ | 1 | | 1 | 1 | | 0 | | 1 | | 1 | | 1 | 1 | | 1 | | | 1 | | 0 | 0 | 0 | 0 | 9 |
| Hippisley-cox et al, 2004 ^(179)^ | 1 | | 1 | 1 | | 0 | | 1 | | 1 | | 1 | 1 | | 1 | | | 1 | | 0 | 0 | 0 | 0 | 9 |
| Keating et al, 2004 ^(180)^ | Although very limited P4Q results in univariate analysis, these are not further addressed (confounders, generalisability,,,,) | | | | | | | | | | | | | | | | | | 0 | | 0 | 0 | 0 | Over-rule |
| Li et al, 2004 ^(181)^ | 1 | | 1 | 1 | | 0 | | 1 | | 1 | | 1 | 1 | | 1 | | | 1 | | 0 | 0 | 0 | 0 | 9 |
| McMenamin et al, 2004 ^(182)^ | 1 | | 1 | 1 | | 0 | | 1 | | 1 | | 1 | 1 | | 1 | | | 1 | | 0 | 0 | 0 | 0 | 9 |
| Schmittdiel et al, 2004 ^(183)^ | 1 | | 1 | 1 | | 0 | | 1 | | 1 | | 1 | 1 | | 1 | | | 1 | | 0 | 0 | 0 | 0 | 9 |
| Wickizer et al, 2004 ^(184)^ | No statistical testing for time trends in fig 1 and 2, the only P4Q congruent process measures reported. | | | | | | | | | | | | | | | | | | 0 | | 0 | 0 | 0 | Over-rule |
| Ashworth et al, 2005 ^(185)^ | 1 | | 1 | 1 | | 0 | | 1 | | 1 | | 1 | 1 | | 1 | | | 1 | | 0 | 0 | 0 | 0 | 9 |
| Beaulieu & Horrigan, 2005 ^(186)^ | 1 | | 1 | 1 | | 1 | | 1 | | 1 | | 1 | 1 | | -1 | | | 1 | | 0 | 0 | 0 | 0 | 8 |
| Harries et al, 2005 ^(187)^ | no statistical testing. | | | | | | | | | | | | | | | | | | 0 | | 0 | 0 | 0 | Over-rule |
| Majeed et al, 2005 ^(188)^ | no statistical testing. | | | | | | | | | | | | | | | | | | 0 | | 0 | 0 | 0 | Over-rule |
| May, 2005 ^(189)^ | descriptive, no testing. | | | | | | | | | | | | | | | | | | 0 | | 0 | 0 | 0 | Over-rule |
| Pourat et al, 2005 ^(190)^ | 1 | | 1 | 1 | | 0 | | 1 | | 1 | | 1 | 1 | | 1 | | | 1 | | 0 | 0 | 0 | 0 | 9 |
| Qual letter, 2005 ^(191)^ | narrative, descriptive, no statistical analysis | | | | | | | | | | | | | | | | | | 0 | | 0 | 0 | 0 | Over-rule |
| Qual letter, 2005b ^(192)^ | narrative, descriptive, no statistical analysis | | | | | | | | | | | | | | | | | | 0 | | 0 | 0 | 0 | Over-rule |
| Rosenthal et al, 2005 ^(193)^ | 1 | | 1 | 1 | | 1 | | 1 | | 1 | | 1 | 1 | | 1 | | | 1 | | 0 | 0 | 0 | 0 | 10 |
| Shenkman et al, 2005 ^(194)^ | 1 | | 1 | 1 | | 0 | | 1 | | 1 | | 1 | 1 | | 1 | | | 1 | | 0 | 0 | 0 | 0 | 9 |
| Sperl-hillen & O'Connor, 2005 ^(195)^ | P4Q only used in last year of 10 year study, was not statistically tested. | | | | | | | | | | | | | | | | | | 0 | | 0 | 0 | 0 | Over-rule |
| Bailit, 2006 ^(196)^ | descriptive case studies, no further evaluation | | | | | | | | | | | | | | | | | | 0 | | 0 | 0 | 0 | Over-rule |
| Doran et al, 2006 ^(197)^ | 1 | | 1 | 1 | | 0 | | 1 | | 1 | | 1 | 1 | | 1 | | | 1 | | 0 | 0 | 0 | 0 | 9 |
| Ettner et al, 2006 ^(198)^ | 1 | | 1 | 1 | | 0 | | 1 | | 1 | | 1 | 1 | | 1 | | | 1 | | 0 | 1 | 1 | 0 | 11 |
| Francis et al, 2006 ^(199)^ | 1 | | 1 | -1 | | 0 | | 1 | | 1 | | 1 | 1 | | 1 | | | 1 | | 0 | 0 | 0 | 0 | 7 |
| Grossbart, 2006 ^(200)^ | 1 | | 1 | 1 | | 1 | | 1 | | 1 | | 1 | 1 | | -1 | | | 1 | | 0 | 0 | 0 | 0 | 8 |
| Healy et al, 2006 ^(201)^ | descriptive Australia profile, no scientific evaluation | | | | | | | | | | | | | | | | | | 0 | | 0 | 0 | 0 | Over-rule |
| Jaiveer et al, 2006 ^(202)^ | 1 | | 1 | 1 | | 0 | | 1 | | 1 | |  | 1 | | 1 | | | 1 | | 0 | 0 | 0 | 0 | 8 |
| Levin-Scherz et al, 2006 ^(203)^ | 1 | | 1 | 1 | | 0 | | 1 | | 1 | | 1 | 1 | | 1 | | | 1 | | 0 | 0 | 0 | 0 | 9 |
| McLean et al, 2006 ^(204)^ | 1 | | 1 | 1 | | 0 | | 1 | | 1 | | 1 | 1 | | 1 | | | 1 | | 0 | 0 | 0 | 0 | 9 |
| Pines, 2006 ^(205)^ | case studies and comment only, no evaluation | | | | | | | | | | | | | | | | | | 0 | | 0 | 0 | 0 | Over-rule |
| Ramsay et al, 2006 ^(206)^ | no statistical analysis | | | | | | | | | | | | | | | | | | 0 | | 0 | 0 | 0 | Over-rule |
| Reiter et al, 2006 ^(207)^ | 1 | | 1 | 1 | | 0 | | 1 | | 1 | | 1 | 1 | | 1 | | | 1 | | 0 | 0 | 0 | 0 | 9 |
| Reschovsky et al, 2006 ^(208)^ | 1 | | 1 | 1 | | 0 | | 1 | | 1 | | 1 | 1 | | 1 | | | 1 | | 0 | 0 | 0 | 0 | 9 |
| Rittenhouse & Robinson, 2006 ^(209)^ | 1 | | 1 | 1 | | 0 | | 1 | | 1 | | 1 | 1 | | 1 | | | 1 | | 0 | 0 | 0 | 0 | 9 |
| Sigfrid et al, 2006 ^(210)^ | 1 | | 1 | 1 | | 0 | | 1 | | 1 | | 1 | 1 | | 1 | | | 1 | | 0 | 0 | 0 | 0 | 9 |
| Simpson et al, 2006 ^(211)^ | 1 | | 1 | 1 | | 0 | | 1 | | 1 | | 1 | 1 | | 1 | | | 1 | | 0 | 0 | 0 | 0 | 9 |
| Srilangalingam et al, 2006 ^(212)^ | 1 | | 1 | 1 | | 0 | | 1 | | 1 | | 1 | 1 | | 1 | | | 1 | | 0 | 0 | 0 | 0 | 9 |
| Strong et al, 2006 ^(213)^ | 1 | | 1 | 1 | | 0 | | 1 | | 1 | | 1 | 1 | | 1 | | | 1 | | 0 | 0 | 0 | 0 | 9 |
| Sutton & Mclean, 2006 ^(214)^ | 1 | | 1 | 1 | | 0 | | 1 | | 1 | | 1 | 1 | | 1 | | | 1 | | 0 | 0 | 0 | 0 | 9 |
| Trisolini et al, 2006 ^(215)^ | No statistical analysis | | | | | | | | | | | | | | | | | | 0 | | 0 | 0 | 0 | Over-rule |
| Wang et al, 2006 ^(216)^ | 1 | | 1 | 1 | | 0 | | 1 | | 1 | | 1 | 1 | | 1 | | | 1 | | 0 | 0 | 0 | 0 | 9 |
| Whalley et al, 2006 ^(217)^ | testing only right before P4P implementation | | | | | | | | | | | | | | | | | | 0 | | 0 | 0 | 0 | Over-rule |
| Williams et al, 2006a ^(218)^ | 1 | | 1 | 1 | | 1 | | 1 | | 1 | | 1 | -1 | | 1 | | | 1 | | 0 | 0 | 0 | 0 | 8 |
| Williams et al, 2006b ^(219)^ | 1 | | 1 | 1 | | 1 | | 1 | | 1 | | 1 | 1 | | 1 | | | 1 | | 0 | 0 | 0 | 0 | 10 |
| Wright et al, 2006 ^(220)^ | 1 | | 1 | 1 | | 0 | | 1 | | 1 | | 1 | 1 | | 1 | | | 1 | | 0 | 0 | 0 | 0 | 9 |
| Ashworth et al, 2007 ^(221)^ | 1 | | 1 | 1 | | 0 | | 1 | | 1 | | 1 | 1 | | 1 | | | 1 | | 0 | 0 | 0 | 0 | 9 |
| Ashworth et al, 2007b ^(222)^ | 1 | | 1 | 1 | | 0 | | 1 | | 1 | | 1 | 1 | | 1 | | | 1 | | 0 | 0 | 0 | 0 | 9 |
| Campbell et al, 2007 ^(223)^ | 1 | | 1 | 1 | | 0 | | 1 | | 1 | | 1 | 1 | | 1 | | | 1 | | 0 | 0 | 0 | -1 | 8 |
| Carey et al, 2007 ^(224)^ | no relationship or effect testing | | | | | | | | | | | | | | | | | | 0 | | 0 | 0 | 0 | Over-rule |
| Casale et al, 2007 ^(225)^ | 1 | | 1 | 1 | | 0 | | 1 | | 1 | | 1 | 1 | | 1 | | | 1 | | 0 | 0 | 0 | 0 | 9 |
| Coleman et al, 2007a ^(226)^ | 1 | | 1 | 1 | | 0 | | 1 | | 1 | | 1 | 1 | | 1 | | | 1 | | 0 | 0 | 0 | 0 | 9 |
| Coleman et al, 2007b ^(227)^ | 1 | | 1 | 1 | | 0 | | 1 | | 1 | | 1 | 1 | | 1 | | | 1 | | 0 | 0 | 0 | 0 | 9 |
| Downing et al, 2007 ^(228)^ | 1 | | 1 | 1 | | 0 | | 1 | | 1 | | 1 | 1 | | 1 | | | 1 | | 0 | 0 | 0 | 0 | 9 |
| Felt-Lisk et al, 2007 ^(229)^ | no significance testing | | | | | | | | | | | | | | | | | | 0 | | 0 | 0 | 0 | Over-rule |
| Gene badia et al, 2007 ^(230)^ | 1 | | 1 | 1 | | 0 | | 1 | | 1 | | 1 | 1 | | 1 | | | 1 | | 0 | 0 | 0 | 0 | 9 |
| Gilmore et al, 2007 ^(231)^ | 1 | | 1 | 1 | | 1 | | 1 | | 1 | | 1 | 1 | | 1 | | | 1 | | 0 | 0 | 0 | 0 | 10 |
| Glickman et al, 2007 ^(232)^ | 1 | | 1 | 1 | | 1 | | 1 | | 1 | | 1 | 1 | | 1 | | | 1 | | 0 | 0 | 1 | 0 | 11 |
| Gray et al, 2007 ^(233)^ | 1 | | 1 | 1 | | 0 | | 1 | | 1 | | 1 | 1 | | 1 | | | 1 | | 0 | 0 | 0 | 1 | 10 |
| Gulliford et al, 2007 ^(234)^ | 1 | | 1 | 1 | | 0 | | 1 | | 1 | | 1 | 1 | | 1 | | | 1 | | 0 | 0 | 0 | 0 | 9 |
| Halanych et al, 2007 ^(235)^ | no testing or adjustment for P4Q | | | | | | | | | | | | | | | | | | 0 | | 0 | 0 | 0 | Over-rule |
| Helm & Tortorella, 2007 ^(236)^ | some response rates below 50% | | | | | | | | | | | | | | | | | | 0 | | 0 | 0 | 0 | Over-rule |
| Heneghan et al, 2007 ^(237)^ | only 50% response rate | | | | | | | | | | | | | | | | | | 0 | | 0 | 0 | 0 | Over-rule |
| Hughes, 2007 ^(238)^ | no referencing, no testing | | | | | | | | | | | | | | | | | | 0 | | 0 | 0 | 0 | Over-rule |
| Kautter et al, 2007 ^(239)^ | descriptive case study findings, no further evaluation or testing | | | | | | | | | | | | | | | | | | 0 | | 0 | 0 | 0 | Over-rule |
| Lindenauer et al, 2007 ^(240)^ | 1 | | 1 | 1 | | 1 | | 1 | | 1 | | 1 | 1 | | 1 | | | 1 | | 0 | 0 | 0 | 0 | 10 |
| Mandel & Kotagal, 2007 ^(241)^ | no statistical testing | | | | | | | | | | | | | | | | | | 0 | | 0 | 0 | 0 | Over-rule |
| McCarlie et al, 2007 ^(242)^ | no statistical testing | | | | | | | | | | | | | | | | | | 0 | | 0 | 0 | 0 | Over-rule |
| McLean et al, 2007a ^(243)^ | 1 | | 1 | 1 | | 0 | | 1 | | 1 | | 1 | 1 | | 1 | | | 1 | | 0 | 0 | 0 | 0 | 9 |
| McLean et al, 2007b ^(244)^ | 1 | | 1 | 1 | | 0 | | 1 | | 1 | | 1 | 1 | | 1 | | | 1 | | 0 | 0 | 0 | 0 | 9 |
| Mehrotra et al, 2007 ^(245)^ | 1 | | 1 | 1 | | 0 | | 1 | | 1 | | 1 | 1 | | 1 | | | 1 | | 0 | 0 | 0 | 0 | 9 |
| Millett et al, 2007a ^(246)^ | 1 | | 1 | 1 | | 0 | | 1 | | 1 | | 1 | 1 | | 1 | | | 1 | | 0 | 0 | 1 | 0 | 10 |
| Millett et al, 2007b ^(247)^ | 1 | | 1 | 1 | | 0 | | 1 | | 1 | | 1 | 1 | | 1 | | | 1 | | 0 | 0 | 1 | 0 | 10 |
| Millett et al, 2007c ^(248)^ | 1 | | 1 | 1 | | 0 | | 1 | | 1 | | 1 | 1 | | 1 | | | 1 | | 0 | 0 | 0 | 0 | 9 |
| Nalli et al, 2007 ^(249)^ | no statistical testing, significance analysis | | | | | | | | | | | | | | | | | | 0 | | 0 | 0 | 0 | Over-rule |
| O’Malley et al, 2007 ^(250)^ | 1 | | 1 | 1 | | 0 | | 1 | | 1 | | 1 | 1 | | 1 | | | 1 | | 0 | 0 | 0 | 0 | 9 |
| Patel et al, 2007 ^(251)^ | no statistical analysis | | | | | | | | | | | | | | | | | | 0 | | 0 | 0 | 0 | Over-rule |
| Rosenthal & Camillus, 2007 ^(252)^ | descriptive, no testing, no references | | | | | | | | | | | | | | | | | | 0 | | 0 | 0 | 0 | Over-rule |
| Saxena et al, 2007 ^(253)^ | 1 | | 1 | 1 | | 0 | | 1 | | 1 | | 1 | 1 | | 1 | | | 1 | | 0 | 0 | 0 | 0 | 9 |
| Shohet et al, 2007 ^(254)^ | 1 | | 1 | 1 | | 0 | | 1 | | 1 | | 1 | 1 | | 1 | | | 1 | | 0 | 0 | 0 | 0 | 9 |
| Simon et al, 2007 ^(255)^ | 1 | | 1 | 1 | | 0 | | 1 | | 1 | | 1 | 1 | | 1 | | | 1 | | 0 | 0 | 0 | 0 | 9 |
| Simpson et al, 2007 ^(256)^ | 1 | | 1 | 1 | | 0 | | 1 | | 1 | | 1 | 1 | | 1 | | | 1 | | 0 | 0 | 0 | 0 | 9 |
| Smith, 2007 ^(257)^ | no statistical analysis | | | | | | | | | | | | | | | | | | 0 | | 0 | 0 | 0 | Over-rule |
| Steel et al, 2007 ^(258)^ | 1 | | 1 | 1 | | 0 | | 1 | | 1 | | 1 | 1 | | 1 | | | 1 | | 0 | 0 | 0 | 0 | 9 |
| Tahrani et al, 2007 ^(259)^ | 1 | | 1 | 1 | | 0 | | 1 | | 1 | | 1 | 1 | | 1 | | | 1 | | 0 | 0 | 0 | 0 | 9 |
| Ting et al, 2007 ^(260)^ | no statistical analysis | | | | | | | | | | | | | | | | | | 0 | | 0 | 0 | 0 | Over-rule |
| Twardella & Brenner, 2007 ^(261)^ | 1 | | 1 | 1 | | 1 | | 1 | | 1 | | 1 | 1 | | 1 | | | 1 | | 1 | 0 | 0 | 0 | 11 |
| Young et al, 2007 ^(262)^ | 1 | | 1 | 1 | | 0 | | 1 | | 1 | | 1 | 1 | | 1 | | | 1 | | 0 | 0 | 0 | 1 | 10 |
| An et al, 2008 ^(263)^ | 1 | | 1 | 1 | | 1 | | 1 | | 1 | | 1 | 1 | | 1 | | | 1 | | 1 | 0 | 0 | 0 | 11 |
| Ashworth et al, 2008 ^(264)^ | 1 | | 1 | 1 | | 0 | | 1 | | 1 | | 1 | 1 | | 1 | | | 1 | | 0 | 0 | 0 | 0 | 9 |
| Bhattacharyya et al, 2008 ^(265)^ | 1 | | 1 | 1 | | 0 | | 1 | | 1 | | 1 | 1 | | 1 | | | 1 | | 0 | 0 | 0 | 0 | 9 |
| Chang et al, 2008 ^(266)^ | too high non response, attrition (> 50%) | | | | | | | | | | | | | | | | | | 0 | | 0 | 0 | 0 | Over-rule |
| Cupples et al, 2008 ^(267)^ | 1 | | 1 | 1 | | 1 | | 1 | | 1 | | 1 | 1 | | 1 | | | 1 | | 0 | 0 | 1 | 0 | 11 |
| Doran et al, 2008a ^(268)^ | 1 | | 1 | 1 | | 0 | | 1 | | 1 | | 1 | 1 | | 1 | | | 1 | | 0 | 0 | 0 | 0 | 9 |
| Doran et al, 2008b ^(269)^ | 1 | | 1 | 1 | | 0 | | 1 | | 1 | | 1 | 1 | | 1 | | | 1 | | 0 | 0 | 0 | 0 | 9 |
| Fleetcroft et al, 2008a ^(270)^ | no significance testing or relationship analysis | | | | | | | | | | | | | | | | | | 0 | | 0 | 0 | 0 | Over-rule |
| Gravelle et al, 2008 ^(271)^ | 1 | | 1 | 1 | | 1 | | 1 | | 1 | | 1 | 1 | | 1 | | | 1 | | 0 | 0 | 0 | 0 | 10 |
| Greenberg et al, 2008 ^(272)^ | 1 | | 1 | 1 | | 0 | | 1 | | 1 | | 1 | 1 | | 1 | | | 1 | | 0 | 0 | 0 | 0 | 9 |
| Gross et al, 2008 ^(273)^ | no statistical analysis | | | | | | | | | | | | | | | | | | 0 | | 0 | 0 | 0 | Over-rule |
| Herrin et al, 2008 ^(274)^ | 1 | | 1 | 1 | | 1 | | 1 | | 1 | | 1 | 1 | | 1 | | | 1 | | 0 | 0 | 1 | 0 | 11 |
| Karve et al, 2008 ^(275)^ | 1 | | 1 | 1 | | 0 | | 1 | | 1 | | 1 | 1 | | 1 | | | 1 | | 0 | 0 | 0 | 0 | 9 |
| Kirschner et al, 2008 ^(276)^ | too small sample (n=11 practices), no significance testing | | | | | | | | | | | | | | | | | | 0 | | 0 | 0 | 0 | Over-rule |
| McBride-Stewart et al, 2008 ^(277)^ | 1 | | 1 | 1 | | 0 | | 1 | | 1 | | 1 | 1 | | 1 | | | 1 | | 0 | 0 | 0 | 1 | 10 |
| McGovern et al, 2008a ^(278)^ | 1 | | 1 | 1 | | 0 | | 1 | | 1 | | 1 | 1 | | 1 | | | 1 | | 0 | 0 | 1 | 0 | 10 |
| McGovern et al, 2008b ^(279)^ | 1 | | 1 | 1 | | 0 | | 1 | | 1 | | 1 | 1 | | 1 | | | 1 | | 0 | 0 | 1 | 0 | 10 |
| Millett et al, 2008a ^(280)^ | 1 | | 1 | 1 | | 0 | | 1 | | 1 | | 1 | 1 | | 1 | | | 1 | | 0 | 0 | 1 | 0 | 10 |
| Millett et al, 2008b ^(281)^ | 1 | | 1 | 1 | | 0 | | 1 | | 1 | | 1 | 1 | | 1 | | | 1 | | 0 | 0 | 1 | 0 | 10 |
| Millett et al, 2008c ^(282)^ | 1 | | 1 | 1 | | 0 | | 1 | | 1 | | 1 | 1 | | 1 | | | 1 | | 0 | 0 | 0 | 0 | 9 |
| Pearson et al, 2008 ^(283)^ | 1 | | 1 | 1 | | 1 | | 1 | | 1 | | 1 | 1 | | -1 | | | 1 | | 0 | 0 | 0 | 0 | 8 |
| Rosenthal et al, 2008 ^(284)^ | 1 | | 1 | 1 | | 1 | | 1 | | 1 | | 1 | 1 | | 1 | | | 1 | | 0 | 0 | 0 | 0 | 10 |
| Steel et al, 2008 ^(285)^ | 1 | | 1 | 1 | | 0 | | 1 | | 1 | | 1 | 1 | | 1 | | | 1 | | 0 | 0 | 0 | 0 | 9 |
| Tahrani et al, 2008 ^(286)^ | 1 | | 1 | 1 | | 0 | | 1 | | 1 | | 1 | 1 | | 1 | | | 1 | | 0 | 0 | 0 | 0 | 9 |
| Vaghela et al, 2008 ^(287)^ | 1 | | 1 | 1 | | 0 | | 1 | | 1 | | 1 | 1 | | 1 | | | 1 | | 0 | 0 | 0 | 0 | 9 |
| Weber et al, 2008 ^(288)^ | 1 | | 1 | 1 | | 0 | | 1 | | 1 | | 1 | 1 | | 1 | | | 1 | | 0 | 0 | 0 | 0 | 9 |
| Yao et al, 2008 ^(289)^ | unclear and small sampling, discorrespondence between text and tables in results interpretation | | | | | | | | | | | | | | | | | | 0 | | 0 | 0 | 0 | Over-rule |
| Benavent et al, 2009 ^(290)^ | Descriptive, no statistical analysis | | | | | | | | | | | | | | | | | | | 0 | 0 | 0 | 0 | Over-rule |
| Bhattacharyya et al, 2009 (291) | 1 | | 1 | 1 | | 0 | | 1 | | 1 | | 1 | 1 | | 1 | | | 1 | | 0 | 0 | 0 | 0 | 9 |
| Calvert et al, 2009 ^(292)^ | 1 | | 1 | 1 | | 0 | | 1 | | 1 | | 1 | 1 | | 1 | | | 1 | | 0 | 0 | 0 | 0 | 9 |
| Campbell et al, 2009 ^(293)^ | 1 | | 1 | 1 | | 0 | | 1 | | 1 | | 1 | 1 | | 1 | | | 1 | | 0 | 0 | 0 | -1 | 8 |
| Crawley et al, 2009 ^(294)^ | 1 | | 1 | 1 | | 0 | | 1 | | 1 | | 1 | 1 | | 1 | | | 1 | | 0 | 0 | 0 | 0 | 9 |
| Damberg et al, 2009 ^(295)^ | Descriptive, no statistical analysis | | | | | | | | | | | | | | | | | | | 0 | 0 | 0 | 0 | Over-rule |
| De Brantes & D’Andrea, 2009 ^(296)^ | 1 | | 1 | 1 | | 0 | | 1 | | 1 | | 1 | 1 | | 1 | | | 1 | | 0 | 0 | 0 | 0 | 9 |
| Foels & Hewner, 2009 ^(297)^ | 1 | | 1 | 1 | | 0 | | 1 | | 1 | | 1 | -1 | | 1 | | | 1 | | 0 | 0 | 0 | 0 | 7 |
| Gemmell et al, 2009 ^(298)^ | 1 | | 1 | 1 | | 0 | | 1 | | 1 | | 1 | 1 | | 1 | | | 1 | | 0 | 0 | 0 | 0 | 9 |
| Menachemi et al, 2009 ^(299)^ | 1 | | 1 | 1 | | 0 | | 1 | | 1 | | 1 | 1 | | 1 | | | 1 | | 0 | 0 | 0 | 0 | 9 |
| Mullen et al, 2009 ^(300)^ | 1 | | 1 | 1 | | 1 | | 1 | | 1 | | 1 | 1 | | 1 | | | 1 | | 0 | 0 | 0 | 1 | 11 |
| Pedros et al, 2009 ^(301)^ | 1 | | 1 | 1 | | 0 | | 1 | | 1 | | 1 | 1 | | 1 | | | 1 | | 0 | 0 | 0 | 1 | 10 |
| Pham et al, 2009 ^(302)^ | 1 | | 1 | 1 | | 0 | | 1 | | 1 | | 1 | 1 | | 1 | | | 1 | | 0 | 0 | 0 | 0 | 9 |
| Robinson et al, 2009 ^(303)^ | 1 | | 1 | 1 | | 0 | | 1 | | 1 | | 1 | 1 | | 1 | | | 1 | | 0 | 0 | 0 | 0 | 9 |
| Rodriguez et al, 2009 ^(304)^ | 1 | | 1 | 1 | | 0 | | 1 | | 1 | | 1 | 1 | | 1 | | | 1 | | 0 | 0 | 0 | 0 | 9 |
| Rubinstein et al, 2009 ^(305)^ | 1 | | 1 | 1 | | 0 | | 1 | | 1 | | 1 | 1 | | 1 | | | 1 | | 0 | 0 | 0 | 0 | 9 |
| Ryan, 2009 ^(306)^ | 1 | | 1 | 1 | | 1 | | 1 | | 1 | | 1 | 1 | | 1 | | | 1 | | 0 | 0 | 0 | 0 | 10 |
| Strong et al, 2009 ^(307)^ | 1 | | 1 | 1 | | 0 | | 1 | | 1 | | 1 | 1 | | 1 | | | 1 | | 1 | 0 | 0 | 0 | 10 |
| Sutton et al, 2009 ^(308)^ | 1 | | 1 | 1 | | 0 | | 1 | | 1 | | 1 | 1 | | 1 | | | 1 | | 0 | 0 | 0 | 0 | 9 |
| Vina et al, 2009 ^(309)^ | 1 | | 1 | 1 | | 0 | | 1 | | 1 | | 1 | 1 | | 1 | | | 1 | | 0 | 1 | 0 | 0 | 10 |

Legend: The tool consists of ten generic items (namely research question, patient population and setting, intervention, comparison, outcome, design, sample size, statistics, generalisability, confounders addressed) and four design specific items (randomization, blinding, clustering effect, number of data points). For each primary study, a score has been given to each of the 14 items, ranging from -1 to +1. A score of +1 on a certain item corresponds with ‘a good result’, a score of -1 corresponds with ‘a bad result’, and a score of 0 corresponds with ‘this item has not been discussed in the article’. To pass the quality appraisal, an article has to gain a score of minimum 8 out of 14. An article can be excluded on the basis of an ‘overrule’ argument when a certain criteria was not met, hence the overall validity has to be questioned (for example no significance testing, too small sample size etc…).

**eTable 7a: M**odelling studies quality appraisal

| Citation | **according to farmacoeconomic guidelines KCE: guideline 10: Modelling + ISPOR guidelines** | | | | | | |  |
| --- | --- | --- | --- | --- | --- | --- | --- | --- |
|  | model design (as simple as possible) | assumptions tested in sensitivity analysis/scenario analysis | original data set provided | sources used presented and described in detail (high quality) | scenarios (for models that extrapolate to longer time periods) | calibration (results should be logically consistent with real-life observations and data) | face validity (the results of the model should be intuitively correct) and cross-validation (transparant enough to allow an explanation of the differences with other models for the same intervention) |  |
| Kahn et al, 2006 ^(310)^ | +/- | - | + | + | NA | + | + |  |
| Fleetcroft & Cookson, 2006 ^(311)^ | + | - | + | + | - | + | + |  |
| McElduff et al, 2004 ^(312)^ | + | + | + | + | - | + (if proposed targets are achieved) | + |  |
| fleetcroft et al, 2008 ^(313)^ | + | + | +/- | + | - | + | + |  |

**eTable 7b: Cost-effectiveness studies quality appraisal**

| Citation | **according to farmacoeconomic guidelines KCE** | | | | | | | | | | | | | |
| --- | --- | --- | --- | --- | --- | --- | --- | --- | --- | --- | --- | --- | --- | --- |
|  | literature review | perspective of the evaluation | taget population | comparator | analytic technique | study design | calculation of costs | valuation of outcomes | data source | incremental cost-effectiveness | time horizon | modelling | handling uncertainty | discount rate |
| Nahra et al, 2006 ^(314)^ | +/- | + | + | NA (new method already in use) | cost-utility | + | +/-(cost of treatment not included) | + | + | NA (old method no costs) | NA | + | + | + (5%) |
| Curtin et al, 2006 ^(315)^ | - | + | + | + | cost-benefit | +/- | + | + | + | NA (old method no costs) | NA | NA | NA | NA |
| Mason et al, 2008 ^(316)^ | +/- | + | + | +/- | cost-utility | + | +/- | + | +/- | NA (old method no costs) | NA | + | + | +/- |
| Salize et al, 2009 ^(317)^ | +/- | + | + | + | cost-utility | + | + | + | + | + | NA | + | + | +/- |

Legend: For each item one of the following scores have been assigned: +, +/-, -. A ‘+’ score on given item corresponds with a good result, a ‘-‘ score corresponds with a bad result, a ‘+/-‘ score corresponds with an in between result. Given the limited amount of modelling and cost-effectiveness studies, the quality appraisal is only performed to inform the reader about the quality of the studies, but was not used to exclude any study. Overall the quality of the modelling and cost-effectiveness studies was acceptable.

**eTable 8: Description of included studies**

| **Author(s)** | **Publica-tion year** | **Country** | **Setting** | | **Study period** | **Study design** | **Sample size** | | |
| --- | --- | --- | --- | --- | --- | --- | --- | --- | --- |
|  |  |  | **Pri-mary** | **Hos-pital** |  |  | **Organizations/**  **practices** | **Providers** | **Patients** |
| Ritchie et al ^(144)^ | 1992 | UK | X |  | 1990-1991 | Historic, Multi | 95 | 313 | 6600 vs. 6400 |
| Kouides et al ^(145)^ | 1993 | US | X | X | 1990-1991 | Concurrent + Historic | NR | 53 vs. 82 | 12271 vs. 30387 |
| Langham et al ^(318)^ | 1995 | UK | X |  | 1992-1993 | Concurrent + Historic | 78 vs. 85 | NR | NR |
| Lynch ^(147)^ | 1995 | UK | X |  | 1991-1992 | Cross section | 208 | NR | NR |
| Morrow et al ^(148)^ | 1995 | US | X |  | 1987-1990 | Historic, Multi | 1607, 418, 271 | NR | 50 per practice |
| Fairbrother et al ^(149)^ | 1997 | US | X |  | 1993-1996 | Historic, before-after | NR | 23 | 173 vs. 528 |
| Grady et al ^(150)^ | 1997 | US | X |  | 3 years | Randomized | 21 vs. 21 vs. 23 | 109 | 11716 |
| Hillman et al ^(151)^ | 1998 | US | X |  | 1993-1995 | Randomized | 26 vs. 26 | NR | NR |
| Kouides et al ^(152)^ | 1998 | US | X |  | 1990-1991 | Randomized | 54 | NR | NR |
| Cameron et al ^(153)^ | 1999 | Australia |  | X | 1991-1997 | Historic, before-after | 21 | NR | NR |
| Fairbrother et al ^(154)^ | 1999 | US | X |  | 1995-1996 | Randomized | NR | 60 | 50 per provider |
| Hillman et al ^(155)^ | 1999 | US | X |  | 1993-1995 | Randomized | 49 | NR | 15 per practice |
| Lebaron ^(157)^ | 1999 | US | X |  | NR | Cross section | 73-116 per state (4), 8-25 per city (2) | NR | 4639-18000, 714-5276 per clinic |
| Safran et al ^(160)^ | 2000 | US | X |  | 1996 | Cross section | NR | NR | 6018 |
| Cattaneo et al ^(162)^ | 2000 | Italy |  | X | 1998-1999 | Historic, before-after | 10 | NR | 9264 |
| Fairbrother et al ^(164)^ | 2001 | US | X |  | 1997-1998 | Randomized | NR | 57 | 50 per provider |
| Shortell et al ^(165)^ | 2001 | US | X |  | NR | Cross section | 56 | 1797 | NR |
| Bond et al ^(168)^ | 2002 | Australia | X |  | 1997-2000 | Cross section | 47 | NR | 1578 vs. 1793 |
| Amundson et al ^(169)^ | 2003 | US | X |  | 1996-1997 | Historic, Multi | 20 | NR | 14489 |
| Casalino et al ^(170)^ | 2003 | US | X |  | 2000-2001 | Cross section | 1587 | NR | NR |
| Chung et al ^(171)^ | 2003 | US | X |  | 1997-2000 | Historic, Multi | NR | 1600 | NR |
| Larsen et al ^(172)^ | 2003 | US | X | X | 1998-2002 | Concurrent + Historic | NR | NR | 5785-9463 per year |
| McMenamin et al ^(173)^ | 2003 | US | X | X | 2000-2001 | Cross section | 1104 | NR | NR |
| Roski et al ^(174)^ | 2003 | US | X |  | 1999-2000 | Randomized | 15 vs. 15 vs. 10 | NR | 4813 vs. 4734 |
| Armour et al ^(175)^ | 2004 | US | X |  | 2000-2001 | Historic, before-after | NR | NR | 3058 vs. 3691 |
| Greene et al ^(178)^ | 2004 | US | X | X | 1999-2001 | Historic, before-after | NR | 900 | 96766 |
| Hippisley-Cox et al ^(179)^ | 2004 | UK | X |  | 2004 | Cross section | 237 | NR | 53687 |
| Li et al ^(181)^ | 2004 | US | X | X | 2000-2001 | Cross section | 1104 | NR | NR |
| McElduff et al ^(312)^ | 2004 | UK | X |  | 2004 | Modelling | NA | NA | NA |
| McMenamin et al ^(182)^ | 2004 | US | X | X | 2000-2001 | Cross section | 1104 | NR | NR |
| Schmittdiel et al ^(183)^ | 2004 | US | X | X | 2000-2001 | Cross section | 1104 | NR | NR |
| Ashworth et al ^(185)^ | 2005 | UK | X |  | 2001-2002 | Cross section | 151 | NR | NR |
| Beaulieu & Horrigan ^(186)^ | 2005 | US | X |  | 2001-2002 | Concurrent + Historic | NR | 21 | 624 vs. 600 |
| Pourat et al ^(190)^ | 2005 | US | X |  | 2002 | Cross section | NR | 948 | NR |
| Rosenthal et al ^(193)^ | 2005 | US | X |  | 2003-2004 | Concurrent + Historic | 134 vs. 33 | NR | NR |
| Shenkman et al ^(194)^ | 2005 | US | X |  | 1999-2001 | Cross section | NR | NR | 2333 |
| Curtin et al ^(315)^ | 2006 | US | X |  | 2000-2004 | Modelling | NR | 3700 | NR |
| Doran et al ^(197)^ | 2006 | UK | X |  | 2004-2005 | Cross section | 8105 | NR | NR |
| Ettner et al ^(198)^ | 2006 | US | X |  | 2000-2001 | Cross section | NR | NR | 6194 |
| Fleetcroft & Cookson ^(311)^ | 2006 | UK | X |  | 2004 | Modelling | NA | NA | NA |
| Grossbart ^(200)^ | 2006 | US |  | X | 2003-2004 | Concurrent + Historic | 4 vs. 6 | NR | 4964 and 6025 vs. 8641 and 9295 |
| Jaiveer et al ^(202)^ | 2006 | UK | X |  | 2004-2005 | Historic, before-after | 13 | NR | 3453 vs. 3173 |
| Kahn et al ^(310)^ | 2006 | US |  | X | 2005 | Modelling | 4203 | NR | NR |
| Levin-Scherz et al ^(203)^ | 2006 | US | X | X | 2001-2003 | Concurrent + Historic | 8 | 5100 | NR |
| McLean et al ^(204)^ | 2006 | UK | X |  | 2004 | Concurrent | 1024 | NR | NR |
| Nahra et al ^(314)^ | 2006 | US |  | X | 2000-2003 | Modelling | 85 | NR | NR |
| Reiter et al ^(207)^ | 2006 | US |  | X | NR | Cross section | 66 | NR | NR |
| Reschovsky et al ^(208)^ | 2006 | US | X | X | 2000-2001 | Cross section | NR | 12406 | NR |
| Rittenhouse & Robinson ^(209)^ | 2006 | US | X |  | 2003 | Cross section | 123 | NR | NR |
| Sigfrid et al ^(210)^ | 2006 | UK | X |  | 2004-2005 | Cross section | 49 | NR | 7157 |
| Simpson et al ^(211)^ | 2006 | UK | X |  | 2004-2005 | Historic, before-after | 310 | NR | 21901 vs. 32401 |
| Srilangalingam et al ^(212)^ | 2006 | UK | X |  | 2003-2004 | Historic, before-after | NR | NR | 328 vs. 319 |
| Strong et al ^(213)^ | 2006 | UK | X |  | 2004-2005 | Cross section | 38 | NR | 12920 |
| Sutton & McLean ^(214)^ | 2006 | UK | X |  | 2004-2005 | Cross section | 60 | NR | NR |
| Wang et al ^(216)^ | 2006 | UK | X |  | 2005 | Cross section | 638 | NR | NR |
| Williams et al (a) ^(218)^ | 2006 | US | X |  | 2003-2004 | Historic, before-after | 225 | NR | More than 35000 |
| Williams et al (b) ^(219)^ | 2006 | UK | X |  | 2004 | Cross section | 2 | NR | 11109 and 9557 |
| Wright et al ^(220)^ | 2006 | UK | X |  | 2004-2005 | Cross section | NR | NR | 8569 |
| Ashworth et al (a) ^(221)^ | 2007 | UK | X |  | 2004-2005 | Cross section | 8430 | NR | NR |
| Ashworth et al (b) ^(222)^ | 2007 | UK | X |  | 2004-2006 | Historic, before-after | 8515 vs. 8264 | NR | NR |
| Campbell et al ^(223)^ | 2007 | UK | X |  | 1998, 2003, 2005 | Historic, Multi | 42 | NR | 2300, 1495, 1482 |
| Casale et al ^(225)^ | 2007 | US |  | X | 2006-2007 | Historic, Multi | NR | NR | 137 vs. 117 |
| Coleman et al (a) ^(226)^ | 2007 | US | X |  | 2002-2004 | Concurrent + Historic | NR | 46 | 1166 |
| Coleman et al (b) ^(227)^ | 2007 | UK | X |  | 1990-2005 | Historic, Multi | NR | NR | 384259 per year |
| Downing et al ^(228)^ | 2007 | UK | X |  | 2004-2005 | Cross section | 94 | NR | NR |
| Gene-Badia et al ^(230)^ | 2007 | Spain | X |  | 2002-2003 | Historic, before-after | 257 | 3439 and 3781 | 200 per PCT |
| Gilmore et al ^(231)^ | 2007 | US | X |  | 1998-2003 | Concurrent + Historic | NR | NR | 222213 per year |
| Glickman et al ^(232)^ | 2007 | US |  | X | 2003-2006 | Concurrent + Historic | 54 vs. 446 | NR | 105383 |
| Gray et al ^(233)^ | 2007 | UK | X |  | 2005-2006 | Cross section | 32 | NR | 7605 |
| Gulliford et al ^(234)^ | 2007 | UK | X |  | 2005 | Cross section | 8484 | NR | NR |
| Lindenauer et al ^(240)^ | 2007 | US |  | X | 2003-2005 | Concurrent + Historic | 207 vs. 406 | NR | NR |
| McLean ^(243)^ | 2007 | UK | X |  | 2003-2005 | Cross section | 8214 vs. 1023 vs. 362 vs. 459 | NR | NR |
| McLean et al ^(244)^ | 2007 | UK | X |  | 2004 | Cross section | 912 | NR | NR |
| Mehrotra ^(245)^ | 2007 | US | X |  | 2005 | Cross section | 100 | NR | NR |
| Millett et al (a) ^(246)^ | 2007 | UK | X |  | 2003-2005 | Historic, Multi | 32 | NR | 4284 |
| Millett et al (b) ^(247)^ | 2007 | UK | X |  | 2003-2006 | Historic, Multi | 32 | NR | 4284 |
| Millett et al (c) ^(248)^ | 2007 | UK | X |  | NR (after 2004) | Cross section | 8970 | NR | 1852762 |
| O’Malley et al ^(250)^ | 2007 | US | X | X | 1996-2005 | Historic, Multi | 7057 vs. 8487 | NR | NR |
| Saxena et al ^(253)^ | 2007 | UK | X |  | 2004-2005 | Cross section | 8970 | NR | 2039919, 8970, 6300476, 839758 |
| Shohet et al ^(254)^ | 2007 | UK | X |  | 2004-2005 | Cross section | 291 | NR | 14224 |
| Simon et al ^(255)^ | 2007 | US | X | X | 2000-2001 | Cross section | 1104 | NR | NR |
| Simpson et al ^(256)^ | 2007 | UK | X |  | 2005 | Cross section | 310 | NR | 32401 |
| Steel et al ^(258)^ | 2007 | UK | X |  | 2003-2005 | Historic, before-after | 18 | NR | 586 vs. 570 |
| Tahrani et al ^(259)^ | 2007 | UK | X |  | 2004-2006 | Historic, Multi | 66 | NR | 16867 |
| Twardella & Brenner ^(261)^ | 2007 | Germany | X |  | 2002-2004 | Randomized | 82 | 94 | 577 |
| Young et al ^(262)^ | 2007 | US | X |  | 1999-2004 | Concurrent + Historic | NR | 334 | NR |
| An et al ^(263)^ | 2008 | US | X |  | 2005-2006 | Randomized | 25 vs. 24 | NR | NR |
| Ashworth et al ^(264)^ | 2008 | UK | X |  | 2004-2007 | Historic, Multi | 8515, 8264, 8192 | NR | NR |
| Bhattacharyya et al ^(265)^ | 2008 | US |  | X | NR | Cross section | 257 | NR | NR |
| Cupples et al ^(267)^ | 2008 | UK | X |  | 2004-2006 | Cross section | 16 vs. 32 | NR | 350 vs. 648 |
| Doran et al (a) ^(268)^ | 2008 | UK | X |  | 2004-2006 | Cross section | 7637 | NR | NR |
| Doran et al (b) ^(319)^ | 2008 | UK | X |  | 2005-2006 | Cross section | 8105 | NR | NR |
| Fleetcroft et al ^(313)^ | 2008 | UK | X |  | 2004, 2006 | Modelling | NA | NA | NA |
| Gravelle et al ^(271)^ | 2008 | UK | X |  | 2004-2005 | Concurrent | 916 | NR | NR |
| Greenberg et al ^(272)^ | 2008 | US |  | X | 2001-2003 | Cross section | 1 | 63 | 150 |
| Herrin et al ^(274)^ | 2008 | US |  | X | 2001-2005 | Concurrent + Historic | 5 vs. 200 | NR | 13673 |
| Karve et al ^(275)^ | 2008 | US |  | X | 2004-2005 | Cross section | 3449 | NR | NR |
| MacBride-Stewart et al ^(277)^ | 2008 | UK | X |  | 2002-2006 | Historic, Multi | 92 | NR | NR |
| Mason et al ^(316)^ | 2008 | UK | X |  | 2004-2007 | Modelling | NA | NA | NA |
| McGovern et al (a) ^(278)^ | 2008 | UK | X |  | 2004-2005 | Historic, before-after | 310 | NR | 37329 vs. 56561 |
| McGovern et al (b) ^(279)^ | 2008 | UK | X |  | 2004-2005 | Historic, before-after | 310 | NR | 58406 vs. 75495 |
| Millett et al (a) ^(280)^ | 2008 | UK | X |  | 2000-2005 | Historic, Multi | 15 | NR | 1968 |
| Millett et al (b) ^(281)^ | 2008 | UK | X |  | 2003-2005 | Historic, before-after | 32 | NR | 2891 vs. 3101 |
| Millett et al (c)  ^(282)^ | 2008 | UK | X |  | 2005-2006 | Historic, before-after | 16 | NR | 8876 |
| Pearson et al ^(283)^ | 2008 | US | X |  | 2001-2003 | Concurrent + Historic | 154 | 5350 | NR |
| Rosenthal et al ^(284)^ | 2008 | US | X | X | 2003-2006 | Concurrent + Historic | NR | 405 vs. 3916, 91 vs. 1204 | NR |
| Steel et al ^(285)^ | 2008 | UK | X |  | 2004-2005 | Concurrent | NR | NR | 4417 |
| Tahrani et al ^(286)^ | 2008 | UK | X |  | 2004-2006 | Historic, Multi | 66 | NR | 16858 |
| Vaghela et al ^(287)^ | 2008 | UK | X |  | 2004-2008 | Historic, Multi | 8423, 8264, 8192, 8255 | NR | NR |
| Weber et al ^(288)^ | 2008 | US | X |  | 2006-2007 | Historic, Multi | NR | 124-136 | 18511-19494 |
| Bhattacharyya et al ^(291)^ | 2009 | US |  | X | 2003 | Cross section | 260 | NR | NR |
| Calvert et al ^(292)^ | 2009 | UK | X |  | 2001-2007 | Historic, Multi | 147 | NR | NR |
| Campbell et al ^(293)^ | 2009 | UK | X |  | 1998-2007 | Historic, Multi | 42 | NR | NR |
| Crawley et al ^(294)^ | 2009 | UK | X |  | 2003, 2006 | Historic, before-after | NR | NR | 5189 vs. 4079 |
| De brantes & D’ Andrea ^(296)^ | 2009 | US | X |  | NR | Cross section | NR | 582-7716 per region | NR |
| Gemmell et al ^(298)^ | 2009 | UK | X |  | 2003-2005 | Historic, before-after | 42 | NR | NR |
| Menachemi et al ^(299)^ | 2009 | US | X |  | 2005 | Cross section | NR | 1014 | NR |
| Mullen et al ^(300)^ | 2009 | US | X |  | 2000-2004 | Concurrent + Historic | 172 vs. 242 | NR | NR |
| Pedros et al ^(301)^ | 2009 | Spain |  | X | 1998-2005 | Historic, Multi | 1 | All physicians in one hospital | NR |
| Pham et al ^(302)^ | 2009 | US | X |  | 2000-2002, 2004-2006 | Cross section | NR | 4567 | 35039 |
| Robinson et al ^(303)^ | 2009 | US | X | X | 2006-2007 | Cross section | 538 | NR | NR |
| Rodriguez et al ^(304)^ | 2009 | US | X |  | 2005-2006 | Cross section | 34 | 2099 | 105663 |
| Rubinstein et al ^(305)^ | 2009 | Argentina | X |  | 2005-2007 | Historic, before-after | 18 | NR | 45700 |
| Ryan ^(306)^ | 2009 | US |  | X | 2000-2006 | Concurrent + Historic | 3570 | NR | 6713928 |
| Salize et al ^(317)^ | 2009 | Germany | X |  | 2002-2004 | Modelling | NA | NA | NA |
| Strong et al ^(307)^ | 2009 | UK | X |  | 2006-2007 | Cross section | 38 | NR | 3217 |
| Sutton et al ^(308)^ | 2009 | UK | X |  | 2000-2005 | Concurrent + Historic | 315 | NR | NR |
| Vina et al ^(309)^ | 2009 | US |  | X | 2004-2005 | Cross section | 92 | NR | NR |

Legend: ‘NA’ stands for Not applicable; ‘NR’ stands for Not reported.

# References

(1) Chen GJ, Feldman SR. Economic aspect of health care systems. Advantage and disadvantage incentives in different systems. Dermatol Clin 2000; 18(2):211-214.

(2) Gosden T, Forland F, Kristiansen IS, Sutton M, Leese B, Giuffrida A et al. Capitation, salary, fee-for-service and mixed systems of payment: effects on the behaviour of primary care physicians. Cochrane Database Syst Rev 2000;(3):CD002215.

(3) Baker LC. Managed care spillover effects. Annual Review of Public Health 2003; 24:435-456.

(4) Armour BS, Pitts MM. Physician financial incentives in managed care - Resource use, quality and cost implications. Disease Management & Health Outcomes 2003; 11(3):139-147.

(5) Havranek EP, Krumholz HM, Dudley RA, Adams K, Gregory D, Lampert S et al. Aligning quality and payment for heart failure care: defining the challenges. J Card Fail 2003; 9(4):251-254.

(6) Yabroff KR, Mangan P, Mandelblatt J. Effectiveness of interventions to increase Papanicolaou smear use. J Am Board Fam Pract 2003; 16(3):188-203.

(7) Baily MA. Ethics, economics, and physician reimbursement. Mt Sinai J Med 2004; 71(4):231-235.

(8) Borenstein J, Badamgarav E, Henning JM, Gano AD, Weingarten SR. The association between quality improvement activities performed by managed care organizations and quality of care. American Journal of Medicine 2004; 117(5):297-304.

(9) Mojica WA, Suttorp MJ, Sherman SE, Morton SC, Roth EA, Maglione MA et al. Smoking-cessation interventions by type of provider - A meta-analysis. American Journal of Preventive Medicine 2004; 26(5):391-401.

(10) Arnold SR, Straus SE. Interventions to improve antibiotic prescribing practices in ambulatory care. Cochrane Database Syst Rev 2005;(4):CD003539.

(11) Harris I, Mulford J, Solomon M, van Gelder JM, Young J. Association between compensation status and outcome after surgery: a meta-analysis. JAMA 2005; 293(13):1644-1652.

(12) Shortell SM, Schmittdiel J, Wang MC, Li R, Gillies RR, Casalino LP et al. An empirical assessment of high-performing medical groups: Results from a national study. Medical Care Research and Review 2005; 62(4):407-434.

(13) Chaillet N, Dube E, Dugas M, Audibert F, Tourigny C, Fraser WD et al. Evidence-based strategies for implementing guidelines in obstetrics: a systematic review. Obstet Gynecol 2006; 108(5):1234-1245.

(14) Veloski J, Boex JR, Grasberger MJ, Evans A, Wolfson DB. Systematic review of the literature on assessment, feedback and physicians' clinical performance: BEME Guide No. 7. Medical Teacher 2006; 28(2):117-128.

(15) Yen BM. Engaging physicians to change practice. Journal of Clinical Outcomes Management 2006; 13(2):103-110.

(16) Khunti K, Gadsby R, Millett C, Majeed A, Davies M. Quality of diabetes care in the UK: comparison of published quality-of-care reports with results of the Quality and Outcomes Framework for Diabetes. Diabetic Medicine 2007; 24(12):1436-1441.

(17) Sood R, Sood A, Ghosh AK. Non-evidence-based variables affecting physicians' test-ordering tendencies: a systematic review. Netherlands Journal of Medicine 2007; 65(5):167-177.

(18) Akbari A, Mayhew A, Al Alawi MA, Grimshaw J, Winkens R, Glidewell E et al. Interventions to improve outpatient referrals from primary care to secondary care. Cochrane Database Syst Rev 2008;(4):CD005471.

(19) Bremer RW, Scholle SH, Keyser D, Houtsinger JVK, Pincus HA. Pay for Performance in Behavioral Health. Psychiatric Services 2008; 59(12):1419-1429.

(20) Chan P, Grindrod K, Bougher D, Passuto FM, Wilgosh C, Eberhart G et al. A systematic review of remuneration systems for clinical pharmacy care services. Can Pharn J 2008; 141(2):102-112.

(21) Glickman SW, Schulman KA, Peterson ED, Hocker MB, Cairns CB. Evidence-based perspectives on pay for performance and quality of patient care and outcomes in emergency medicine. Annals of Emergency Medicine 2008; 51(5):622-631.

(22) Kaestner R, Guardado J. Medicare reimbursement, nurse staffing, and patient outcomes. J Health Econ 2008; 27(2):339-361.

(23) Lu CY, Ross-Degnan D, Soumerai SB, Pearson SA. Interventions designed to improve the quality and efficiency of medication use in managed care: A critical review of the literature - 2001-2007. Bmc Health Services Research 2008; 8.

(24) Raftery J, Bryant J, Powell J, Kerr C, Hawker S. Payment to healthcare professionals for patient recruitment to trials: systematic review and qualitative study. Health Technol Assess 2008; 12(10):1-128, iii.

(25) Welton JM. Implications of Medicare reimbursement changes related to inpatient nursing care quality. J Nurs Adm 2008; 38(7-8):325-330.

(26) Briesacher BA, Field TS, Baril J, Gurwitz JH. Pay-for-Performance in Nursing Homes. Health Care Financing Review 2009; 30(3):1-13.

(27) Scott I. What are the most effective strategies for improving quality and safety of health care? Intern Med J 2009; 39(6):389-400.

(28) Sarnoff R, Rundall T. Meta-analysis of effectiveness of interventions to increase influenza immunization rates among high-risk population groups. Med Care Res Rev 1998; 55(4):432-456.

(29) Achat H, McIntyre P, Burgess M. Health care incentives in immunisation. Australian and New Zealand Journal of Public Health 1999; 23(3):285-288.

(30) Buchan J, Thompson M, O'May F. Issues in health service delivery. Discussion Paper Nr. 4. Incentive and remuneration strategies. Health workforce incentives and remuneration strategies. A research review. WHO/EIP/OSD/00.14. 2000. Evidence and information for policy. Department of organization of healthservices delivery. World Health Organization. Geneva.

Ref Type: Report

(31) Chaix-Couturier C, Durand-Zaleski I, Jolly D, Durieux P. Effects of financial incentives on medical practice: results from a systematic review of the literature and methodological issues. Int J Qual Health Care 2000; 12(2):133-142.

(32) Giuffrida A, Gosden T, Forland F, Kristiansen IS, Sergison M, Leese B et al. Target payments in primary care: effects on professional practice and health care outcomes. Cochrane Database Syst Rev 2000;(3):CD000531.

(33) Armour BS, Pitts MM, Maclean R, Cangialose C, Kishel M, Imai H et al. The effect of explicit financial incentives on physician behavior. Arch Intern Med 2001; 161(10):1261-1266.

(34) Heffner JE. Altering physician behavior to improve clinical performance. Top Health Inf Manage 2001; 22(2):1-9.

(35) Flynn KE, Smith MA, Davis MK. From physician to consumer: The effectiveness of strategies to manage health care utilization. Medical Care Research and Review 2002; 59(4):455-481.

(36) Stone EG, Morton SC, Hulscher ME, Maglione MA, Roth EA, Grimshaw JM et al. Interventions that increase use of adult immunization and cancer screening services: a meta-analysis. Ann Intern Med 2002; 136(9):641-651.

(37) Grol R, Grimshaw J. From best evidence to best practice: effective implementation of change in patients' care. Lancet 2003; 362(9391):1225-1230.

(38) alper PR. Pitfalls of financial incentives for improving diabetes outcomes. Diabetes trends 2004; 16S:36-40.

(39) Conrad DA, Christianson JB. Penetrating the "black box": financial incentives for enhancing the quality of physician services. Med Care Res Rev 2004; 61(3 Suppl):37S-68S.

(40) Czubak R, Tucker J, Zarowitz BJ. Optimizing drug prescribing in managed care populations - Improving clinical and economic outcomes. Disease Management & Health Outcomes 2004; 12(3):147-167.

(41) Dudley RA, Frolich A, Robinowitz DL, Talavera JA, Broadhead P, Luft HS. Strategies to support quality-based purchasing: A review of the evidence. Technical review 10. AHRQ Publication No. 04-0057. 2004. Rockville, MD, Prepared by the Stanford-University of California San Francisco Evidence-based Practice Center.

Ref Type: Report

(42) Kane RL, Johnson PE, Town RJ, Butler M. Economic incentives for preventive care. Evid Rep Technol Assess (Summ ) 2004;(101):1-7.

(43) Shortell SM. Increasing value: a research agenda for addressing the managerial and organizational challenges facing health care delivery in the United States. Med Care Res Rev 2004; 61(3 Suppl):12S-30S.

(44) Smellie WSA, Roy DV. Impact of the new General Medical Services contract on the clinical laboratory. Annals of Clinical Biochemistry 2005; 42:4-10.

(45) Sturm H, Austvoll-Dahlgren A, Aaserud M, Oxman AD, Ramsay C, Vernby A et al. Pharmaceutical policies: effects of financial incentives for prescribers. Cochrane Database Syst Rev 2007;(3):CD006731.

(46) Town R, Kane R, Johnson P, Butler M. Economic incentives and physicians' delivery of preventive care: a systematic review. Am J Prev Med 2005; 28(2):234-240.

(47) Fenter TC, Lewis SJ. Pay-for-performance initiatives. Journal of Managed Care Pharmacy 2008; 14(6):S12-S15.

(48) Freed GL, Uren RL. Pay-for-performance: an overview for pediatrics. J Pediatr 2006; 149(1):120-124.

(49) McNamara P. Purchaser strategies to influence quality of care: from rhetoric to global applications. Qual Saf Health Care 2006; 15(3):171-173.

(50) Petersen LA, Woodard LD, Urech T, Daw C, Sookanan S. Does pay-for-performance improve the quality of health care? Ann Intern Med 2006; 145(4):265-272.

(51) Pink GH, Brown AD, Studer ML, Reiter KL, Leatt P. Pay-for-performance in publicly financed healthcare: some international experience and considerations for Canada. Healthc Pap 2006; 6(4):8-26.

(52) Rosenthal MB, Frank RG. What is the empirical basis for paying for quality in health care? Medical Care Research and Review 2006; 63(2):135-157.

(53) Chien AT, Conti RM, Pollack HA. A pediatric-focused review of the performance incentive literature. Curr Opin Pediatr 2007; 19(6):719-725.

(54) Doran T, Fullwood C. Pay for performance: is it the best way to improve control of hypertension? Curr Hypertens Rep 2007; 9(5):360-367.

(55) Ensor T, Weinzierl S. Regulating health care in low- and middle-income countries: Broadening the policy response in resource constrained environments. Social Science & Medicine 2007; 65(2):355-366.

(56) Frolich A, Talavera JA, Broadhead P, Dudley RA. A behavioral model of clinician responses to incentives to improve quality. Health Policy 2007; 80(1):179-193.

(57) Gonzalez CM, Penson D, Kosiak B, Dupree J, Clemens JQ. Pay for performance: rationale and potential implications for urology. J Urol 2007; 178(2):402-408.

(58) Hartig JR, Allison J. Physician performance improvement: an overview of methodologies. Clin Exp Rheumatol 2007; 25(6 Suppl 47):50-54.

(59) Pierce RG, Bozic KJ, Bradford DS. Pay for performance in orthopaedic surgery. Clin Orthop Relat Res 2007; 457:87-95.

(60) Scott IA. Pay for performance in health care: strategic issues for Australian experiments. Med J Aust 2007; 187(1):31-35.

(61) Sikka R. Pay for performance in emergency medicine. Annals of Emergency Medicine 2007; 49(6):756-761.

(62) Varela G. Pay for performance in thoracic surgery. Thorac Surg Clin 2007; 17(3):431-435.

(63) Chopra M, Munro S, Lavis JN, Vist G, Bennett S. Effects of policy options for human resources for health: an analysis of systematic reviews. Lancet 2008; 371(9613):668-674.

(64) Christianson JB, Leatherman S, Sutherland K. Lessons From Evaluations of Purchaser Pay-for-Performance Programs A Review of the Evidence. Medical Care Research and Review 2008; 65(6):5S-35S.

(65) Curry SJ, Keller PA, Orleans CT, Fiore MC. The role of health care systems in increased tobacco cessation. Annu Rev Public Health 2008; 29:411-428.

(66) Custers T, Hurley J, Klazinga NS, Brown AD. Selecting effective incentive structures in health care: A decision framework to support health care purchasers in finding the right incentives to drive performance. BMC Health Serv Res 2008; 8:66.

(67) Hamblin R. Regulation, measurements and incentives. The experience in the US and UK: does context matter? J R Soc Promot Health 2008; 128(6):291-298.

(68) Hart-Hester S, Jones W, Watzlaf VJ, Fenton SH, Nielsen C, Madison M et al. Impact of creating a pay for quality improvement (P4QI) incentive program on healthcare disparity: leveraging HIT in rural hospitals and small physician offices. Perspect Health Inf Manag 2008; 5:14.

(69) Mason A. New medicines in primary care: a review of influences on general practitioner prescribing. Journal of Clinical Pharmacy and Therapeutics 2008; 33(1):1-10.

(70) McDonald J, Harris MF, Cumming J, Davies GP, Burns P. The implementation and impact of different funding initiatives on access to multidisciplinary primary health care and policy implications. Medical Journal of Australia 2008; 188(8):S69-S72.

(71) Sabatino SA, Habarta N, Baron RC, Coates RJ, Rimer BK, Kerner J et al. Interventions to increase recommendation and delivery of screening for breast, cervical, and colorectal cancers by healthcare providers - Systematic reviews of provider assessment and feedback and provider incentives. American Journal of Preventive Medicine 2008; 35(1):S67-S74.

(72) Schatz M. Does pay-for-performance influence the quality of care? Curr Opin Allergy Clin Immunol 2008; 8(3):213-221.

(73) Thomas C, Rosenthal MB. The medical home: Growing evidence to support a new approach to primary care. Journal of the American Board of Family Medicine 2008; 21(5):427-440.

(74) Sloan FA, Kasper H. Incentives and choice in health care. Cambridge, Massachusetts London, England: MIT Press; 2008.

(75) Conrad DA, Perry L. Quality-based financial incentives in health care: can we improve quality by paying for it? Annu Rev Public Health 2009; 30:357-371.

(76) Greene SE, Nash DB. Pay for Performance: An Overview of the Literature. Am J Med Qual 2009.

(77) James CV, Rosenbaum S. Paying for quality care: implications for racial and ethnic health disparities in pediatric asthma. Pediatrics 2009; 123 Suppl 3:S205-S210.

(78) Khanduja K, Scales DC, Adhikari NK. Pay for performance in the intensive care unit--opportunity or threat? Crit Care Med 2009; 37(3):852-858.

(79) Mehrotra A, Damberg CL, Sorbero MES, Teleki SS. Pay for Performance in the Hospital Setting: What Is the State of the Evidence? American Journal of Medical Quality 2009; 24(1):19-28.

(80) Rhoads KF, Koneti BM, Dudley RA. Performance management, public reporting and pay-for-performance. Urologic Clinics of North America 2009; 36:37-48.

(81) Hemenway D. Financial Incentives for Childhood Immunization. Journal of Policy Analysis and Management 1995; 14(1):133-139.

(82) Shen YJ. Selection incentives in a performance-based contracting system. Health Services Research 2003; 38(2):535-552.

(83) Dixon J, Lewis R, Rosen R, Finlayson B, Gray D. Can the NHS learn from US managed care organisations? British Medical Journal 2004; 328(7433):223-225.

(84) Eggleston K, Hsieh CR. Healthcare payment incentives : a comparative analysis of reforms in taiwan, South Korea and china. Appl Health Econ Health Policy 2004; 3(1):47-56.

(85) Feldman S. Rewarding results: Improving the quality of treatment for people with alcohol and drug problems - Join together, a national policy panel. Administration and Policy in Mental Health 2004; 31(4):283-312.

(86) Forsberg E, Axelsson R, Arnetz B. The relative importance of leadership and payment system - Effects on quality of care and work environment. Health Policy 2004; 69(1):73-82.

(87) Laurence CO, Beilby J, Campbell S, Campbell J, Ponte L, Woodward G. Process for improving the integration of care across the primary and acute care settings in rural South Australia: asthma as a case study. Aust J Rural Health 2004; 12(6):264-268.

(88) Mullen PM. Using performance indicators to improve performance. Health Services Management Research 17, 217-228. 2004.

Ref Type: Journal (Full)

(89) St Jacques PJ, Patel N, Higgins MS. Improving anesthesiologist performance through profiling and incentives. Journal of Clinical Anesthesia 2004; 16(7):523-528.

(90) Zivin JG, Pfaff ASP. To err on humans is not benign - Incentives for adoption of medical error-reporting systems. Journal of Health Economics 2004; 23(5):935-949.

(91) Beersen N, Redekop WK, de Bruijn JHB, Theuvenet PJ, Berg M, Klazinga NS. Quality based social insurance coverage and payment of the application of a high cost medical therapy: the case of spinal cord stimulation for chronic non-oncologic pain in The Netherlands. Health Policy 2005; 71(1):107-115.

(92) Feldman MD, Arean PA, Ong MK, Lee DL, Feldmann S. Incentives for primary care providers to participate in a collaborative care program for depression. Psychiatric Services 2005; 56(11):1344-1346.

(93) Gandjour A, Lauterbach KW. How much does it cost to change the behavior of health professionals? A mathematical model and an application to academic detailing. Medical Decision Making 2005; 25(3):341-347.

(94) Grembowski D, Paschane D, Diehr P, Katon W, Martin D, Patrick DL. Managed care, physician job satisfaction, and the quality of primary care. Journal of General Internal Medicine 2005; 20(3):271-277.

(95) Jack W. Purchasing health care services from providers with unknown altruism. Journal of Health Economics 2005; 24(1):73-93.

(96) Koffman DMM, Goetzel RZ, Anwuri VV, Shore KK, Orenstein D, LaPier T. Heart healthy and stroke free - Successful business strategies to prevent cardiovascular disease. American Journal of Preventive Medicine 2005; 29(5):113-121.

(97) Mentari EK, Deoreo PB, O'Connor AS, Love TE, Ricanati ES, Sehgal AR. Changes in Medicare reimbursement and patient-nephrologist visits, quality of care, and health-related quality of life. American Journal of Kidney Diseases 2005; 46(4):621-627.

(98) Saitto C, Marino C, Fusco D, Arca M, Perucci CA. Toward a new payment system for inpatient rehabilitation - Part II: Reimbursing providers. Medical Care 2005; 43(9):856-864.

(99) Schneider F, Menke R, Harter M, Salize HJ, Janssen B, Bergmann F et al. Are bonus systems applicable to guideline-oriented depression treatment provided by general practitioners and psychiatrists? Nervenarzt 2005; 76(3):308-+.

(100) Shepard DS, Daley MC, Beinecke RH, Hurley CL. Managed behavioral health care: Lessons from Massachusetts. Administration and Policy in Mental Health 2005; 32(4):311-319.

(101) Spertus JA, Eagle KA, Krumholz HM, Mitchell KR, Normand SLT. American College of Cardiology and American Heart Association methodology for the selection and creation of performance measures for quantifying the quality of cardiovascular care. Journal of the American College of Cardiology 2005; 45(7):1147-1156.

(102) Bachman J. Pay for performance in primary and specialty behavioral health care: Two "concept" proposals. Professional Psychology-Research and Practice 2006; 37(4):384-388.

(103) Backstrom M, Mjorndal T. A small economic inducement to stimulate increased reporting of adverse drug reactions--a way of dealing with an old problem? Eur J Clin Pharmacol 2006; 62(5):381-385.

(104) Bloche MG. Perspective - Tax preferences for nonprofits: From per se exemption to pay-for-performance. Health Affairs 2006; 25(4):W304-W307.

(105) Ginsburg PB. Recalibrating Medicare payments for inpatient care. New England Journal of Medicine 2006; 355(20):2061-2064.

(106) Paleologou V, Kontodimopoulos N, Stamouli A, Aletras V, Niakas D. Developing and testing an instrument for identifying performance incentives in the Greek health care sector. Bmc Health Services Research 2006; 6.

(107) Thomas MR, Waxmonsky JA, McGinnis GF, Barry CL. Realigning clinical and economic incentives to support depression management within a Medicaid population: The Colorado Access experience. Administration and Policy in Mental Health 2006; 33(1):26-33.

(108) Biai S, Rodrigues A, Gomes M, Ribeiro I, Sodemann M, Alves F et al. Reduced in-hospital mortality after improved management of children under 5 years admitted to hospital with malaria: randomised trial. BMJ 2007; 335(7625):862.

(109) Cotter CM. Making the case for a clinical information system: The chief information officer view. Journal of Critical Care 2007; 22(1):56-65.

(110) Harrison JP, Coppola MN. Is the quality of hospital care a function of leadership? Health Care Manag (Frederick ) 2007; 26(3):263-272.

(111) Huddle TS. The limits of objective assessment of medical practice. Theoretical Medicine and Bioethics 2007; 28(6):487-496.

(112) Ittner CD, Larcker DF, Pizzini M. Performance-based compensation in member-owned firms: An examination of medical group practices. Journal of Accounting & Economics 2007; 44(3):300-327.

(113) Martens JD, Werkhoven MJ, Severens JL, Winkens RAG. Effects of a behaviour independent financial incentive on prescribing behaviour of general practitioners. Journal of Evaluation in Clinical Practice 2007; 13(3):369-373.

(114) Pelonero AL, Johnson RL. A pay-for-performance program for behavioral health care practitioners. Psychiatric Services 2007; 58(4):442-444.

(115) Pronovost P, Thompson DA, Holzmueller CG, Dorman T, Morlock LL. Impact of the Leapfrog Group's intensive care unit physician staffing standard. Journal of Critical Care 2007; 22(2):89-96.

(116) Bottle A, Gnani S, Saxena S, Aylin P, Mainous AG, Majeed A. Association between quality of primary care and hospitalization for coronary heart disease in England: National cross-sectional study. Journal of General Internal Medicine 2008; 23(2):135-141.

(117) Boucai L, Zonszein J. Effects of quality improvement strategies for type 2 diabetes in Bronx, N.Y. Clinical Diabetes 25[4], 155-159. 2007.

Ref Type: Journal (Full)

(118) Buetow S, Bryant L. Is the rate of generic drug prescribing a fair measure for pay for performance? Disease Management & Health Outcomes 2008; 16(6):377-380.

(119) Chung KP, Lai MS, Cheng SH, Tang ST, Huang CC, Cheng AL et al. Organization-based performance measures of cancer care quality: core measure development for breast cancer in Taiwan. European Journal of Cancer Care 2008; 17(1):5-18.

(120) Greene RA, Beckman HB, Mahoney T. Beyond the efficiency index: Finding a better way to reduce overuse and increase efficiency in physician care. Health Affairs 2008; 27(4):W250-W259.

(121) Quimbo SA, Peabody JW, Shimkhada R, Woo K, Solon O. Should we have confidence if a physician is accredited? A study of the relative impacts of accreditation and insurance payments on quality of care in the Philippines. Social Science & Medicine 2008; 67(4):505-510.

(122) Scholle SH, Roski J, Adams JL, Dunn DL, Kerr EA, Dugan DP et al. Benchmarking physician performance: reliability of individual and composite measures. Am J Manag Care 2008; 14(12):833-838.

(123) Sinsky CA, Foreman-Hoffman V, Cram P. The impact of expressions of treatment efficacy and out-of-pocket expenses on patient and physician interest in osteoporosis treatment: Implications for pay-for-performance programs. Journal of General Internal Medicine 2008; 23(2):164-168.

(124) Tuerk PW, Mueller M, Egede LE. Estimating physician effects on glycemic control in the treatment of diabetes - Methods, effects sizes, and implications for treatment policy. Diabetes Care 2008; 31(5):869-873.

(125) Wright SW, Trott A, Lindsell CJ, Smith C, Gibler WB. Evidence-based emergency medicine. Creating a system to facilitate translation of evidence into standardized clinical practice: a preliminary report. Ann Emerg Med 2008; 51(1):80-6, 86.

(126) Arling G, Job C, Cooke V. Medicaid Nursing Home Pay for Performance: Where Do We Stand? Gerontologist 2009.

(127) Chung KC, Shauver MJ. Measuring quality in healthcare and its implications for pay-for-performance initiatives. Hand Clinics 2009; 25(1):71-81.

(128) Eggleston K, Bir A. Measuring selection incentives in managed care: evidence from the Massachusetts state employee insurance program. The Journal of Risk and Insurance 2009; 76(1):159-175.

(129) Gellad WF, Detsky AS, Choudhry NK. Implications of recent clinical trials on pay-for-performance. Am J Health Syst Pharm 2009; 66(9):864-867.

(130) Ham C. Chronic care in the English National Health Service: progress and challenges. Health Aff (Millwood ) 2009; 28(1):190-201.

(131) Hartzband P, Groopman J. Money and the changing culture of medicine. N Engl J Med 2009; 360(2):101-103.

(132) Hirth RA, Turenne MN, Wheeler JR, Pan Q, Ma Y, Messana JM. Provider Monitoring and Pay-for-Performance When Multiple Providers Affect Outcomes: An Application to Renal Dialysis. Health Serv Res 2009.

(133) Kaplan SH, Griffith JL, Price LL, Pawlson LG, Greenfield S. Improving the reliability of physician performance assessment: identifying the "physician effect" on quality and creating composite measures. Med Care 2009; 47(4):378-387.

(134) Lee TH, Ferris TG. Pay for performance: a work in progress. Circulation 2009; 119(23):2965-2966.

(135) Lehman R, Krumholz HM. Tight control of blood glucose in long standing type 2 diabetes. BMJ 2009; 338:b800.

(136) Lippi BM, Nobilio L, Ugolini C. Economic incentives in general practice: the impact of pay-for-participation and pay-for-compliance programs on diabetes care. Health Policy 2009; 90(2-3):140-148.

(137) Marco AP, Bottles K. Pay-for-performance. Physician Exec 2009; 35(2):34-39.

(138) Petersen LA, Woodard LD, Henderson LM, Urech TH, Pietz K. Will hypertension performance measures used for pay-for-performance programs penalize those who care for medically complex patients? Circulation 2009; 119(23):2978-2985.

(139) Rother J. A consumer perspective on physician payment reform. Health Aff (Millwood ) 2009; 28(2):w235-w237.

(140) Scholle SH, Roski J, Dunn DL, Adams JL, Dugan DP, Pawlson LG et al. Availability of data for measuring physician quality performance. Am J Manag Care 2009; 15(1):67-72.

(141) Zimmerman RK, Nowalk MP, Lin CJ, Raymund M, Fox DE, Harper JD et al. Factorial design for improving influenza vaccination among employees of a large health system. Infect Control Hosp Epidemiol 2009; 30(7):691-697.

(142) Feely J, Moriarty S, O'Connor P. Stimulating reporting of adverse drug reactions by using a fee. BMJ 1990; 300(6716):22-23.

(143) Reid GS, Robertson AJ, Bissett C, Smith J, Waugh N, Halkerston R. Cervical Screening in Perth and Kinross Since Introduction of the New Contract. British Medical Journal 1991; 303(6800):447-450.

(144) Ritchie LD, Bisset AF, Russell D, Leslie V, Thomson I. Primary and Preschool Immunization in Grampian - Progress and the 1990 Contract. British Medical Journal 1992; 304(6830):816-819.

(145) Kouides RW, Lewis B, Bennett NM, Bell KM, Barker WH, Black ER et al. A Performance-Based Incentive Program for Influenza Immunization in the Elderly. American Journal of Preventive Medicine 1993; 9(4):250-255.

(146) Bennett NM, Lewis B, Doniger AS, Bell K, Kouides R, LaForce FM et al. A Coordinated, Community-Wide Program in Monroe County, New-York, to Increase Influenza Immunization Rates in the Elderly. Archives of Internal Medicine 1994; 154(15):1741-1745.

(147) Lynch M. Effect of Practice and Patient Population Characteristics on the Uptake of Childhood Immunizations. British Journal of General Practice 1995; 45(393):205-208.

(148) Morrow RW, Gooding AD, Clark C. Improving physicians' preventive health care behavior through peer review and financial incentives. Arch Fam Med 1995; 4(2):165-169.

(149) Fairbrother G, Friedman S, Hanson KL, Butts GC. Effect of the vaccines for children program on inner-city neighborhood physicians. Archives of Pediatrics & Adolescent Medicine 1997; 151(12):1229-1235.

(150) Grady KE, Lemkau JP, Lee NR, Caddell C. Enhancing mammography referral in primary care. Preventive Medicine 1997; 26(6):791-800.

(151) Hillman AL, Ripley K, Goldfarb N, Nuamah I, Weiner J, Lusk E. Physician financial incentives and feedback: Failure to increase cancer screening in Medicaid managed care. American Journal of Public Health 1998; 88(11):1699-1701.

(152) Kouides RW, Bennett NM, Lewis B, Cappuccio JD, Barker WH, LaForce FM. Performance-based physician reimbursement and influenza immunization rates in the elderly. American Journal of Preventive Medicine 1998; 14(2):89-95.

(153) Cameron PA, Kennedy MP, Mcneil JJ. The effects of bonus payments on emergency service performance in Victoria. Medical Journal of Australia 1999; 171(5):243-246.

(154) Fairbrother G, Hanson KL, Friedman S, Butts GC. The impact of physician bonuses, enhanced fees, and feedback on childhood immunization coverage rates. American Journal of Public Health 1999; 89(2):171-175.

(155) Hillman AL, Ripley K, Goldfarb N, Weiner J, Nuamah I, Lusk E. The use of physician financial incentives and feedback to improve pediatric preventive care in Medicaid managed care. Pediatrics 1999; 104(4):931-935.

(156) Hopkins JR. Financial incentives for ambulatory care performance improvement. Jt Comm J Qual Improv 1999; 25(5):223-238.

(157) LeBaron CW, Mercer JT, Massoudi MS, Dini E, Stevenson J, Fischer WM et al. Changes in clinic vaccination coverage after institution of measurement and feedback in 4 states and 2 cities. Archives of Pediatrics & Adolescent Medicine 1999; 153(8):879-886.

(158) Schauffler HH, Brown C, Milstein A. Raising the bar: The use of performance guarantees by the Pacific Business Group on Health. Health Affairs 1999; 18(2):134-142.

(159) Woodson SB. Making the connection between physician performance and pay. Healthc Financ Manage 1999; 53(2):39-42, 44.

(160) Safran DG, Rogers WH, Tarlov AR, Inui T, Taira DA, Montgomery JE et al. Organizational and financial characteristics of health plans - Are they related to primary care performance? Archives of Internal Medicine 2000; 160(1):69-76.

(161) Sussman AJ, Fairchild DG, Coblyn J, Brennan TA. Primary care compensation at an academic medical center: A model for the mixed-payer environment. Academic Medicine 2001; 76(7):693-699.

(162) Cattaneo A, Borgnolo G, Simon G. Breastfeeding by objectives. European Journal of Public Health 2001; 11(4):397-401.

(163) Coleman T, Wynn AT, Stevenson K, Cheater F. Qualitative study of pilot payment aimed at increasing general practitioners' antismoking advice to smokers. British Medical Journal 2001; 323(7310):432-435.

(164) Fairbrother G, Hanson KL, Butts GC, Friedman S. Comparison of preventive care in Medicaid managed care and medicaid fee for service in institutions and private practices. Ambulatory Pediatrics 2001; 1(6):294-301.

(165) Shortell SM, Zazzali JL, Burns LR, Alexander JA, Gillies RR, Budetti PP et al. Implementing evidence-based medicine - The role of market pressures, compensation incentives, and culture in physician organizations. Medical Care 2001; 39(7):I62-I78.

(166) Ashworth M, Golding S, Majeed A. Prescribing indicators and their use by primary care groups to influence prescribing. Journal of Clinical Pharmacy and Therapeutics 2002; 27(3):197-204.

(167) Ashworth M, Golding S, Shephard L, Majeed A. Prescribing incentive schemes in two NHS regions: cross sectional survey. British Medical Journal 2002; 324(7347):1187-1188.

(168) Bond L, Davie G, Carlin JB, Lester R, Nolan T. Increases in vaccination coverage for children in child care, 1997 to 2000: an evaluation of the impact of government incentives and initiatives. Australian and New Zealand Journal of Public Health 2002; 26(1):58-64.

(169) Amundson G, Solberg LI, Reed M, Martini EM, Carlson R. Paying for quality improvement: compliance with tobacco cessation guidelines. Jt Comm J Qual Saf 2003; 29(2):59-65.

(170) Casalino L, Gillies RR, Shortell SM, Schmittdiel JA, Bodenheimer T, Robinson JC et al. External incentives, information technology, and organized processes to improve health care quality for patients with chronic diseases. Jama-Journal of the American Medical Association 2003; 289(4):434-441.

(171) Chung RS, Chernicoff HO, Nakao KA, Nickel RC, Legorreta AP. A quality-driven physician compensation model: four-year follow-up study. J Healthc Qual 2003; 25(6):31-37.

(172) Larsen DL, Cannon W, Towner S. Longitudinal assessment of a diabetes care management system in an integrated health network. J Manag Care Pharm 2003; 9(6):552-558.

(173) McMenamin SB, Schauffler HH, Shortell SM, Rundall TG, Gillies RR. Support for smoking cessation interventions in physician organizations - Results from a National Study. Medical Care 2003; 41(12):1396-1406.

(174) Roski J, Jeddeloh R, An L, Lando H, Hannan P, Hall C et al. The impact of financial incentives and a patient registry on preventive care quality: increasing provider adherence to evidence-based smoking cessation practice guidelines. Preventive Medicine 2003; 36(3):291-299.

(175) Armour BS, Friedman C, Pitts MM, Wike J, Alley L, Etchason J. The influence of year-end bonuses on colorectal cancer screening. Am J Managed Care 2004; 10(9):617-624.

(176) Ashworth M, Lea R, Gray H, Rowlands G, Gravelle H, Majeed A. How are primary care organizations using financial incentives to influence prescribing? Journal of Public Health 2004; 26(1):48-51.

(177) Berthiaume JT, Tyler PA, Ng-Osorio J, LaBresh KA. Aligning financial incentives with "get with the guidelines" to improve cardiovascular care. Am J Managed Care 2004; 10(7):501-504.

(178) Greene RA, Beckman H, Chamberlain J, Partridge G, Miller M, Burden D et al. Increasing adherence to a community-based guideline for acute sinusitis through education, physician profiling, and financial incentives. Am J Managed Care 2004; 10(10):670-678.

(179) Hippisley-Cox J, O'Hanlon S, Coupland C. Association of deprivation, ethnicity, and sex with quality indicators for diabetes: population based survey of 53 000 patients in primary care. British Medical Journal 2004; 329(7477):1267-1269.

(180) Keating NL, Landrum MB, Landon BE, Ayanian JZ, Borbas C, Robert WF et al. The influence of physicians' practice management strategies and financial arrangements on quality of care among patients with diabetes. Medical Care 2004; 42(9):829-839.

(181) Li R, Simon J, Bodenheimer T, Gillies RR, Casalino L, Schmittdiel J et al. Organizational factors affecting the adoption of diabetes care management process in physician organizations. Diabetes Care 2004; 27(10):2312-2316.

(182) McMenamin SB, Schmittdiel J, Halpin HA, Gillies R, Rundall TG, Shortell SA. Health promotion in physician organizations - Results from a national study. American Journal of Preventive Medicine 2004; 26(4):259-264.

(183) Schmittdiel J, McMenamin SB, Halpin HA, Gillies RR, Bodenheimer T, Shortell SM et al. The use of patient and physician reminders for preventive services: results from a National Study of Physician Organizations. Preventive Medicine 2004; 39(5):1000-1006.

(184) Wickizer TM, Franklin G, Gluck JV, Fulton-Kehoe D. Improving quality through identifying inappropriate care: THe use of guideline-based utilization review protocols in the Washington state workers' compensation system. Journal of Occupational and Environmental Medicine 2004; 46(3):198-204.

(185) Ashworth M, Armstrong D, de Freitas J, Boullier G, Garforth J, Virji A. The relationship between income and performance indicators in general practice: a cross-sectional study. Health Serv Manage Res 2005; 18(4):258-264.

(186) Beaulieu ND, Horrigan DR. Putting smart money to work for quality improvement. Health Services Research 2005; 40(5):1318-1334.

(187) Harries AD, Salaniponi FM, Nunn RR, Raviglione M. Performance-related allowances within the Malawi National Tuberculosis Control Programme. International Journal of Tuberculosis and Lung Disease 2005; 9(2):138-144.

(188) Majeed A, Williams J, De Lusignan S, Chan T. Management of heart failure in primary care after implementation of the National Service Framework for Coronary Heart Disease: a cross-sectional study. Public Health 2005; 119(2):105-111.

(189) May EL. Take the lead or take your chances: engaging physicians in pay-for-performance. Healthc Exec 2005; 20(2):24-28.

(190) Pourat N, Rice T, Tai-Seale M, Bolan G, Nihalani J. Association between physician compensation methods and delivery of guideline-concordant STD care: Is there a link? Am J Managed Care 2005; 11(7):426-432.

(191) Looking at lessons on quality from the Medicare pay-for-performance hospital demonstration. Qual Lett Healthc Lead 2005; 17(10):2-13, 1.

(192) Aligning incentives to promote quality care: lessons from pay-for-performance initiatives. Qual Lett Healthc Lead 2005; 17(12):2-7, 1.

(193) Rosenthal MB, Frank RG, Li ZH, Epstein AM. Early experience with pay-for-performance - From concept to practice. Jama-Journal of the American Medical Association 2005; 294(14):1788-1793.

(194) Shenkman E, Tian LL, Nackashi J, Schatz D. Managed care organization characteristics and outpatient specialty care use among children with chronic illness. Pediatrics 2005; 115(6):1547-1554.

(195) Sperl-Hillen JM, O'Connor PJ. Factors driving diabetes care improvement in a large medical group: Ten years of progress. Am J Managed Care 2005; 11(5):S177-S185.

(196) Bailit Health Purchasing LLC. Incentives and rewards best practices primer: Lessons learned from early pilots. 2006. The Leapfrog Group.

Ref Type: Report

(197) Doran T, Fullwood C, Gravelle H, Reeves D, Kontopantelis E, Hiroeh U et al. Pay-for-performance programs in family practices in the United Kingdom. New England Journal of Medicine 2006; 355(4):375-384.

(198) Ettner SL, Thompson TJ, Stevens MR, Mangione CM, Kim C, Steers WN et al. Are physician reimbursement strategies associated with processes of care and patient satisfaction for patients with diabetes in managed care? Health Services Research 2006; 41(4):1221-1241.

(199) Francis DO, Beckman H, Chamberlain J, Partridge G, Greene RA. Introducing a multifaceted intervention to improve the management of otitis media: How do pediatricians, internists, and family physicians respond? American Journal of Medical Quality 2006; 21(2):134-143.

(200) Grossbart SR. What's the return? Assessing the effect of "pay-for-performance" initiatives on the quality of care delivery. Medical Care Research and Review 2006; 63(1):29S-48S.

(201) Healy J, Sharman E, Lokuge B. Australia: Health system review. Health Systems in Transition 2006; 8(5):1-158.

(202) Jaiveer PK, Jaiveer S, Jujjavarapu SB, Morrissey J, White J, Gadsby R et al. Improvements in clinical diabetes care in the first year of the new General Medical Services contract in the UK. The British Journal of Diabetes and Vascular Disease 6, 45-48. 2006.

Ref Type: Journal (Full)

(203) Levin-Scherz J, DeVita N, Timbie J. Impact of pay-for-performance contracts and network registry on diabetes and asthma HEDIS (R) measures in an integrated delivery network. Medical Care Research and Review 2006; 63(1):14S-28S.

(204) McLean G, Sutton M, Guthrie B. Deprivation and quality of primary care services: evidence for persistence of the inverse care law from the UK Quality and Outcomes Framework. Journal of Epidemiology and Community Health 2006; 60(11):917-922.

(205) Pines JM. Profiles in patient safety: Antibiotic timing in pneumonia and pay-for-performance. Academic Emergency Medicine 2006; 13(7):787-790.

(206) Ramsay SE, Whincup PH, Lawlor DA, Papacosta O, Lennon LT, Thomas MC et al. Secondary prevention of coronary heart disease in older patients after the national service framework: population based study. British Medical Journal 2006; 332(7534):144-145.

(207) Reiter KL, Nahra TA, Alexander JA, Wheeler JR. Hospital responses to pay-for-performance incentives. Health Serv Manage Res 2006; 19(2):123-134.

(208) Reschovsky JD, Hadley J, Landon BE. Effects of compensation methods and physician group structure on physicians' perceived incentives to alter services to patients. Health Services Research 2006; 41(4):1200-1220.

(209) Rittenhouse DR, Robinson JC. Improving quality in Medicaid - The use of care management processes for chronic illness and preventive care. Medical Care 2006; 44(1):47-54.

(210) Sigfrid LA, Turner C, Crook D, Ray S. Using the UK primary care Quality and Outcomes Framework to audit health care equity: preliminary data on diabetes management. Journal of Public Health 2006; 28(3):221-225.

(211) Simpson CR, Hannaford PC, Lefevre K, Williams D. Effect of the UK incentive-based contract on the management of patients with stroke in primary care. Stroke 2006; 37(9):2354-2360.

(212) Srirangalingam U, Sahathevan SK, Lasker SS, Chowdhury TA. Changing pattern of referral to a diabetes clinic following implementation of the new UK GP contract. British Journal of General Practice 2006; 56(529):624-626.

(213) Strong M, Maheswaran R, Radford J. Socioeconomic deprivation, coronary heart disease prevalence and quality of care: a practice-level analysis in Rotherham using data from the new UK general practitioner Quality and Outcomes Framework. Journal of Public Health 2006; 28(1):39-42.

(214) Sutton M, McLean G. Determinants of primary medical care quality measured under the new UK contract: cross sectional study. British Medical Journal 2006; 332(7538):389-390.

(215) Trisolini M, Pope G, Kautter J, Aggarwal J. Medicare physician group practices: innovations in quality and efficiency. 971. 2006. The Commonwealth Fund.

Ref Type: Report

(216) Wang YY, O'Donnell CA, Mackay DF, Watt GCM. Practice size and quality attainment under the new GMS contract: a cross-sectional analysis. British Journal of General Practice 2006; 56(532):830-835.

(217) Whalley D, Bojke C, Gravelle H, Sibbald B. GP job satisfaction in view of contract reform: a national survey. Br J Gen Pract 2006; 56(523):87-92.

(218) Williams PH, De Lusignan S. Does a higher 'quality points' score mean better care in stroke? An audit of general practice medical records. Inform Prim Care 2006; 14(1):29-40.

(219) Williams TR, Raube K, Damberg CL, Mardon RE. Pay for performance: its influence on the use of IT in physician organizations. J Med Pract Manage 2006; 21(5):301-306.

(220) Wright J, Martin D, Cockings S, Polack C. Overall Quality of Outcomes Framework scores lower in practices in deprived areas. British Journal of General Practice 2006; 56(525):277-279.

(221) Ashworth M, Seed P, Armstrong D, Durbaba S, Jones R. The relationship between social deprivation and the quality of primary care: a national survey using indicators from the UK Quality and Outcomes Framework. British Journal of General Practice 2007; 57(539):441-448.

(222) Ashworth M, Lloyd D, Smith RS, Wagner A, Rowlands G. Social deprivation and statin prescribing: a cross-sectional analysis using data from the new UK general practitioner 'Quality and Outcomes Framework'. Journal of Public Health 2007; 29(1):40-47.

(223) Campbell S, Reeves D, Kontopantelis E, Middleton E, Sibbald B, Roland M. Quality of primary care in England with the introduction of pay for performance. New England Journal of Medicine 2007; 357(2):181-190.

(224) Carey IM, DeWilde S, Harris T, Whincup PH, Cook DG. Spurious trends in coronary heart disease incidence: unintended consequences of the new GP contract? British Journal of General Practice 2007; 57(539):486-489.

(225) Casale AS, Paulus RA, Selna MJ, Doll MC, Bothe AE, McKinley KE et al. "ProvenCare(SM)" a provider-driven pay-for-performance program for acute episodic cardiac surgical core. Annals of Surgery 2007; 246(4):613-623.

(226) Coleman K, Reiter KL, Fulwiler D. The impact of pay-for-performance on diabetes care in a large network of community health centers. Journal of Health Care for the Poor and Underserved 2007; 18(4):966-983.

(227) Coleman T, Lewis S, Hubbard R, Smith C. Impact of contractual financial incentives on the ascertainment and management of smoking in primary care. Addiction 2007; 102(5):803-808.

(228) Downing A, Rudge G, Cheng Y, Tu YK, Keen J, Gilthorpe MS. Do the UK government's new Quality and Outcomes Framework (QOF) scores adequately measure primary care performance? A cross-sectional survey of routine healthcare data. Bmc Health Services Research 2007; 7.

(229) Felt-Lisk S, Gimm G, Peterson S. Making pay-for-performance work in Medicaid. Health Affairs 2007; 26(4):W516-W527.

(230) Gene-Badia J, Escaramis-Babiano G, Sans-Corrales M, Sampietro-Colom L, Aguado-Menguy F, Cabezas-Pena C et al. Impact of economic incentives on quality of professional life and on end-user satisfaction in primary care. Health Policy 2007; 80(1):2-10.

(231) Gilmore AS, Zhao YX, Kang N, Ryskina KL, Legorreta AP, Taira DA et al. Patient outcomes and evidence-based medicine in a preferred provider organization setting: A six-year evaluation of a physician pay-for-performance program. Health Services Research 2007; 42(6):2140-2159.

(232) Glickman SW, Ou FS, Delong ER, Roe MT, Lytle BL, Mulgund J et al. Pay for performance, quality of care, and outcomes in acute myocardial infarction. Jama-Journal of the American Medical Association 2007; 297(21):2373-2380.

(233) Gray J, Millett C, Saxena S, Netuveli G, Khunti K, Majeed A. Ethnicity and quality of diabetes care in a health system with universal coverage: Population-based cross-sectional survey in primary care. Journal of General Internal Medicine 2007; 22(9):1317-1320.

(234) Gulliford MC, Ashworth M, Robotham D, Mohiddin A. Achievement of metabolic targets for diabetes by English primary care practices under a new system of incentives. Diabetic Medicine 2007; 24(5):505-511.

(235) Halanych JH, Safford MM, Keys WC, Person SD, Shikany JM, Kim YI et al. Burden of comorbid medical conditions and quality of diabetes care. Diabetes Care 2007; 30(12):2999-3004.

(236) Helm C, Holladay CL, Tortorella FR. The performance management system: Applying and evaluating a pay-for-performance initiative. Journal of Healthcare Management 2007; 52(1):49-62.

(237) Heneghan C, Perera R, Mant D, Glasziou P. Hypertension guideline recommendations in general practice: awareness, agreement, adoption, and adherence. British Journal of General Practice 2007; 57(545):948-952.

(238) Hughes E. Payment by results--a model for other diabetes healthcare systems? Prim Care Diabetes 2007; 1(2):111-113.

(239) Kautter J, Pope GC, Trisolini M, Grund S. Medicare Physician Group Practice demonstration design: Quality and efficiency pay-for-performance. Health Care Financing Review 2007; 29(1):15-29.

(240) Lindenauer PK, Remus D, Roman S, Rothberg MB, Benjamin EM, Ma A et al. Public reporting and pay for performance in hospital quality improvement. New England Journal of Medicine 2007; 356(5):486-496.

(241) Mandel KE, Kotagal UR. Pay for performance alone cannot drive quality. Archives of Pediatrics & Adolescent Medicine 2007; 161(7):650-655.

(242) McCarlie J, Reid E, Brady AJB. Audit of the new GMS contract Quality and Outcomes Framework: Raising standards in CHD. The British Journal of Cardiology 14, 117-120. 2007.

Ref Type: Journal (Full)

(243) McLean G, Guthrie B, Sutton M. Differences in the quality of primary medical care for CVD and diabetes across the NHS: evidence from the quality and outcomes framework. Bmc Health Services Research 2007; 7.

(244) McLean G, Guthrie B, Sutton M. Differences in the quality of primary medical care services by remoteness from urban settlements. Quality & Safety in Health Care 2007; 16(6):446-449.

(245) Mehrotra A, Pearson SD, Coltin KL, Kleinman KP, Singer JA, Rabson B et al. The response of physician groups to P4P incentives. Am J Manag Care 2007; 13(5):249-255.

(246) Millett C, Gray J, Saxena S, Netuveli G, Khunti K, Majeed A. Ethnic disparities in diabetes management and pay-for-performance in the UK: The Wandsworth prospective diabetes study. Plos Medicine 2007; 4(6):1087-1093.

(247) Millett C, Gray J, Saxena S, Netuveli G, Majeed A. Impact of a pay-for-performance incentive on support for smoking cessation and on smoking prevalence among people with diabetes. Canadian Medical Association Journal 2007; 176(12):1705-1710.

(248) Millett C, Car J, Eldred D, Khunti K, Mainous AG, Majeed A. Diabetes prevalence, process of care and outcomes in relation to practice size, caseload and deprivation: national cross-sectional study in primary care. Journal of the Royal Society of Medicine 2007; 100(6):275-283.

(249) Nalli GA, Scanlon DP, Libby D. Developing a performance-based incentive program for hospitals: a case study from Maine. Health Aff (Millwood ) 2007; 26(3):817-824.

(250) O'Malley AS, Pham HH, Reschovsky JD. Predictors of the growing influence of clinical practice guidelines. Journal of General Internal Medicine 2007; 22(6):742-748.

(251) Patel PH, Siemons D, Shields MC. Proven methods to achieve high payment for performance. J Med Pract Manage 2007; 23(1):5-11.

(252) Rosenthal MB, Camillus J. How four purchasers designed and implemented quality-based purchasing activities. 2007. Agency for Healthcare Research and Quality.

Ref Type: Report

(253) Saxena S, Car J, Eldred D, Soljak M, Majeed A. Practice size, caseload, deprivation and quality of care of patients with coronary heart disease, hypertension and stroke in primary care: national cross-sectional study. Bmc Health Services Research 2007; 7.

(254) Shohet C, Yelloly J, Bingham P, Lyratzopoulos G. The association between the quality of epilepsy management in primary care, general practice population deprivation status and epilepsy-related emergency hospitalisations. Seizure-European Journal of Epilepsy 2007; 16(4):351-355.

(255) Simon JS, Rundall TG, Shortell SM. Adoption of order entry with decision support for chronic care by physician organizations. Journal of the American Medical Informatics Association 2007; 14(4):432-439.

(256) Simpson CR, Hannaford PC, McGovern M, Taylor MW, Green PN, Lefevre K et al. Are different groups of patients with stroke more likely to be excluded from the new UK general medical services contract? A cross-sectional retrospective analysis of a large primary care population. Bmc Family Practice 2007; 8.

(257) Smith AL. Merging P4P and disease management: How do you know which one is working? Journal of Managed Care Pharmacy 2007; 13(2):S7-S10.

(258) Steel N, Maisey S, Clark A, Fleetcroft R, Howe A. Quality of clinical primary care and targeted incentive payments: an observational study. Br J Gen Pract 2007; 57(539):449-454.

(259) Tahrani AA, McCarthy M, Godson J, Taylor S, Slater H, Capps N et al. Diabetes care and the new GMS contract: the evidence for a whole county. British Journal of General Practice 2007; 57(539):483-485.

(260) Ting HH, Galvin RS, Krumholz HM, Petersen LA, Block PC. Do economic incentives improve quality of health care? Implications for pay-for-performance. ACC Cardiosource Review Journal 16[7], 22-25. 2007.

Ref Type: Journal (Full)

(261) Twardella D, Brenner H. Effects of practitioner education, practitioner payment and reimbursement of patients' drug costs on smoking cessation in primary care: a cluster randomised trial. Tobacco Control 2007; 16(1):15-21.

(262) Young GJ, Meterko M, Beckman H, Baker E, White B, Sautter KM et al. Effects of paying physicians based on their relative performance for quality. Journal of General Internal Medicine 2007; 22(6):872-876.

(263) An LC, Bluhm JH, Foldes SS, Alesci NL, Klatt CM, Center BA et al. A randomized trial of a pay-for-performance program targeting clinician referral to a state tobacco quitline. Archives of Internal Medicine 2008; 168(18):1993-1999.

(264) Ashworth M, Medina J, Morgan M. Effect of social deprivation on blood pressure monitoring and control in England: a survey of data from the quality and outcomes framework. BMJ 2008; 337:a2030.

(265) Bhattacharyya T, Mehta P, Freiberg AA. Hospital characteristics associated with success in a pay-for-performance program in orthopaedic surgery. Journal of Bone and Joint Surgery-American Volume 2008; 90A(6):1240-1243.

(266) Chang FC, Hu TW, Lin M, Yu PT, Chao KY. Effects of financing smoking cessation outpatient services in Taiwan. Tobacco Control 2008; 17(3):183-189.

(267) Cupples ME, Byrne MC, Smith SM, Leathem CS, Murphy AW. Secondary prevention of cardiovascular disease in different primary healthcare systems with and without pay-for-performance. Heart 2008; 94(12):1594-1600.

(268) Doran T, Fullwood C, Reeves D, Gravelle H, Roland M. Exclusion of patients from pay-for-performance targets by english physicians. New England Journal of Medicine 2008; 359(3):274-284.

(269) Doran T. Lessons from early experience with pay for performance. Disease Management & Health Outcomes 2008; 16(2):69-77.

(270) Fleetcroft R, Steel N, Cookson R, Howe A. "Mind the gap!" Evaluation of the performance gap attributable to exception reporting and target thresholds in the new GMS contract: National database analysis. Bmc Health Services Research 2008; 8.

(271) Gravelle H, Sutton M, Ma A. Doctor behaviour under a pay for performance contract: Further evidence from the quality and outcomes framework. 34, 1-31. 2008. The University of York, Centre for Health Economics. CHE Research Paper.

Ref Type: Report

(272) Greenberg MR, Weinstock M, Fenimore DG, Sierzega GM. Emergency department tobacco cessation program: staff participation and intervention success among patients. J Am Osteopath Assoc 2008; 108(8):391-396.

(273) Gross R, Elhaynay A, Friedman N, Buetow S. Pay-for-performance programs in P4P programs Israeli sick funds. J Health Organ Manag 2008; 22(1):23-35.

(274) Herrin J, Nicewander D, Ballard DJ. The effect of health care system administrator pay-for-performance on quality of care. Jt Comm J Qual Patient Saf 2008; 34(11):646-654.

(275) Karve AM, Ou FS, Lytle BL, Peterson ED. Potential unintended financial consequences of pay-for-performance on the quality of care for minority patients. American Heart Journal 2008; 155(3):571-576.

(276) Kirschner K, Braspenning J, Batenburg J, Van de Rijt D, Muijers P, Van Everdingen C et al. Value for money: een model voor honoreren van kwaliteit in de huisartsenpraktijk. Project Transparantie Huisartsenzorg (Fase 2). 2008. Nijmegen, Wetenschappelijk Instituut Kwaliteit Gezondheidszorg, UMC St Radboud.

Ref Type: Report

(277) MacBride-Stewart SP, Elton R, Walley T. Do quality incentives change prescribing patterns in primary care? An observational study in Scotland. Family Practice 2008; 25(1):27-32.

(278) McGovern MP, Boroujerdi MA, Taylor MW, Williams DJ, Hannaford PC, Lefevre KE et al. The effect of the UK incentive-based contract on the management of patients with coronary heart disease in primary care. Family Practice 2008; 25(1):33-39.

(279) McGovern MP, Williams DJ, Hannaford PC, Taylor MW, Lefevre KE, Boroujerdi MA et al. Introduction of a new incentive and target-based contract for family physicians in the UK: good for older patients with diabetes but less good for women? Diabetic Medicine 2008; 25(9):1083-1089.

(280) Millett C, Gray J, Bottle A, Majeed A. Ethnic disparities in blood pressure management in patients with hypertension after the introduction of pay for performance. Ann Fam Med 2008; 6(6):490-496.

(281) Millett C, Gray J, Wall M, Majeed A. Ethnic Disparities in Coronary Heart Disease Management and Pay for Performance in the UK. J Gen Intern Med 2008.

(282) Millett C, Netuveli G, Saxena S, Majeed A. Impact of pay for performance on ethnic disparities in intermediate outcomes for diabetes: longitudinal study. Diabetes Care 2008.

(283) Pearson SD, Schneider EC, Kleinman KP, Coltin KL, Singer JA. The impact of pay-for-performance on health care quality in Massachusetts, 2001-2003. Health Affairs 2008; 27(4):1167-1176.

(284) Rosenthal MB, de Brantes FS, Sinaiko AD, Frankel M, Robbins RD, Young S. Bridges to Excellence - Recognizing High-Quality Care: Analysis of Physician Quality and Resource Use. Am J Managed Care 2008; 14(10):670-677.

(285) Steel N, Bachmann M, Maisey S, Shekelle P, Breeze E, Marmot M et al. Self reported receipt of care consistent with 32 quality indicators: national population survey of adults aged 50 or more in England. British Medical Journal 2008; 337(7667).

(286) Tahrani AA, McCarthy M, Godson J, Taylor S, Slater H, Capps N et al. Impact of practice size on delivery of diabetes care before and after the Quality and Outcomes Framework implementation. British Journal of General Practice 2008; 58(553):576-579.

(287) Vaghela P, Ashworth M, Schofield P, Gulliford MC. Population intermediate outcomes of diabetes under pay for performance incentives in England from 2004 to 2008. Diabetes Care 2008.

(288) Weber V, Bloom F, Pierdon S, Wood C. Employing the electronic health record to improve diabetes care: A multifaceted intervention in an integrated delivery system. Journal of General Internal Medicine 2008; 23(4):379-382.

(289) Yao H, Wei X, Liu J, Zhao J, Hu D, Walley JD. Evaluating the effects of providing financial incentives to tuberculosis patients and health providers in China. International Journal of Tuberculosis and Lung Disease 2008; 12(10):1166-1172.

(290) Benavent J, Juan C, Clos J, Sequeira E, Gimferrer N, Vilaseca J. Using pay-for-performance to introduce changes in primary healthcare centres in Spain: first year results. Qual Prim Care 2009; 17(2):123-131.

(291) Bhattacharyya T, Freiberg AA, Mehta P, Katz JN, Ferris T. Measuring the report card: the validity of pay-for-performance metrics in orthopedic surgery. Health Aff (Millwood ) 2009; 28(2):526-532.

(292) Calvert M, Shankar A, McManus RJ, Lester H, Freemantle N. Effect of the quality and outcomes framework on diabetes care in the United Kingdom: retrospective cohort study. BMJ 2009; 338:b1870.

(293) Campbell SM, Reeves D, Kontopantelis E, Sibbald B, Roland M. Effects of pay for performance on the quality of primary care in England. N Engl J Med 2009; 361(4):368-378.

(294) Crawley D, Ng A, Mainous AG, III, Majeed A, Millett C. Impact of pay for performance on quality of chronic disease management by social class group in England. J R Soc Med 2009; 102(3):103-107.

(295) Damberg CL, Raube K, Teleki SS, Dela CE. Taking stock of pay-for-performance: a candid assessment from the front lines. Health Aff (Millwood ) 2009; 28(2):517-525.

(296) de Brantes FS, D'Andrea BG. Physicians respond to pay-for-performance incentives: larger incentives yield greater participation. Am J Manag Care 2009; 15(5):305-310.

(297) Foels T, Hewner S. Integrating pay for performance with educational strategies to improve diabetes care. Popul Health Manag 2009; 12(3):121-129.

(298) Gemmell I, Campbell S, Hann M, Sibbald B. Assessing workload in general practice in England before and after the introduction of the pay-for-performance contract. J Adv Nurs 2009; 65(3):509-515.

(299) Menachemi N, Struchen-Shellhorn W, Brooks RG, Simpson L. Influence of pay-for-performance programs on information technology use among child health providers: the devil is in the details. Pediatrics 2009; 123 Suppl 2:S92-S96.

(300) Mullen KJ, Frank RG, Rosenthal MB. Can you get what you pay for? Pay-for-performance and the quality of healthcare providers. 14886, 1-43. 2009. Cambridge, Massachusetts, National Bureau of Economic Research. NBER Working Paper Series.

Ref Type: Report

(301) Pedros C, Vallano A, Cereza G, Mendoza-Aran G, Agusti A, Aguilera C et al. An intervention to improve spontaneous adverse drug reaction reporting by hospital physicians: a time series analysis in Spain. Drug Saf 2009; 32(1):77-83.

(302) Pham HH, Landon BE, Reschovsky JD, Wu B, Schrag D. Rapidity and modality of imaging for acute low back pain in elderly patients. Arch Intern Med 2009; 169(10):972-981.

(303) Robinson JC, Casalino LP, Gillies RR, Rittenhouse DR, Shortell SS, Fernandes-Taylor S. Financial incentives, quality improvement programs, and the adoption of clinical information technology. Med Care 2009; 47(4):411-417.

(304) Rodriguez HP, Von Glahn T, Rogers WH, Safran DG. Organizational and market influences on physician performance on patient experience measures. Health Services Research 2009; 44(3):880-901.

(305) Rubinstein A, Rubinstein F, Botargues M, Barani M, Kopitowski K. A multimodal strategy based on pay-per-performance to improve quality of care of family practitioners in Argentina. J Ambul Care Manage 2009; 32(2):103-114.

(306) Ryan AM. Effects of the Premier hospital quality incentive demonstration on Medicare patient mortality and cost. Health Services Research 2009; 44(3):821-842.

(307) Strong M, South G, Carlisle R. The UK Quality and Outcomes Framework pay-for-performance scheme and spirometry: rewarding quality or just quantity? A cross-sectional study in Rotherham, UK. BMC Health Serv Res 2009; 9:108.

(308) Sutton M, Elder R, Guthrie B, Watt G. Record rewards: the effects of targeted quality incentives on the recording of risk factors by primary care providers. Health Econ 2009.

(309) Vina ER, Rhew DC, Weingarten SR, Weingarten JB, Chang JT. Relationship between organizational factors and performance among pay-for-performance hospitals. J Gen Intern Med 2009; 24(7):833-840.

(310) Kahn CN, Ault T, Isenstein H, Potetz L, Van Gelder S. Snapshot of hospital quality reporting and pay-for-performance under Medicare. Health Affairs 2006; 25(1):148-162.

(311) Fleetcroft R, Cookson R. Do the incentive payments in the new NHS contract for primary care reflect likely population health gains? J Health Serv Res Policy 2006; 11(1):27-31.

(312) McElduff P, Lyratzopoulos G, Edwards R, Heller RF, Shekelle P, Roland M. Will changes in primary care improve health outcomes? Modelling the impact of financial incentives introduced to improve quality of care in the UK. Quality & Safety in Health Care 2004; 13(3):191-197.

(313) Fleetcroft R, Parekh S, Steel N, Swift L, Cookson R, Howe A. Potential population health gain of the quality and outcomes framework. Report to Department of Health 2008. 1-25. 2008. University of East Anglia.

Ref Type: Report

(314) Nahra TA, Reiter KL, Hirth RA, Shermer JE, Wheeler JRC. Cost-effectiveness of hospital pay-for-performance incentives. Medical Care Research and Review 2006; 63(1):49S-72S.

(315) Curtin K, Beckman H, Pankow G, Milillo Y, Greene RA. Return on investment in pay for performance: A diabetes case study. Journal of Healthcare Management 2006; 51(6):365-374.

(316) Mason A, Walker S, Claxton K, Cookson R, Fenwick E, Sculpher M. The GMS Quality and Outcomes Framework: Are the Quality and Outcomes Framework (QOF) Indicators a Cost-Effective Use of NHS Resources? 2008. University of York.

Ref Type: Report

(317) Salize HJ, Merkel S, Reinhard I, Twardella D, Mann K, Brenner H. Cost-effective primary care-based strategies to improve smoking cessation: more value for money. Arch Intern Med 2009; 169(3):230-235.

(318) Langham S, Gillam S, Thorogood M. The Carrot, the Stick and the General-Practitioner - How Have Changes in Financial Incentives Affected Health Promotion Activity in General-Practice. British Journal of General Practice 1995; 45(401):665-668.

(319) Doran T, Fullwood C, Kontopantelis E, Reeves D. Effect of financial incentives on inequalities in the delivery of primary clinical care in England: analysis of clinical activity indicators for the quality and outcomes framework. Lancet 2008; 372(9640):728-736.

(317) Salize HJ, Merkel S, Reinhard I, Twardella D, Mann K, Brenner H. Cost-effective primary care-based strategies to improve smoking cessation: more value for money. Arch Intern Med 2009; 169(3):230-235.

(318) Langham S, Gillam S, Thorogood M. The Carrot, the Stick and the General-Practitioner - How Have Changes in Financial Incentives Affected Health Promotion Activity in General-Practice. British Journal of General Practice 1995; 45(401):665-668.

(319) Doran T, Fullwood C, Kontopantelis E, Reeves D. Effect of financial incentives on inequalities in the delivery of primary clinical care in England: analysis of clinical activity indicators for the quality and outcomes framework. Lancet 2008; 372(9640):728-736.
