# Supplementary material for: Systematic review: Effects, design choices, and context of pay-for-performance in health care
Source: BMC Health Serv Res. 2010 Aug 23;10:247. doi: 10.1186/1472-6963-10-247 (PMC2936378; doi:10.1186/1472-6963-10-247)
Supplement: Additional file 3 — The effects of P4P on clinical effectiveness. [file 1472-6963-10-247-S3.DOCX]

## The effects of P4P on clinical effectiveness

| Patient group | Cit | Study design* | | | Targets† | Type of measure‡ | | | | Effect§ | | | Range of effect size (%)\|\| |
| --- | --- | --- | --- | --- | --- | --- | --- | --- | --- | --- | --- | --- | --- |
|  |  | R | C+H | H Multi |  | S | P | IO | O | + | - | ↔ |  |
| Preventive care | | | | | | | | | | | | | |
| Children | ^[33;34;98;101; 102;105;106]^ | X | X |  | Immunization rate; well-child visit rate ($) |  | X |  |  |  |  | X | n.s. to 25.3, minus 5 to 5 |
|  | ^[106]^ | X |  |  | Screening rate ($) |  | X |  |  |  |  |  | n.s. |
| Adults | ^[104]^ |  |  | X | Cholesterol screening rate ($) |  | X |  |  | X |  |  | 3 |
| Influenza | ^[109;114]^ | X | X |  | Immunization rate ($) |  | X |  |  | X |  |  | 6.8 to 8.4 |
| Colorectal cancer | ^[105;113]^ | X |  |  | Screening rate ($) |  | X |  |  |  |  |  | n.s. |
| Breast cancer | ^[33;34;103;105; 110;113]^ | X | X |  | Screening rate ($) |  | X |  |  |  |  | X | n.s. to 2.2 |
| Cervical cancer | ^[33;34;103;105; 110]^ | X | X |  | Screening rate ($) |  | X |  |  |  |  | X | n.s. to 3.9 |
| Chlamydia | ^[33;34]^ |  | X |  | Screening rate ($) |  | X |  |  |  |  | X | minus 11 to 2 |
| General population | ^[33]^ |  | X |  | Avoidable hospitalization rate ($) (↓) |  |  |  | X |  |  |  | n.s. |
|  |  |  |  |  | Hospital readmission rate ($) (↓) |  |  |  | X |  |  | X | minus 0.3 to 0.1 |
| Acute care | | | | | | | | | | | | | |
| Myocardial infarction | ^[32]^ |  | X |  | Timely percutaneous intervention rate ($) |  | X |  |  | X |  |  | 5.4 |
|  |  |  |  |  | Timely thrombolytics rate ($) |  | X |  |  |  |  |  | n.s. |
|  | ^[32;80;81;92]^ |  | X |  | Aspirin at arrival, at discharge; beta blocker at arrival, at discharge; ACE inhibitor for LVSD; smoking cessation advice ($) |  | X |  |  |  |  | X | n.s. to 9.9 |
|  | ^[80;85;92]^ |  | X |  | Heparin use, glycoprotein IIb/IIIa inhibitor use, clopidogrel at discharge, dietary modification counseling, cardiac rehabilitation referral, timely ECG rate, timely thrombolytics rate, timely cardiac catheterization rate, in hospital mortality rate (not $) |  | X |  | X |  |  |  | n.s. |
|  | ^[92]^ |  | X |  | Lipid-lowering agent at discharge (not $) |  | X |  |  | X |  |  | 4.3 |
| Heart failure (acute) | ^[32]^ |  | X |  | Provision of discharge instructions ($) |  | X |  |  | X |  |  | 25.5 |
|  | ^[32;81]^ |  | X |  | ACE inhibitor for LVSD, smoking cessation advice ($) |  | X |  |  |  |  |  | n.s. |
|  | ^[32;80;81]^ |  | X |  | LVF assessment ($) |  | X |  |  |  |  | X | minus 2.4 to 5.1 |
|  | ^[80;85]^ |  | X |  | Mortality rate (not $) |  |  |  | X |  |  |  | n.s. |
| CABG | ^[83]^ |  |  | X | Discharged to home rate ($) |  |  |  | X | X |  |  | 10 |
|  |  |  |  |  | 30-day readmission rate; complication rate (total, pulmonary, neurologic); blood product use; ICU readmission rate; operative mortality; atrial fibrillation rate; deep sternal wound infection rate; reintubation rate; total ventilation hours ($) |  |  |  | X |  |  |  | n.s. |
| Community acquired pneumonia | ^[32;80;81]^ |  | X |  | Pneumococcal screening and/or vaccination rate, blood culture use ($) |  | X |  |  | X |  |  | 9.5 to 44.7, 3.5 |
|  | ^[32]^ |  | X |  | Smoking cessation advice ($) |  | X |  |  |  | X |  | minus 16.7 |
|  | ^[32;80;81]^ |  | X |  | Oxygenation assessment, timely antibiotics use ($) |  | X |  |  |  |  | X | minus 3.2 to 4.3 |
|  | ^[85]^ |  | X |  | 30-day mortality (not $) |  |  |  | X |  |  |  | n.s. |
| Bronchitis, pharyngitis | ^[33]^ |  | X |  | Preferred antibiotics usage rate ($) |  | X |  |  |  |  | X | minus 3.4 to 1.4 |
| Chronic care | | | | | | | | | | | | | |
| Diabetes | ^[33-35;79;87-89;93;95;99; 100;103;116]^ |  | X | X | Hba1c testing rate ($) |  | X |  |  |  |  | X | n.s. to 28.1 |
|  | ^[35;51;64;89;90;96;116]^ |  | X | X | Hba1c below-threshold rate ($) |  |  | X |  |  |  | X | n.s. to 19 |
|  | ^[88]^ |  | X |  | Hba1c below-threshold rate (not $) |  |  | X |  | X |  |  | 13.2 to 19.3 |
|  | ^[34;35;87-90;93;95;116]^ |  | X | X | Lipid or cholesterol testing rate ($) |  | X |  |  |  |  | X | minus 3 . to 25.8 |
|  | ^[35;51;64;87;89;90;116]^ |  | X | X | Lipid or cholesterol below-threshold rate ($) |  |  | X |  |  |  | X | n.s. to 24 |
|  | ^[88]^ |  | X |  | Lipid or cholesterol below-threshold rate (not $) |  |  | X |  | X |  |  | 29.9 |
|  | ^[35;89;116]^ |  |  | X | Blood pressure recording rate ($) |  | X |  |  |  |  | X | n.s. to 10 |
|  | ^[35;51;87;89;90;116]^ |  | X | X | Blood pressure below-threshold rate ($) |  |  | X |  | X |  |  | 1.6 to 18 |
|  | ^[34;35;87;89;93;95;103;116]^ |  | X | X | Nephropathy testing rate ($) |  | X |  |  |  |  | X | n.s. to 70 |
|  | ^[88]^ |  | X |  | Nephropathy testing rate (not $) |  | X |  |  | X |  |  | 10 |
|  | ^[116]^ |  |  | X | Weight recording ($) |  | X |  |  | X |  |  | 1.82 |
|  | ^[34;35;87;89;93;95;103;116]^ |  | X | X | Retinal exam rate ($) |  | X |  |  |  |  | X | n.s. to 37 |
|  | ^[33;88]^ |  | X |  | Retinal exam rate (not $) |  | X |  |  |  |  | X | n.s. to 10 |
|  | ^[35;87;116]^ |  | X | X | Foot exam rate ($) |  | X |  |  | X |  |  | 2.7 to 45.4 |
|  | ^[35;89;116]^ |  |  | X | Peripheral pulse testing rate ($) |  | X |  |  | X |  |  | 4.9 to 59 |
|  | ^[35;52;89;90; 116]^ |  | X | X | Smoking status recording ($) |  | X |  |  |  |  | X | n.s. to 51 |
|  | ^[52;89]^ |  |  | X | Smoking cessation advice ($) |  | X |  |  | X |  |  | 12 to 35.5 |
|  | ^[90;95]^ |  | X | X | Influenza vaccination ($) |  | X |  |  |  |  | X | n.s. to 15.9 |
|  | ^[90]^ |  |  | X | Pneumococcal vaccination ($) |  | X |  |  | X |  |  | 24.3 |
|  | ^[35]^ |  |  | X | Overall diabetes performance ($) | X | X | X |  | X |  |  | 6.9 to 7.5 |
| Heart failure (chronic) | ^[79]^ |  |  | X | ACE inhibitor or angiotensin receptor blocking use ($) |  | X |  |  | X |  |  | 23.4 |
| CHD | ^[35]^ |  |  | X | Blood pressure recording rate, blood pressure below threshold rate, blood pressure action rate, cholesterol recording rate, cholesterol below-threshold rate, cholesterol action rate, smoking status recording, smoking cessation advice, exercise ECG referral, aspirin prescription rate, beta blocker prescription rate ($) |  | X | X |  |  |  |  | n.s. |
|  | ^[33]^ |  | X |  | Cholesterol-lowering drug use (not $) |  | X |  |  |  |  |  | n.s. |
| Asthma | ^[33;93]^ |  | X |  | Asthma controller use, peak expiratory flow recording ($) |  | X |  |  |  |  |  | n.s. |
|  | ^[35]^ |  |  | X | Overall asthma performance ($) |  | X | X |  |  |  |  | 5.5 to 9.4 |
| Smoking cessation | ^[97;115;141]^ | X |  | X | Smoking status recording ($) |  | X |  |  | X |  |  | 7.9 to 24 |
|  | ^[96;115;141]^ | X |  | X | Smoking cessation advice ($) |  | X |  |  |  |  | X | n.s. to 21 |
|  | ^[86]^ | X |  |  | Smoking abstinence rate ($) |  |  |  | X |  |  |  | n.s. |
|  | ^[75]^ | X |  |  | Referral rate ($) |  | X |  |  | X |  |  | 6.2 |

Legend . *R indicates randomized; C+H, concurrent plus historical comparison; H Multi, historical comparison with multiple time points.

†$, incentivized; ↓, decrease intended.

‡S, structure; P, process; IO, intermediate outcome; O, outcome.

§+, positive effect; -, negative effect; ↔, conflicting.

||n.s., not significant.

ACE indicates angiotensin-converting enzyme; CABG, coronary artery bypass graft; CHD, coronary heart disease; Cit, citation; ECG, electrocardiogram; ICU, intensive care unit; LVF, left ventricular failure; LVSD, left ventricular systolic dysfunction
